# ZnEt_2_ as a Precatalyst for the Addition of Alcohols to Carbodiimides

**DOI:** 10.1021/acs.organomet.2c00372

**Published:** 2022-11-01

**Authors:** Alberto Ramos, Fernando Carrillo-Hermosilla, Rafael Fernández-Galán, David Elorriaga, Jesús Naranjo, Antonio Antiñolo, Daniel García-Vivó

**Affiliations:** † Departamento de Química Inorgánica, Orgánica y Bioquímica-Centro de Innovación en Química Avanzada (ORFEO−CINQA). Universidad de Castilla-La Mancha, Campus Universitario, E-13071Ciudad Real, Spain; ‡ Departamento de Química Orgánica e Inorgánica/IUQOEM, Universidad de Oviedo, E-33071Oviedo, Spain

## Abstract

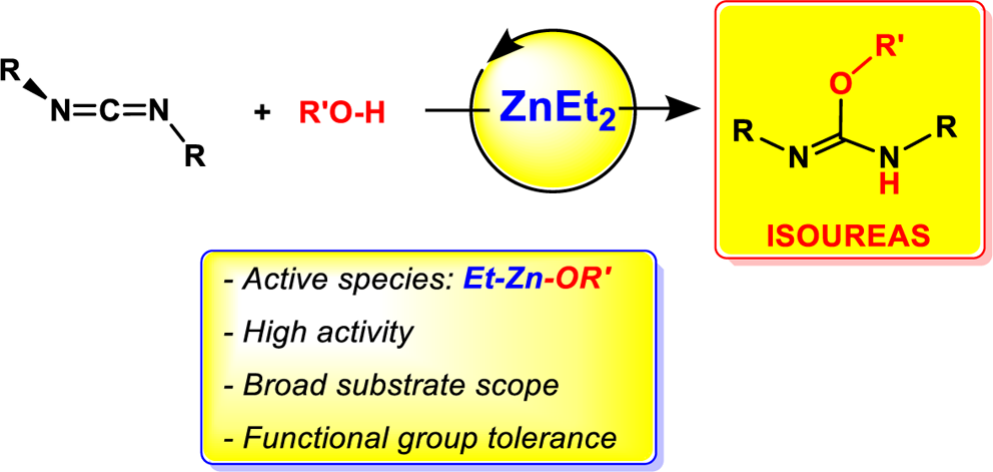

We report here the use of commercially available ZnEt_2_ as an efficient precatalyst for the addition of alcohols to carbodiimides to obtain a wide range of isoureas under mild conditions. In an initial screening using methanol and commercial carbodiimides as substrates, the bulky isourea (OMe)(NHDipp)C(NDipp) (Dipp = 2,6-*i*Pr_2_C_6_H_3_) was prepared for the first time using a catalytic method, and its structure confirmed by an X-ray diffraction analysis. Then, the efficiency of the precatalyst was tested with two carbodiimides, C(N*i*Pr)_2_ and C(N*p*-tol)_2_, toward a series of alkylic and arylic alcohols and diols, with different steric and electronic properties, including the presence of other functional groups, usually with excellent conversions, especially for the more reactive aromatic carbodiimide. Some of the new isoureas thus prepared have also been isolated and characterized. Kinetic and stoichiometric experiments allowed us to propose a plausible mechanism for these transformations.

## Introduction

**Figure 1:**
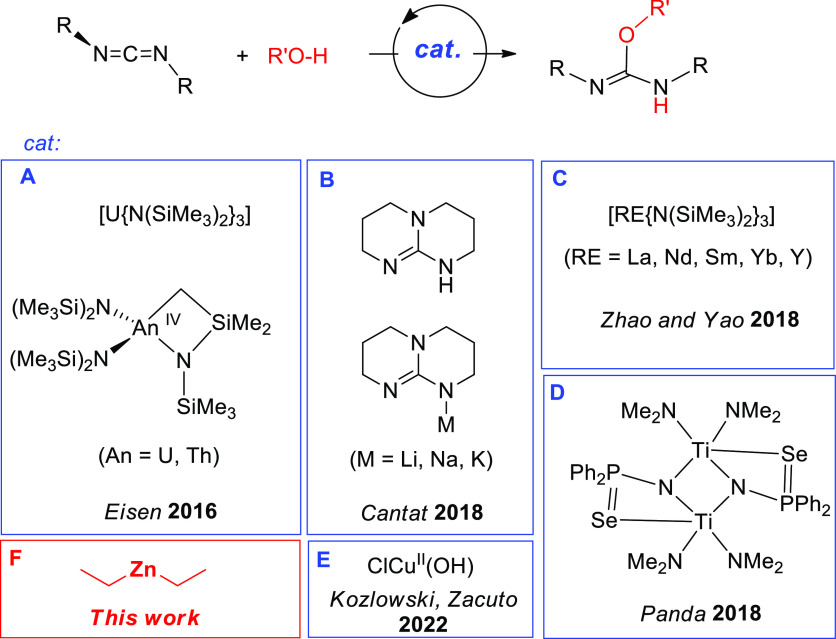
Recent examples of catalysts for the addition of alcohols to carbodiimides.

Isoureas, urea isomers of general formula (RN)C(OR^1^)(NR^2^R^3^) (R^1^ ≠ H), are very important compounds typically used in organic synthesis as alkylating reagents.^1^ Additionally, they have found applications in agriculture (stimulating plant growth),^2^ medicinal chemistry (enzyme inhibitors),^3^ physical chemistry (surfactants),^4^ and biochemistry.^5^ The starting materials traditionally used to obtain isoureas are ureas, carbodiimides, chloroformamidines, cyanamides, or organic cyanates^1c,6^ and more recently protocols, based on isonitriles have also been described,^7^ usually requiring a metal-based catalyst.^7a−7c^ From all these methods, the most straightforward and efficient in terms of atom economy is the intermolecular addition of alcohols to carbodiimides, which is also the most widely used.^1a−1c,8^ Due to the low electrophilicity of carbodiimides, these reactions often require the use of a catalyst, the most typical ones being copper(I) and copper(II) salts^1c,1e,8,9^ but also zinc(II) salts,^1c,8,9g^ in which the metal ion is believed to act as a Lewis acid interacting with the carbodiimide via the imine N atom to enhance the electrophilicity of the central carbon atom. More recently, improved state-of-the-art homogeneous catalysts have been reported for these transformations, significantly broadening the substrate scope for this reaction and adding valuable mechanistic studies as well. Thus, in 2016, Eisen and co-workers reported the first examples of actinide-based catalysts with amide ligands for the hydroalkoxylation of carbodiimides (**A** in Figure 1).^10^ In the following years, subsequent reports by the same group would expand this family of catalysts with iminato-supported actinide^11^ and hafnium^12^ complexes. In 2018, Cantat and co-workers reported 1,5,7-triazabicyclo[4.4.0]dec-5-ene and its alkali salts (Li^+^, Na^+^, K^+^) as examples of transition-metal-free catalysts for the hydroalkoxylation of carbodiimides (**B** in Figure 1), with excellent results in terms of activity for the K^+^ salt.^13^ In the same year, Zhao, Yao, and co-workers reported the use of rare-earth-metal catalysts for the first time for this transformation (**C** in Figure 1), more specifically, lanthanide and yttrium amides.^14^ Also in 2018, Panda and co-workers reported a binuclear Ti(IV) complex that turned out to be efficient catalysts for the addition of a variety of E–H bonds (E = O, C, N, P) to carbodiimides and other heterocumulenes (**D** in Figure 1).^15^ In 2022, Kozlowski, Zacuto, and co-workers used CuCl, a catalyst traditionally used for this reaction, to find an acceleration in the reaction rate in the presence of atmospheric O_2_ due to in situ formation of ClCu^II^(OH) as the active species (**E** in Figure 1).^16^

Since 2010, our group has successfully used simple, commercially available ZnEt_2_ as a precatalyst in other hydroelementation reactions involving carbodiimides and (i) amines to give guanidines (N–H addition),^17^ or (ii) terminal alkynes to give the corresponding propiolamidines (C–H addition).^18^ Additionally, in 2021, Ma, Roesky, and co-workers reported the use of this compound for the hydrophosphination (P–H addition) of carbodiimides and related heterocumulenes.^19^ In an attempt to expand the catalytic hydroelementation repertoire of ZnEt_2_, we focus now our attention on the hydroalkoxylation of carbodiimides. As mentioned above, there are scarce reports in the literature employing Zn(II) salts as Lewis-acid-type catalysts for these reactions^8a,9g^ and to the best of our knowledge, no in-depth mechanistic studies have been carried out. However, zinc alkyls have not been reported and different reaction mechanisms should be expected as well. Thus, here we report the addition of a wide range of alcohols to carbodiimides catalyzed by ZnEt_2_ (**F** in Figure 1), together with stoichiometric and kinetics experiments, allowing us to propose a plausible mechanism for this catalytic reaction.

## Results and Discussion

### Catalytic Addition of Alcohols to Carbodiimides

We start, as a model process to study, with the reaction between 1,3-diisopropylcarbodiimide (DIC) with methanol, or *t*BuOH, in order to find optimal conditions for the synthesis of isoureas (See Supporting Information, Table S1). Having established these conditions using a 5 mol % precatalyst ratio and a temperature of 60 °C, the study was extended to the reaction of different commercial carbodiimides toward methanol in the presence of ZnEt_2_, with the corresponding blank experiments (Table 1). As can be seen, DIC reacted with methanol to give essentially full conversion to isourea **1a** in 1 h at 60 °C (entry 1), whereas the control experiment only shows low conversion after protracted heating (16 h) at the same temperature (entry 2). Similar results were obtained for 1,3-dicyclohexylcarbodiimide (DCC), achieving full conversion to **2a** in 2 h at 60 °C (entry 3) with no conversion observed in the control experiment (entry 4). The bulkier 1,3-di-*tert-*butylcarbodiimide reacted sluggishly with MeOH in the presence of ZnEt_2_ and only 27% conversion to isourea **3a** was obtained after 30 h at 60 °C (entry 5). It is worth mentioning the latter isourea has been reported just once before and prepared via a stoichiometric method.^20^ Unsurprisingly, the more electrophilic 1,3-di-*p*-tolylcarbodiimide (DTC) reacted under milder conditions to give full conversion to **4a** in 1.5 h at 25 °C (entry 7), whereas in the absence of ZnEt_2_ only 64% conversion was achieved after 4 h at 60 °C (entry 8). Finally, a challenging substrate, such as the bulky 1,3-bis-(2,6-diisopropylphenyl)carbodiimide, was chosen to test the activity of ZnEt_2_ as a precatalyst. Pleasingly, under somewhat more demanding conditions (80 °C, 120 h), 97% conversion to isourea **5a** was reached (entry 9), as opposed to the blank experiment in which virtually no conversion was obtained under the same conditions (entry 10). We should note here that the latter isourea had not been prepared by catalytic methods before and, to our knowledge, it has only been reported once and prepared by a stoichiometric method (using carbodiimide and NaOMe as starting materials).^21^ Moreover, an attempted synthesis of **5a** by Eisen and co-workers using actinide-based catalysts showed no conversion after 24 h.^11e^ For that reason, we decided to isolate and fully characterize compound **5a**, which was obtained in excellent yields (89%) as a white crystalline solid.

**Table 1:** Reactivity of Different Carbodiimides toward Methanol with and without the Catalyst^a^

entry	C(NR)_2_	precatalyst	*T* (°C)	*t* (h)	conversion (%)^b^
1	R = *i*Pr	ZnEt_2_	60	1	>99
2			60	21	16
3	R = Cy	ZnEt_2_	60	2	>99
4			60	2	
5	R = *t*Bu	ZnEt_2_	60	30	27
6			60	48	
7	R = *p-*tol	ZnEt_2_	25	1.5	>99
8			60	4	64
9	R = Dipp	ZnEt_2_	80	120	97 [89]^c^
10			80	120	<1

a Reaction conditions: 0.50 mmol carbodiimide, 0.50 mmol MeOH, 0.025 mmol precatalyst (25 μL, 1.0 M solution of ZnEt_2_ in hexanes), 500 μL of C_6_D_6_, and Si(SiMe_3_)_4_ (0.01 mmol) as an internal standard.

b Based on ^1^H NMR of the reaction crude versus internal standard.

c Isolated yield from the preparative–scale reaction.

**Figure 2:**
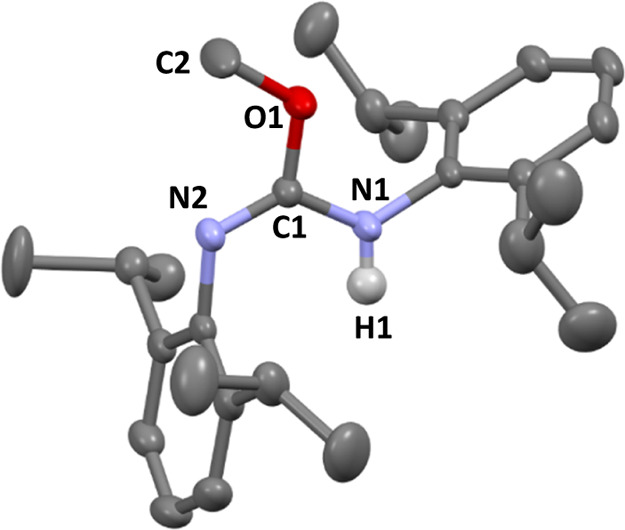
Molecular structure of **5a** with ellipsoids plotted at 30% probability and H atoms omitted for clarity (except H1, attached to N1). Selected distances (Å) and angles (°): C1–O1 1.342(2), C1–N1 1.359(2), C1–N2 1.270(2), O1–C2 1.434(2), N1–C1–N2 127.1(1), O1–C1–N1 111.0(1), N2–C1–O1 122.0(1), and C1–O1–C2 116.2(1).

An X-ray diffraction analysis of crystals of **5a** confirmed its structure unambiguously (Figure 2), displaying a planar, Y-shaped “N_2_CO” core, and an *anti*-conformation about the C=N double bond. The structural features are akin to those of the related isourea (OEt)(NHPh)C=NPh, also with an *anti*-conformation,^22^ as opposed to the *syn* isomer found in the solid state for (OPh){NH(*p*-tol)}C=N(*p*-tol), reported by Cantat and co-workers,^13^ most likely favored due to two N–H hydrogen bonds found between two molecules of isourea in the lattice. In fact, a density functional theory calculation for the *syn* and *anti*-isomers of **5a** demonstrated that the *anti*-isomer is significantly more stable (ca. 8 kcal·mol^–1^) than the *syn* counterpart, hence pointing to the retention in solution of the *anti*-conformation found in the solid-state structure. We must highlight that this trend was also observed for other isoureas derived from DIC (**1**) and DTC (**4**) with alkylic groups on the O atom, for which the *anti*-arrangement remains as the most stable one (thermodynamically), although with reduced energy gaps between *syn*/*anti* isomers. However, the opposite situation (most stable *syn* isomers) appears to be the case for most of the isoureas with aryl groups on the O atom (see the Supporting Information). Additionally, for some of the isoureas, low energy gaps between these isomers (<2 kcal·mol^–1^) are consistent with the coexistence of both isomers in solution as experimentally detected by NMR (i.e., isoureas **1d,g,i**, vide infra).

Having performed the initial screening with different carbodiimides toward methanol, we then decided to focus on the addition of a wide range of alcohols (and diols) to DIC (Table 2) and DTC (Table 3) using 5 mol % of ZnEt_2_ as a precatalyst to expand the scope of this reaction. DIC, the less electrophilic of the two carbodiimides chosen for this study, usually required temperatures of 60 °C to reach high conversions in reasonable times. Thus, reactions with EtOH and *i*PrOH proceeded to full conversion in 3 h to give isoureas **1b**,**c**, respectively. Addition of the more sterically encumbered *t*BuOH gave 88% conversion of an isomer mixture (*syn*/*anti*)^23^ of **1d** after 5 h. Because prolonged heating did not increase conversion, we assume that a thermodynamic equilibrium was reached at this temperature. We must highlight that ZnEt_2_ outperformed other catalysts reported in the literature in terms of activity and conversion for the synthesis of **1d**, such as an actinide-based catalyst reported by Eisen (17%, 24 h, 75 °C),^10^ Na-TBD reported by Cantat (58%, 5 h, 75 °C),^13^ or the rare-earth-based catalyst [La{N(SiMe_3_)_2_}_3_] (trace amounts, 24 h, 60 °C).^14^ Benzyl alcohol and 1-phenylethanol were fully consumed in 1 h to give the corresponding isoureas **1e,f**. The synthesis of **1f** is another example in which ZnEt_2_ overcame the limitations of the lanthanum catalyst [La{N(SiMe_3_)_2_}_3_],^14^ only able to produce trace amounts of **1f** after 24 h at 60 °C. Bulky 1-adamantanol reacted with DIC to give the novel isourea **1g** with 60% conversion after 6 h. Like in the reaction with *t*BuOH, further heating did not increase conversion, and NMR analysis of the reaction crude also suggested that **1g** was obtained as an isomer mixture. The reaction of phenol with DIC led to 58% conversion to **1h**. This is in line with results obtained by Cantat and co-workers with TBD-based catalysts^13^ because the synthesis of **1h** usually requires harsh reaction conditions due to strong product inhibition, as deduced from kinetic studies.^24^ Similarly, the reaction with 2,6-dimethylphenol led to 68% conversion to the novel isourea **1i**. Increasing steric bulk in phenols led to lower conversions, as in the reaction of DIC with 2,6-*tert*butylphenol, with 14% conversion to the corresponding isourea **1j**. Functional group tolerance was also tested, as in the addition of 2-(methylthio)ethanol, which rendered full conversion to **1k** in 48 h at 60 °C. This new isourea was also isolated in excellent yields as colorless oil. A pyridine-based diol was found to be also active in a double O–H addition toward 2 equiv of DIC, providing the new bisisourea **1l** quantitatively after 4 h, which was also isolated in very good yields as a white solid.

**Table 2:**
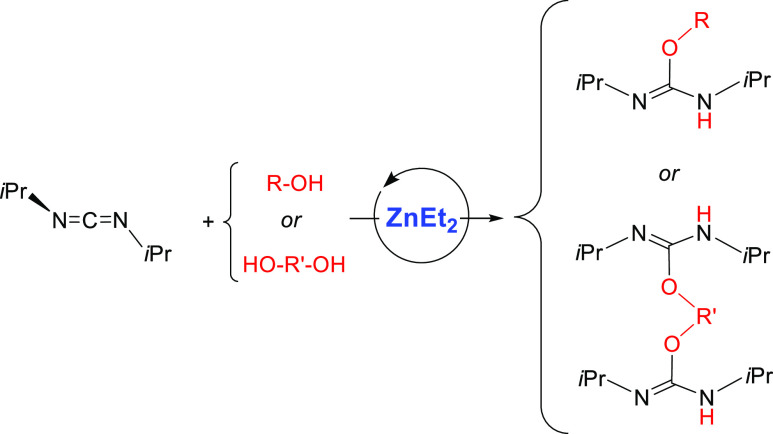

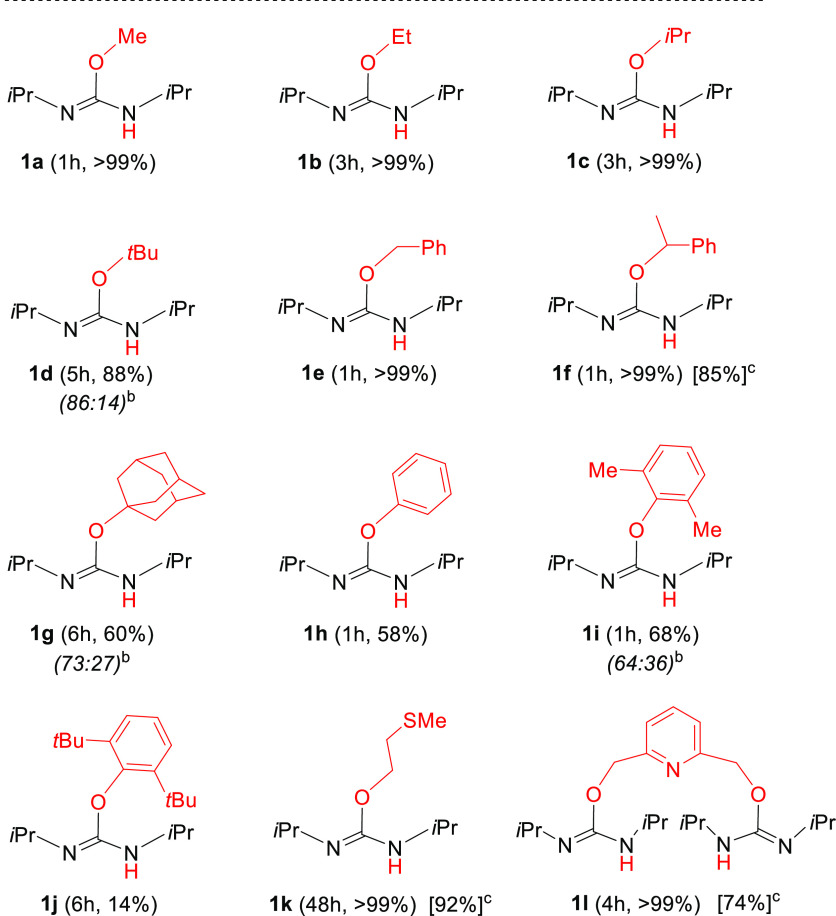
Addition of Alcohols to DIC Using ZnEt_2_ as a Precatalyst^a^

a Reaction conditions: the corresponding alcohol (0.50 mmol) or diol (0.25 mmol), 79.0 μL of DIC (0.50 mmol), 25.0 μL of a 1 M solution of ZnEt_2_ in hexane (0.025 mmol) in C_6_D_6_ (500 μL) at 60 °C, using Si(SiMe_3_)_4_ as an internal standard.

b Isomer ratio.

c Isolated yield from a preparative scale reaction.

**Table 3:**
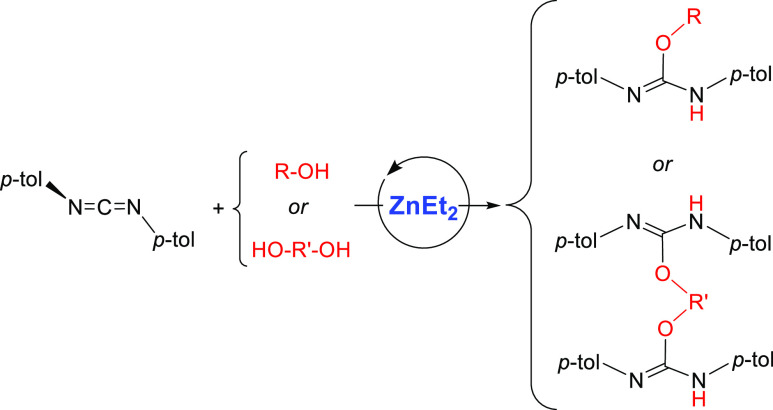

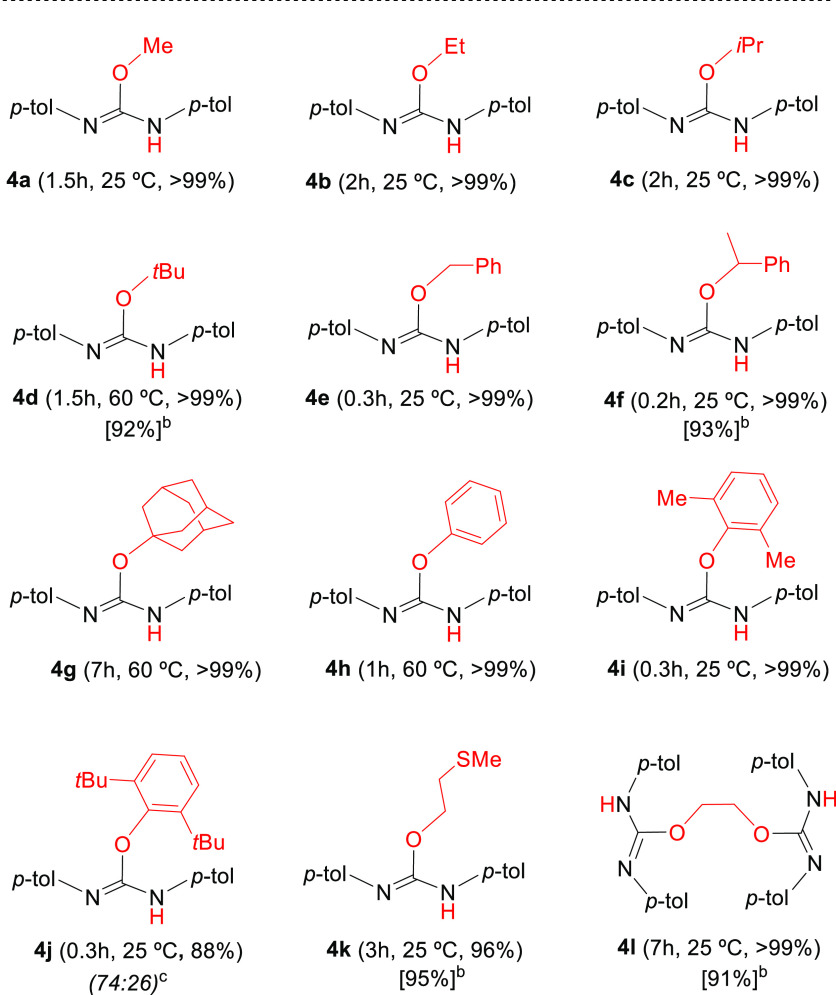
Addition of Alcohols to DTC Using ZnEt_2_ as a Precatalyst^a^

a Reaction conditions: the corresponding alcohol (0.50 mmol) or diol (0.25 mmol), 112 mg of DTC (0.50 mmol), 25.0 μL of a 1 M solution of ZnEt_2_ in hexane (0.025 mmol) in C_6_D_6_ (500 μL), using Si(SiMe_3_)_4_ as internal standard.

b Isolated yield from preparative-scale reaction.

c Isomer ratio.

Alcohol additions to the more electrophilic DTC were also carried out (Table 3). As expected, milder reaction conditions were generally required for this carbodiimide as compared to DIC. Indeed, addition of methanol, ethanol, or isopropanol proceeded in 2 h or less to give full conversion to isoureas **4a–c** at 25 °C. The reaction with bulkier *t*BuOH required somewhat harsher conditions, and full conversion was achieved after heating at 60 °C for 1.5 h. Formation of isoureas **4e,f** from benzyl alcohol and 1-phenylethanol, respectively, proceeded remarkably fast, almost upon mixing time. Moreover, isourea **4f**, reported here for the first time, was also isolated as a white solid in excellent yields. The more sterically demanding 1-adamantol required heating to 60 °C to reach total conversion to **4g** in 7 h. Phenols were also reactive toward DTC: while unsubstituted phenol needed heating at 60 °C to give **4h**, 2,6-dimethylphenol reacted essentially upon mixing with DTC to give **4i**. Likewise, bulkier 2,6-di*tert*butylphenol gave 88% conversion to isourea **4j** right after mixing all substrates, as an isomer mixture (*syn/anti*). Again, longer reaction times did not increase the conversion, suggesting a thermodynamic equilibrium between O–H addition and elimination. The presence of heteroatoms was well tolerated, as proven by the reaction with 2-(methylthio)ethanol, which gave 96% conversion to the isourea **4k** in 3 h at 25 °C. Finally, glycol was chosen to test the double O–H addition toward 2 equivalents of DTC with excellent results, as denoted by the full conversion to bisisourea **4l** under mild conditions (25 °C, 7 h). The latter two isoureas, **4k** and **4l**, have also been prepared for the first time and were isolated as white solids in excellent yields.

### Mechanistic Studies

**Figure 3:**
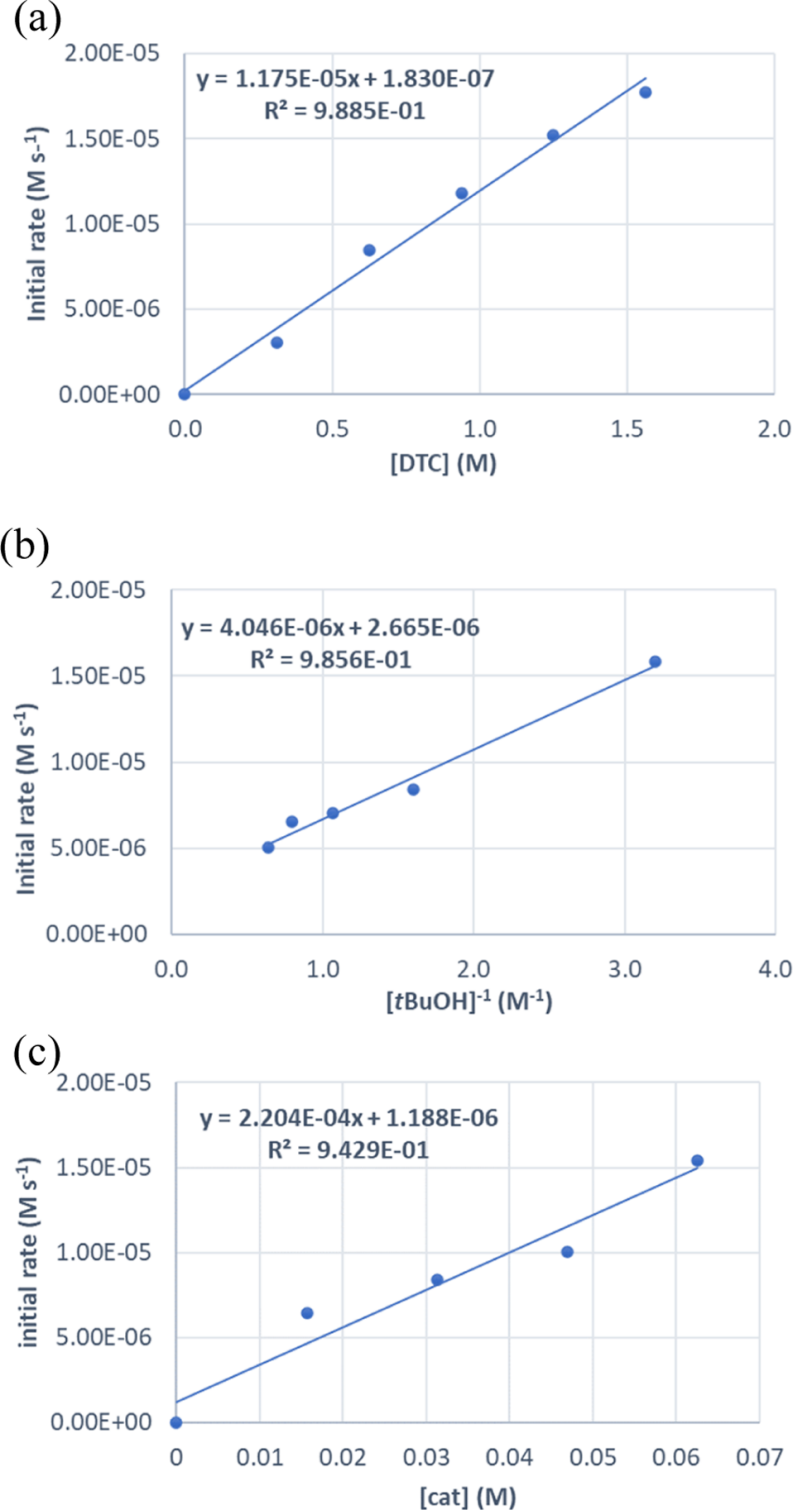
Plots of initial rate versus: (a) [DTC], (b) [*t*BuOH]^−1^, (c) [ZnEt_2_], for the formation of **4d** from *t*BuOH and DTC using ZnEt_2_ as a precatalyst.

To shed some light upon the plausible mechanism for the addition of alcohols to carbodiimides using ZnEt_2_ as a precatalyst, we decided to carry out kinetic studies. For that purpose, we chose to study the reaction between DTC and *t*BuOH to give **4d**, which proceeded slow enough at 25 °C allowing us to use the initial reaction rate method. Thus, after a series of experiments following product formation at the early stages of the reaction, by varying the concentration of each one of the substrates or precatalyst, while keeping the others constant we found a first-order dependence both on the concentration of ZnEt_2_ and the concentration of DTC and an inverse first-order dependence on the concentration of the alcohol (Figure 3).
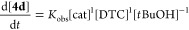


**Figure 4:**
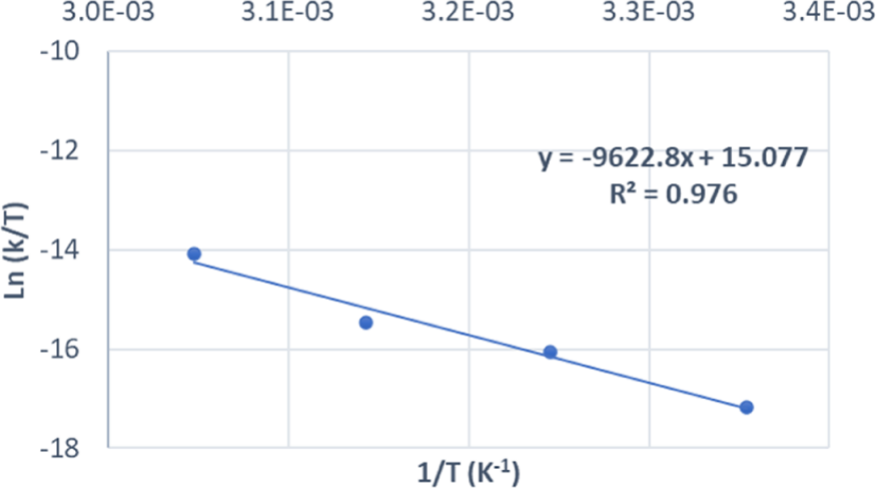
Eyring plot for the formation of **4d** from *t*BuOH and DTC using ZnEt_2_ as a precatalyst.

Experiments at different temperatures allowed us to obtain the activation parameters. Thus, an activation energy (*E*_a_) value of 83(9) KJ mol^–1^ was obtained from the Arrhenius plot (Figure S33, Supporting Information), and enthalpy (Δ*H*^‡^) and entropy (Δ*S*^‡^) of activation of 80(9) KJ mol^–1^ and -72(28) e. u. were obtained from the Eyring plot (Figure 4), the latter suggesting a highly ordered transition state in the rate-determining step of the reaction. To complete our kinetic studies, experiments employing *t*BuOD instead of *t*BuOH allowed us to obtain KIE values of 2.6(5), indicating that a step involving direct protonolysis should be the rate determining step (vide infra).

**Scheme 1:**
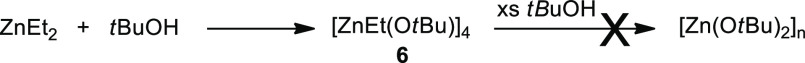
Reaction of ZnEt_2_ with *t*BuOH to Form **6**

To gain further understanding of the reaction mechanism, we also performed a series of stoichiometric experiments (Supporting Information, Figures S36–S43). In the first place, we noticed that, while monitoring the catalytic additions of *t*BuOH toward DIC and DTC to give isoureas **1d** and **4d** by ^1^H NMR, a new set of signals appeared that could be assigned to the ethyl [ca. 1.5 (triplet) and ca. 0.6 (quadruplet) ppm] and the *tert-*butoxide [ca. 1.3 ppm (singlet)] groups of the tetramer [ZnEt(O*t*Bu)]_4_ (**6**),^25^ the expected mono-protonolysis product in the reaction of ZnEt_2_ with *t*BuOH, suggesting that the excess *t*BuOH is unable to react with the second alkyl group. To confirm our proposal, a stoichiometric reaction in a preparative scale between ZnEt_2_ and *tert-*butanol was carried out, affording compound **6** in excellent yields. We also tested that the reaction product was independent of the stoichiometry employed, and even an excess *t*BuOH did not give the bisalkoxide derivative, confirming our prior observations during the catalysis (Scheme 1).^25^

These results strongly suggest that compound **6** is one of the active species in the catalytic addition of *t*BuOH to DTC, which was further confirmed after running a catalytic test with **6** as a catalyst under the same reaction conditions and obtaining >99% conversions in 1.5 h at 60 °C as well. Once established the plausible role of alkoxide derivative **6** in the reaction, we also confirmed that ZnEt_2_ did not react with equimolar amounts of DTC or DIC under the latter conditions (>1 h, 60 °C), ruling out reaction pathways involving the insertion of carbodiimides in Zn–C(alkyl) bonds. Neither did compound **6**, hinting that the insertion of carbodiimides into Zn-OR bonds is thermodynamically unfavorable. However, the addition of 1 equiv of *t*BuOH to the latter solutions at 60 °C (for solutions with DIC) or room temperature (for solutions with DTC) gave variable amounts of the isoureas **1d** (isomer mixture) and **4d**, respectively, together with the formation of the expected compound **6** in the reactions with ZnEt_2_. In all these reactions, zinc isoureato intermediates were not detected either, again evidencing the instability of these compounds. Further attempts were made to prepare these intermediates by mixing isourea **4d** with ZnEt_2_ in equimolar amounts and monitoring the reaction by ^1^H NMR. We observed, already upon mixing at room temperature, that the signals of isourea are no longer present and instead a new set of broad signals were clearly observed in the regions 7.2–6.0 and 2.2–2.1 ppm, attributed most likely to aromatic and methyl protons from *p*-tolyl groups of novel isoureato derivatives. Unfortunately, attempts to isolate and/or purify these species were unsuccessful, but we must point out that keeping these solutions at room temperature for prolonged times resulted in progressive disappearance of these intermediates to give mixtures containing **6** and DTC as the major products after 3 days, which evidences the presence of unstable zinc isoureato products that de-insert carbodiimide to give back the zinc alkoxide **6**.

**Scheme 2:**
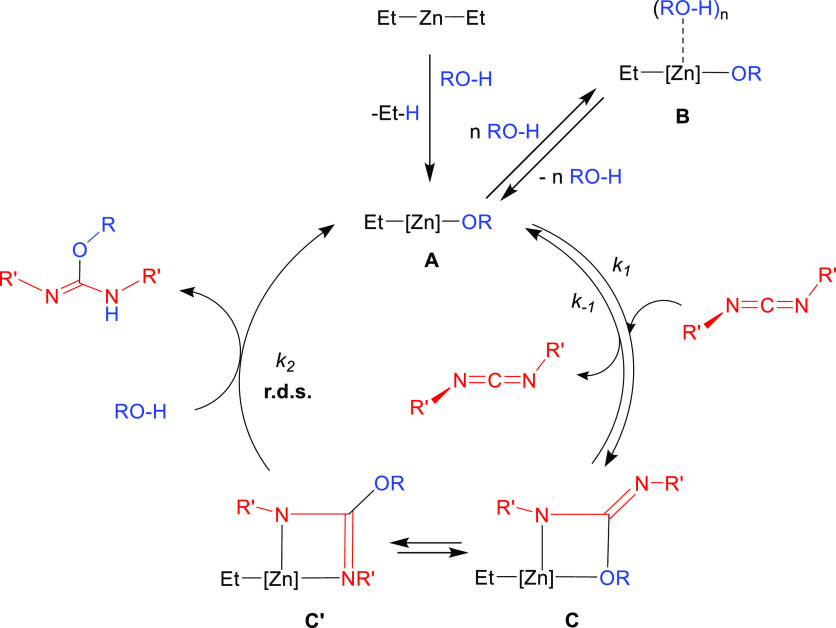
Proposed Mechanism for the Addition of Alcohols to Carbodiimides Catalyzed by ZnEt_2_. In the Scheme, [Zn] Represents One of the Reactive Centers for the Possible Aggregates of A, B, C, and C′

Combining the kinetics with the stoichiometric experiments we were able to propose a plausible reaction mechanism for the addition of alcohols to carbodiimides catalyzed by ZnEt_2_ (Scheme 2). Initially, the diethylzinc would react with one equivalent of alcohol releasing ethane to give a zinc monoalkoxide species (**A** in Scheme 2), which, based on the literature of zinc(II) alkyl-alkoxides, would most likely be a tetramer.^25,26^ A ^1^H NMR study of **6** at 60 °C (the highest temperature used in catalytic experiments), in C_6_D_6_, reveals only a slight shift of the signals of the ethyl and *tert*-butyl groups of the compound. This could indicate that the tetrameric structure is essentially maintained at this temperature, although it cannot be ruled out that the presence of an excess of alcohol and carbodiimide during the catalytic process could alter this structure. Then, [2 + 2] cycloaddition of a molecule of carbodiimide and a Zn-OR moiety of **A** would give rise to a *N,O*-isoureato intermediate (**C** in Scheme 1), which may isomerize or be in equilibrium with a more stable *N,N*′-isoureato species (**C**′ in Scheme 1). Although these intermediates could not be isolated, previously described *N,N′*-monoguanidinato,^17h^ propiolamidinato,^18^ and phosphaguanidinato^19^ zinc compounds structurally related to **C′** displayed a dimeric structure in the solid state, as evidenced by X-ray diffraction studies. Moreover, these compounds were all proposed as intermediates in the addition of amines, terminal alkynes and phosphines to carbodiimides catalyzed by ZnEt_2_. As already mentioned, the insertion of carbodiimide in **A** to give the isoureato derivatives is an equilibrium because the inverse reaction (de-insertion of carbodiimide) slowly takes place in the absence of alcohol to give back the alkoxide **A**. However, in the presence of excess *t*BuOH, protonolysis of intermediates **C** and/or **C′** would take place to generate free isourea and regenerate alkoxide **A** completing the cycle. According to the KIE analysis, this would be the rate-determining step of the reaction, which is also in good agreement with the negative value of Δ*S*^‡^ obtained in the Eyring plot, as the protonolysis of the isoureato **C**/**C′** would involve a more ordered transition state at this step. Finally, to account for the inverse first-order on the alcohol experimentally obtained, we must propose an off–cycle equilibrium involving one or more molecules of alcohol. This equilibrium might involve the coordination of one or several molecules of alcohol to Zn centers of the alkoxide derivative **A** to form adducts of the type **B**. Thus, coordination of alcohol molecules to form adducts of the type **B** would compete with the coordination and insertion of carbodiimide to give isoureatos **C**/**C′**. To experimentally prove this theory, we carried out the reaction of compound **6** toward 1 and 5 equiv of *t*BuOH and checked for significant changes in the ^1^H NMR spectra (Supporting Information, Figure S44). An upfield shift was especially noticeable in the signals due to the Et moiety (ca. 0.04 ppm) after adding 5 equiv of alcohol, which can be attributed to weak interactions between molecules of *t*BuOH and compound **6**. Similar equilibria were already proposed by Eisen and co-workers with actinide alkoxides to account for inverse first-order kinetics in alcohol in the same type of catalysis.^10,11b,11d,11e^

## Conclusions

In summary, we have expanded the catalytic repertoire of commercially available ZnEt_2_ for hydroelementation reactions proving this time its effectiveness for the addition of alcohols to carbodiimides. Using this precatalyst, we have prepared a wide range of isoureas under mild conditions, some of them synthesized for the first time. An initial screening showed that ZnEt_2_ is a very active precatalyst for the addition of MeOH to electrophilic carbodiimides such as DTC even at 25 °C and to less reactive alkylic carbodiimides, such as DIC and DCC, at 60 °C. The catalytic hydroalkoxylation of the more sterically demanding carbodiimide C(NDipp)_2_, not reported until now, was even possible under slightly harsher conditions (80 °C, 5 days). Afterward, a set of different alkylic and arylic alcohols, with different steric demands and electronic properties, were tested toward DIC and DTC. Again, ZnEt_2_ proved to be more active toward DTC, reaching very high conversions under mild conditions (25 °C) even with the most challenging and sterically demanding substrates, whereas the less electrophilic DIC required higher temperatures (60 °C) and the conversion only dropped substantially with phenols and very bulky alcohols. The presence of other functionalities on the alcohols, such as thiolates (−SR), was well tolerated by the catalyst for both carbodiimides.

Kinetic and stoichiometric experiments combined allowed us to propose a plausible mechanism for these catalytic reactions, involving an alkyl-alkoxide zinc intermediate (detected and isolated) and unstable alkyl-isoureato zinc compounds (detected by NMR). The protonolysis of the latter species turned out to be the rate-determining step of the reaction, as determined by KIE experiments. Additionally, an inverse first-order dependence was found in the alcohol concentration, suggesting an off–cycle equilibrium probably involving weak coordination of alcohol molecules to the alkoxide compound.

## Supporting Information Available

General remarks, synthetic protocols for catalytic reactions and characterization data, NMR spectra for compounds **1-6**, kinetic studies, stoichiometric reactions, crystallography, and theoretical calculations (PDF)
Cartesian coordinates of the optimized structures (XYZ)

## Supplementary Material

om2c00372_si_001.pdf

om2c00372_si_002.xyz

## Notes

CCDC 2172359 contains the supplementary crystallographic data for this paper. These data can be obtained free of charge via ww.ccdc.cam.ac.uk/data_request/cif, or by emailing data_request@ccdc.cam.ac.uk, or by contacting The Cambridge Crystallographic Data Centre, 12 Union Road, Cambridge CB2 1EZ, UK; fax: +44 1223 336033.
