# Supplementary material for: ZnEt2 as a Precatalyst for the Addition of Alcohols to Carbodiimides
Source: Organometallics. 2022 Nov 1;41(21):2949–57. doi: 10.1021/acs.organomet.2c00372 (PMC9667888; doi:10.1021/acs.organomet.2c00372)
Supplement: Supplementary file 1 — om2c00372_si_001.pdf [file om2c00372_si_001.pdf]

Supporting Information

$ZnEt_2$ as precatalyst for the addition of alcohols to carbodiimides

Alberto Ramos,*[a] Fernando Carrillo-Hermosilla,*[a] Rafael Fernández-Galán,[a] David Elorriaga,[a] Jesús Naranjo,[a] Antonio Antiñolo,[a] Daniel García-Vivó[b]

[a] *Centro de Innovación en Química Avanzada (ORFEO-CINQA). Departamento de Química Inorgánica, Orgánica y Bioquímica, Facultad de Ciencias y Tecnologías Químicas, Universidad de Castilla-La Mancha, Campus Universitario, E-13071 Ciudad Real, Spain.*

[b] *Departamento de Química Orgánica e Inorgánica/IUQOEM, Universidad de Oviedo, E-33071 Oviedo, Spain.*

*Email: fernando.carrillo@uclm.es

CONTENTS

General remarks, protocols for catalytic reaction and characterization data……….S2

NMR spectra for compounds **1-6**………………………………………………………………….S11

Kinetic studies……………………………………………………………………………………….S41

Stoichiometric experiments……………………………………………………………………….S46

Crystallography…………………………………………………………………………………….S55

Theoretical calculations…………………………………………………………………………..S57

References…………………………………………………………………………………………….S61

General remarks. All manipulations were carried out under dry nitrogen using standard Schlenk and glovebox techniques. Anhydrous solvents, purchased from the usual chemical suppliers, were stored in a glovebox with activated molecular sieves (4 Å). Mass spectroscopic analyses (electrospray ionization) were performed on a MAXIS II instrument. Microanalyses were carried out with a LECO CHNS-932 analyser. NMR spectra were recorded on Bruker 400 and 500 spectrometers in C_6D_6 at 298 K unless otherwise stated, using standard TOPSPIN 4.0 software. ^{1}H NMR and ^{13}C{^{1}H} NMR chemical shifts are referenced to residual protons or carbons in deuterated solvent. Chemical shifts (δ) are given in ppm and coupling constants (J) in Hz. All reagents were purchased from the usual commercial suppliers, except *N,N'*-di-*p*-tolylcarbodiimide (DTC), which was synthesized according to a literature procedure.[1]

General protocol for the catalytic addition of alcohols to carbodiimides. *NMR scale*: In a vial inside the glovebox, the corresponding alcohol (0.50 mmol) or diol (0.25 mmol) was added to a solution containing 0.50 mmol of carbodiimide and tetrakis(trimethyl)silane (TKS, 50.0 µL of a 0.2 M stock solution, 0.01 mmol) as internal standard in C_6D_6 (500 µL). Then, 25.0 µL of a $ZnEt_2$ solution in hexane (1 M, 0.025 mmol, 5 mol%) was added to the latter mixture, briefly stirred, and charged into an NMR tube, which was taken outside the glovebox and kept at the desired temperature in an oil bath. The reaction progress was monitored periodically by ^{1}H and ^{13}C{^{1}H} NMR spectroscopy. Known compounds were compared to previous literature. *Preparative scale*: Catalytic reactions using 5 mol% of $ZnEt_2$ and the appropriate amounts of carbodiimide and alcohol (or diol) were carried out in a vial inside the glovebox (25 ºC) or in a Schlenk with a J. Young Teflon stopper (>25 ºC) in C_6D_6 or toluene. After the time required to complete the reaction, the solvents were removed under vacuum and the product was extracted with hexane or dichloromethane (depending on the solubility), filtered through a celite plug under air and dried under vacuum. The product thus obtained (oil or white solid) was not further purified unless otherwise stated.

Table S1. Preliminary optimization tests for the catalytic addition of alcohols to carbodiimides, using $ZnEt_2$ as precatalyst and C(N*i*Pr)$_2$ and MeOH, or *t*BuOH, as substrates.[a]

Entry	ROH	Precatalyst	T (ºC)	t (h)	Conversion (%)[b]
1	R = *Me*	-	60	21	16
2	R = *Me*	1 mol%	25	24	14
3	R = *Me*	1 mol%	60	1	93
4	R = *Me*	5 mol%	60	1	>99
5	R = *t*Bu	5 mol%	60	1h	46
6	R = *t*Bu	5 mol%	60	5h	88

[a] Reaction conditions: 0.50 mmol carbodiimide, 0.50 mmol ROH, precatalyst (1.0 M solution of $ZnEt_2$ in hexanes), 500 µL of C_6D_6, and Si(SiMe$_3$)$_4$ (0.01 mmol) as an internal standard. [b] Based on ^{1}H NMR of the reaction crude vs internal standard.

Characterisation data

***N,N'*-diisopropyl-*O*-methylisourea (1a).** 79.0 µL of *N,N'*-diisopropylcarbodiimide (DIC, 0.50 mmol), 21.0 µL of MeOH (0.50 mmol), 60 ºC, 1 h, >99% conversion. ^{1}H NMR (400 MHz, C_6D_6) δ 3.72 (dsept, *J* = 7.9, 6.4 Hz, 1H, NHC*H-i*Pr), 3.64 (s, 3H, O*Me*), 3.19 (d, br, *J* = 7.9 Hz, 1H, N*H*), 3.11 (sept, *J* = 6.2 Hz, 1H, NC*H-i*Pr), 1.20 (d, *J* = 6.2 Hz, 6H, C*H$_3$-i*Pr), 0.90 (d, *J* = 6.4 Hz, 6H, C*H$_3$-i*Pr). ^{13}C{^{1}H} NMR (101 MHz, C_6D_6) δ 151.6 (s, *C*N$_2$), 52.5 (s, O*Me*), 46.5, 43.5, (2 x s, 2 x *C*H-*i*Pr), 24.9, 24.0 (2 x s, 2 x *C*H$_3$-*i*Pr). These data are consistent with previous reports.[2]

***N,N'*-diisopropyl-*O*-ethylisourea (1b).** 79.0 µL of DIC (0.50 mmol), 29.0 µL of EtOH (0.50 mmol), 60 ºC, 3 h, >99% conversion. ^{1}H NMR (500 MHz, C_6D_6) δ 4.21 (q, *J* = 7.1 Hz, 2H, C*H$_2$*-Et), 3.74 (dsept, *J* = 8.0, 6.4 Hz, 1H, NHC*H-i*Pr), 3.18 (d, br, *J* = 8.0 Hz, 1H, N*H*), 3.12 (sept, *J* = 6.2 Hz, 1H, NC*H-i*Pr), 1.20 (d, *J* = 6.2 Hz, 6H, C*H$_3$-i*Pr), 1.13 (t, *J* = 7.1 Hz, 3H, C*H$_3$*-Et), 0.92 (d, *J* = 6.4 Hz, 6H, C*H$_3$-i*Pr). ^{13}C{^{1}H} NMR (126 MHz, C_6D_6) δ 150.9 (s, *C*N$_2$), 60.6 (s, *C*H$_2$-Et), 46.5, 43.4 (2 x s, 2 x *C*H-*i*Pr), 24.9, 24.0 (2 x s, 2 x *C*H$_3$-*i*Pr), 14.7 (s, *C*H$_3$-Et). These data are consistent with previous reports.[2-3]

***N,N'*-diisopropyl-*O*-isopropylisourea (1c).** 79.0 µL of DIC (0.50 mmol), 38.5 µL of *i*PrOH (0.50 mmol), 60 ºC, 3 h, >99% conversion. ^{1}H NMR (400 MHz, C_6D_6) δ 5.31 (sept, *J* = 6.3 Hz, 1H, OC*H-i*Pr), 3.73 (dsept, *J* = 7.9, 6.4 Hz, 1H, NHC*H-i*Pr), 3.17 (br, d, *J* = 7.9 Hz, 1H, N*H*), 3.12 (sept, *J* = 6.2 Hz, 1H, NC*H-i*Pr), 1.20 (d, *J* = 6.3 Hz, 6H, C*H$_3$-i*Pr), 1.19 (d, *J* = 6.3 Hz, 6H, C*H$_3$-i*Pr), 0.92 (d, *J* = 6.4 Hz, 6H, C*H$_3$-i*Pr). ^{13}C{^{1}H} NMR (101 MHz, C_6D_6) δ 150.3 (s, *C*N$_2$), 66.5 (s, O*C*H-*i*Pr), 46.5, 43.3 (2 x s, 2 x N*C*H-

*i*Pr), 24.9, 24.0, 22.2 (3 x s, 3 x *C*H_3-*i*Pr). These data are consistent with previous reports.[2a, c]

***N,N'*-diisopropyl-*O-tert*-butylisourea (*syn*- and *anti*-1d).** 79.0 µL of DIC (0.50 mmol), 48.3 µL of *t*BuOH (0.50 mmol), 60 ºC, 5 h, 88% conversion to isomer mixture of **1d** (isomer ratio = 86:14). ^{1}H NMR (400 MHz, C_6D_6) δ 3.92 (sept, *J* = 6.4 Hz, 2H, C*H*-*i*Pr, *min*), 3.66 (dsept, *J* = 7.9, 6.4 Hz, 1H, NHC*H*-*i*Pr, *maj*), 3.11 (br, 1H, N*H*, *maj*), 3.10 (sept, *J* = 6.1 Hz, 1H, NC*H*-*i*Pr, *maj*), 2.88 (br, 1H, N*H*, *min*), 1.56 (s, 9H, C*Me*$_3$, *maj*), ca. 1.25 (br, C*H*$_3$-*i*Pr, 6H, *min*), 1.21 (s, 9H, C*Me*$_3$, *min*), 1.16 (d, *J* = 6.1 Hz, 6H, C*H*$_3$-*i*Pr, *maj*), ca. 1.00 (br, C*H*$_3$-*i*Pr, 6H, *min*), 0.92 (d, *J* = 6.4 Hz, 6H, C*H*$_3$-*i*Pr, *maj*). ^{13}C{^{1}H} NMR (101 MHz, C_6D_6) δ 149.7 (s, *C*N_2, *maj*) 148.0 (s, *C*N_2, *min*), 78.1 (s, O*C*Me$_3$, *maj*), 77.6 (s, O*C*Me$_3$, *min*), 46.9 (br, *C*H-*i*Pr, *min*), 46.6, 43.6 (2 x s, 2 x *C*H-*i*Pr, *maj*), 43.4 (br, *C*H-*i*Pr, *min*), 29.2 (s, OC*Me*$_3$, *min*), 28.8 (s, O*C*Me$_3$, *maj*), 25.5 (s, *C*H_3-*i*Pr, *min*), 24.8, 24.0 (2 x s, 2 x *C*H_3-*i*Pr, *maj*), 22.8 (s, *C*H_3-*i*Pr, *min*). These data are consistent with previous reports.[2a, c, 4]

***N,N'*-diisopropyl-*O*-benzylisourea (1e).** 79.0 µL of DIC (0.50 mmol), 51.8 µL of benzyl alcohol (0.50 mmol), 60 ºC, 1 h, >99% conversion. ^{1}H NMR (400 MHz, C_6D_6) δ 7.32 (m, 2H, *o/m*-Ph), 7.14 (m, 2H, *m/o*-Ph), 7.07 (m, 1H, *p*-Ph), 5.28 (s, 2H C*H*$_2$), 3.74 (dsept, *J* = 8.0, 6.5 Hz, 1H, C*H*-*i*Pr), 3.22 (d, *J* = 8.0 Hz, 1H N*H*), 3.13 (sept, *J* = 6.3 Hz, 1H, C*H*-*i*Pr), 1.22 (d, *J* = 6.3 Hz, 6H C*H*$_3$-*i*Pr), 0.88 (d, *J* = 6.5 Hz, 6H C*H*$_3$-*i*Pr). ^{13}C{^{1}H} NMR (101 MHz, C_6D_6) δ 150.8 (s, *C*N_2), 138.9 (s, *ipso*-Ph), 128.5, 128.2 (2 x s, *o/m*-Ph) 127.6 (s, *p*-Ph), 67.0 (s, O*C*H_2), 46.6, 43.5 (2 x s, 2 x *C*H-*i*Pr), 24.9, 24.0 (2 x s, 2 x *C*H_3-*i*Pr). These data are consistent with previous reports.[3, 5]

***N,N'*-diisopropyl-*O*-(1-phenyl)ethylisourea (1f).** 79.0 µL of DIC (0.50 mmol), 61.6 µL of 1-phenylethanol (0.50 mmol). 60 ºC, 1 h, >99% conversion. *Preparative scale*: 330 µL of DIC (2.11 mmol), 250 µL of 1-phenylethanol (2.03 mmol) in toluene (2 mL), 60 ºC, 1.5 h (colorless oil, yield: 430 mg, 85%). ^{1}H NMR (400 MHz, C_6D_6) δ 7.37 (m, 2H, *o/m*-Ph), 7.17 (m, 2H, *m/o*-Ph), 7.07 (m, 1H *p*-Ph), 6.30 (q, *J* = 6.5 Hz, 1H, CH_3C*H*-O), 3.80 (dsept, *J* = 8.0, 6.5 Hz, 1H, C*H*-*i*Pr), 3.18 (d, *J* = 8.0 Hz, 1H, N*H*), 3.08 (sept, *J* = 6.2 Hz, 1H, C*H*-*i*Pr), 1.51 (d, *J* = 6.6 Hz, 3H C*H*$_3$CH-O), 1.20 (d, *J* = 6.2 Hz, 3H, C*H*$_3$-*i*Pr), 1.11 (d, *J* = 6.2 Hz, 3H, C*H*$_3$-*i*Pr), 0.95 (d, *J* = 6.5 Hz, 3H C*H*$_3$-*i*Pr), 0.85 (d, *J* = 6.5 Hz, 3H, C*H*$_3$-*i*Pr). ^{13}C{^{1}H} NMR (100 MHz, C_6D_6) δ 150.0 (s, *C*N_2), 144.3 (s, *ipso*-Ph), 128.4 (s, *o/m*-Ph), 127.3 (s, *p*-Ph), 126.6 (s, *m/o*-Ph), 71.6 (s, O*C*HCH_3), 46.6 (s, *C*H-*i*Pr-N), 43.5 (s, *C*H-*i*Pr-NH), 24.9, 24.8 (2 x s, 2 x *C*H_3-*i*Pr-N), 24.1, 24.0 (2 x s, 2 x *C*H_3-*i*Pr-NH), 22.7 (s, OCH*C*H_3). These data are consistent with previous reports.[5]

***N,N'*-diisopropyl-*O*-adamantylisourea (*syn*- and *anti*-1g).** 79.0 μL of DIC (0.50 mmol), 77 mg of 1-adamantanol (0.50 mmol). 60 ºC, 6 h, 60% conversion to isomer mixture of **1g** (isomer ratio = 73:27). ^{1}H NMR (400 MHz, C_6D_6) δ 4.05 (sept, *J* = 6.3 Hz, 1H, NC*H*-*i*Pr, *min*), 3.96 (m, 1H, NHC*H*-*i*Pr, *min*), 3.71 (dsept, *J* = 7.7, 6.4 Hz, 1H, NHC*H*-*i*Pr, *maj*), 3.11 (br, 1H, N*H*, *maj*), 3.11 (sept, *J* = 6.2 Hz, 1H, NC*H*-*i*Pr, *maj*), 2.99 – 2.94 (br, 1H, N*H*, *min*), 2.41 (d, *J* = 3.1 Hz, 6H, C*H*2-Adm, *maj*), 2.06 (br, 3H, C*H*-Adm, *maj*), 1.95 (br, 3H, C*H*-Adm, *min*), 1.92 (m, 6H, C*H*2-Adm, *min*), 1.72 – 1.50 (m, 6H, C*H*2-Adm, *maj*), 1.44 (m, C*H*2-Adm, 6H, *min*), 1.31 (d, *J* = 6.3 Hz, 6H, C*H*3-*i*Pr, *min*), 1.18 (d, *J* = 6.2 Hz, 6H, C*H*3-*i*Pr, *maj*), 1.05 (d, *J* = 6.4 Hz, 6H, C*H*3-*i*Pr, *min*), 0.95 (d, *J* = 6.4 Hz, 6H, C*H*3-*i*Pr, *maj*). ^{13}C{^{1}H} NMR (101 MHz, C_6D_6) δ 149.4 (s, *C*N2, *maj*), 147.4 (s, *C*N2, *min*), 78.0 (s, O*C*-Adm, *maj*), 77.3 (s, O*C*-Adm, *min*), 47.0 (s, N*C*H-*i*Pr, *min*), 46.7 (s, N*C*H-*i*Pr, *min*), 43.7 (s, NH*C*H-*i*Pr, *maj*), 43.6 (s, NH*C*H-*i*Pr, *min*), 43.3 (s, *C*H2-Adm, *min*), 42.4, 37.0 (2 x s, *C*H2-Adm, *maj*), 36.4 (s, *C*H2-Adm, *min*), 31.5 (s, *C*H-Adm, *maj*), 31.4 (s, *C*H-Adm, *min*), 25.6 (s, *C*H3-*i*Pr, *min*), 24.8, 24.0, (2 x s, *C*H3-*i*Pr, *maj*), 22.9 (s, *C*H3-*i*Pr, *min*).

***N,N'*-diisopropyl-*O*-phenylisourea (1h).** 79.0 μL of DIC (0.50 mmol), 47 mg of phenol (0.50 mmol), 60 ºC, 1 h, 58% conversion. ^{1}H NMR (500 MHz, C_6D_6) δ 7.04 (m, 2H, *o/m*-Ph), 6.95 (m, 2H, *m/o*-Ph), 6.81 (tt, *J* = 7.2, 1.2 Hz, 1H, *p*-Ph), 5.74 (br, 1H, N*H*), 3.82 (sept, *J* = 6.5 Hz, 2H, C*H*-*i*Pr), 1.02 (*J* = 6.5 Hz, 12H, C*H*3-*i*Pr). These data are consistent with previous reports.[2c, 3, 6]

***N,N'*-diisopropyl-*O*-(2,6-dimethyl)phenylisourea (*syn*- and *anti*-1i).** 79.0 μL of DIC (0.50 mmol), 62 mg of 2,6-dimethylphenol (0.50 mmol), 60 ºC, 1 h, 68% conversion to isomer mixture of **1i** (isomer ratio = 64:36). <u>Partial spectroscopic data for **1i**:</u> ^{1}H NMR (400 MHz, C_6D_6) δ 4.21 (sept, *J* = 6.3 Hz, 2H, C*H*-*i*Pr, *maj*), 3.93 (m, 1H, C*H*-*i*Pr, *min*), 3.70 (s, br, 1H, N*H*, *maj*), 3.28 (d, *J* = 7.8 Hz, 1H, N*H*, *min*), 3.03 (sept, *J* = 6.2 Hz, 1H, C*H*-*i*Pr, *min*), 2.27 (s, 6H, *Me*2-C6H3, *min*), 2.14 (s, 6H, *Me*2-C6H3, *maj*), 1.13 (d, *J* = 6.6 Hz, 12H, C*H*3-*i*Pr, *maj*), 1.01 (d, *J* = 6.4 Hz, 12H, C*H*3-*i*Pr, *min*). Signals owing to aromatic protons of **1i** overlapped with those of 2,6-dimethylphenol and could not be unambiguously assigned. The NH signal of **1i** (major isomer, *maj*) and the OH of the phenol are isochronic, as well as the Me signals of the mesityl groups of the phenol and **1i** (*maj*) probably due to exchange equilibrium. ^{13}C{^{1}H} NMR (101 MHz, C_6D_6) δ 46.9 (s, *C*H-*i*Pr, *min*), 44.7 (s, *C*H-*i*Pr, *maj*) 44.1 (s, *C*H-*i*Pr, *min*), 24.4, 24.3 (2 x s, *C*H3-*i*Pr, *min*) 24.1 (s, *C*H3-*i*Pr, *maj*), 17.0 (s, *Me*2-C6H3, *min*), 16.2 (s, *Me*2-C6H3, *maj*). Signals

owing to aromatic carbons and central CN_2 carbons in **1i** could not be unambiguously assigned.

N,N'*-diisopropyl-*O*-(2,6-di-*tert*-butyl)phenylisourea (1j).** 79.0 µL of DIC (0.50 mmol), 104 mg of 2,6-di-*tert*-butylphenol (0.50 mmol), 60 ºC, 6 h, 14% conversion. <u>*Partial spectroscopic data for **1j:</u> ^{1}H NMR (500 MHz, C_6D_6) δ 7.18 (d, J = 7.9 Hz, 2H, *m*-Ar,), 6.95 (t, J = 7.9 Hz, 1H, *p*-Ar), 4.51 (sept, J = 6.3 Hz, 1H, C*H*-*i*Pr), 3.88 (sept, J = 6.5 Hz, 1H, C*H*-*i*Pr), 1.41 (s, 18H, *t*Bu).

N,N'-diisopropyl-*O*-(2-methylthio)ethylisourea (1k). 79.0 µL of DIC (0.50 mmol), 43.5 µL of 2-(methylthio)ethanol (0.50 mmol), 60 ºC, 48 h, 95% conversion. <u>*Preparative scale*</u>: 330 µL of DIC (2.11 mmol), 180 µL of (2-methylthio)ethanol (1.98 mmol) in toluene (2 mL), 60 ºC, 72 h (colourless oil, yield: 405 mg, 92%). ^{1}H NMR (400 MHz, C_6D_6) δ 4.32 (t, J = 6.8 Hz, 2H, O-CH_2), 3.74 (dsept, J = 7.9, 6.5 Hz, 1H, C*H*-*i*Pr), 3.20 (d, J = 7.9 Hz, 1H, N*H*), 3.09 (sept, J = 6.3 Hz, 1H, C*H*-*i*Pr), 2.59 (t, J = 6.8 Hz, 2H, S-CH_2), 1.81 (s, 3H, S*Me*), 1.16 (d, J = 6.3 Hz, 6H, CH_3-*i*Pr), 0.92 (d, J = 6.5 Hz, 6H, CH_3-*i*Pr). ^{13}C{^{1}H} NMR (101 MHz, C_6D_6) δ 150.7 (s, *C*N_2), 63.8 (s, O*C*H_2), 46.5, 43.5 (2 x s, 2 x *C*H-*i*Pr), 33.3 (s, S*C*H_2), 24.8, 24.0 (2 x s, 2 x *C*H_3-*i*Pr), 15.51 (s, S*Me*). Anal calc. for $C_{10}H_{22}N_2OS$: C, 55.00; H, 10.16; N, 12.83. Found: C, 55.12; H, 9.89; N, 12.66. ESI-MS: Exact Mass: 218.15; $[M^*+H]^+$ 219.15 (Found: 219.15)

Pyridine-2,6-bis(*N,N'*-diisopropyl-*O*-methyleneisourea) (1l). 79.0 µL of DIC (0.50 mmol), 36 mg of 2,6-pyridinedimethanol (0.25 mmol), 60 ºC, 4 h, >99% conversion. <u>*Preparative scale*</u>: 158 µL of DIC (1.00 mmol), 71 mg of 2,6-pyridinedimethanol (0.50 mmol) in toluene (2 mL), 60 ºC, 4 h (white solid, yield: 142 mg, 74%). ^{1}H NMR (500 MHz, C_6D_6) δ 7.22 (s, 3H, C*H*-py), 5.55 (s, 4H, O-*C*H_2), 3.79 (m, 2H, C*H*-*i*Pr), 3.24 (d, J = 8.1 Hz, 2H, N*H*), 3.11 (sept, J = 6.1 Hz, 2H, C*H*-*i*Pr), 1.18 (d, J = 6.1 Hz, 12H, CH_3-*i*Pr), 0.91 (d, J = 6.6 Hz, 12H, CH_3-*i*Pr).^{13}C{^{1}H} NMR (126 MHz, C_6D_6) δ 158.3 (s, $C^{2,6}$-py), 150.6 (s, *C*N_2), 136.4 (s, C^4-py), 119.7 (s, $C^{3,5}$-py), 67.8 (s, O-*C*H_2), 46.6, 43.6, (2 x s, 2 x *C*H-*i*Pr), 24.7, 24.0 (2 x s, 2 x *C*H_3-*i*Pr). Anal calc. for $C_{21}H_{37}N_5O_2$: C, 64.42; H, 9.52; N, 17.89. Found: C, 64.29; H, 9.31; N, 17.98. ESI-MS: Exact Mass: 391.29; $[M^*+H]^+$ 392.30 (Found: 392.30).

***N,N'*-dicyclohexyl-*O*-methylisourea (2a).** 104 mg of *N,N'*-dicyclohexylcarbodiimide (DCC, 0.50 mmol), 21.0 µL of MeOH (0.50 mmol), 60 ºC, 1 h, >99% conversion. ^{1}H NMR (500 MHz, C_6D_6) δ 3.67 (s, OCH_3, 3H), 3.47 – 3.34 (m, 2H, C*H*-Cy+NH), 2.95 (tt, J = 9.4, 3.8 Hz, 1H, C*H*-Cy), 2.29 – 0.68 (m, 20H, CH_2-Cy). ^{13}C{^{1}H} NMR (126 MHz, C_6D_6) δ 151.4 (s, *C*N_2), 54.9 (s, C^1-Cy), 52.4 (s, O-*C*H_3), 50.6 (s, C^1-Cy), 35.2, 34.8 (2 x

s, 2 x C^2-Cy), 26.7, 26.0 (2 x s, 2 x C^4-Cy), 25.4 (s, 2 x C^3-Cy). These data are consistent with previous reports.[7]

N,N'*-di-*tert*-butyl-*O*-methylisourea (3a).** 97.4 µL of *N,N'*-di-*tert*-butylcarbodiimide (0.50 mmol), 21.0 µL of MeOH (0.50 mmol), 60 ºC, 30 h, 27% conversion. *Partial NMR data for **3a: ^{1}H NMR (500 MHz, C_6D_6) δ 3.57 (s, OCH_3, 3H), 1.28, 1.18 (2 x s, 2 x 9H, *t*Bu).

***N,N'*-di-*p*-tolyl-*O*-methylisourea (4a).** 112 mg of *N,N'*-di-*p*-tolylcarbodiimide (DTC, 0.50 mmol), 21.0 µL of MeOH (0.50 mmol), 25 ºC, 1.5 h, >99% conversion. ^{1}H NMR (400 MHz, C_6D_6) δ 7.00 (m, 4H, *H*-Ar), 6.79 (m, 4H, *H*-Ar), 5.89 (s, 1H, N*H*), 3.73 (s, 3H, O-CH_3), 2.16, 2.06 (2 x s, 2 x 3H, *p*-CH_3). ^{13}C{^{1}H} NMR (101 MHz, C_6D_6) δ 150.9 (s, *C*N_2), 146.3, 136.7, 132.5, 132.1 (4 x s, 4 x *C*-Ar), 130.7, 129.6, 123.0, 121.2 (4 x s, *C*H-Ar), 53.7 (s, O-*C*H_3), 20.8, 20.7 (2 x s, *p*-*C*H_3). These data are consistent with previous reports.[2a, c, 8]

***N,N'*-di-*p*-tolyl-*O*-ethylisourea (4b).** 112 mg of DTC (0.50 mmol), 29.0 µL of EtOH (0.50 mmol), 25 ºC, 2 h, >99% conversion. ^{1}H NMR (400 MHz, C_6D_6) δ 7.02 (m, 4H, *H*-Ar), 6.81 (m, 4H, *H*-Ar), 5.88 (s, 1H, N*H*), 4.36 (q, *J* = 7.1 Hz, 2H, CH_2-Et), 2.17, 2.07 (2 x s, 2 x 3H, *p*-CH_3), 1.14 (t, *J* = 7.1 Hz, 3H, CH_3-Et). ^{13}C{^{1}H} NMR (101 MHz, C_6D_6) δ 150.1 (s, *C*N_2), 146.5, 136.9, 132.3, 132.0 (4 x s, 4 x *C*-Ar), 130.7, 129.6, 123.0, 120.9 2 (4 x s, *C*H-Ar), 62.7 (s, *C*H_2-Et), 20.8, 20.7 (2 x s, *p*-*C*H_3), 14.4 (s, *C*H_3-Et). These data are consistent with previous reports.[2a, c, 8]

***N,N'*-di-*p*-tolyl-*O*-isopropylisourea (4c).** 112 mg of DTC (0.50 mmol), 38.5 µL of *i*PrOH (0.50 mmol), 25 ºC, 2 h, >99% conversion. ^{1}H NMR (400 MHz, C_6D_6) δ 7.02 (m, 4H, *H*-Ar), 6.81 (m, 4H, *H*-Ar), 5.87 (s, 1H, N*H*), 5.46 (sept, *J* = 6.2 Hz, 1H, C*H*-*i*Pr), 2.17, 2.06 (2 x s, 2 x 3H, *p*-CH_3), 1.26 (d, *J* = 6.3 Hz, 6H, CH_3-*i*Pr). ^{13}C{^{1}H} NMR (101 MHz, C_6D_6) δ 149.6 (s, *C*N_2), 146.6, 137.0, 132.1, 131.9 (4 x s, 4 x *C*-Ar), 130.7, 129.6, 122.9, 120.8 (4 x s, *C*H-Ar), 69.6 (s, *C*H-*i*Pr), 22.0 (s, *C*H_3-*i*Pr), 20.9, 20.7 (2 x s, *p*-*C*H_3). These data are consistent with previous reports.[2a, c, 8]

***N,N'*-di-*p*-tolyl-*O*-*tert*-butylisourea (4d).** 112 mg of DTC (0.50 mmol), 48.3 µL of *t*BuOH (0.50 mmol), 60 ºC, 1.5 h, >99% conversion. *Preparative scale*: 112 mg of DTC (0.50 mmol), 48.3 µL of *t*BuOH (0.50 mmol), in toluene (2 mL), 60 ºC, 2 h (white solid, yield: 136 mg, 92%). ^{1}H NMR (500 MHz, C_6D_6) δ 6.99 (m, 4H, *H*-Ar), 6.80 (m, 4H, *H*-Ar), 5.78 (s, 1H, N*H*), 2.17, 2.07 (2 x s, 2 x 3H, *p*-CH_3), 1.60 (s, 9H, *t*Bu). ^{13}C{^{1}H} NMR (126 MHz, C_6D_6) δ 148.6 (s, *C*N_2), 146.5, 137.4, 131.9, 131.7 (4 x s, 4 x *C*-Ar), 130.6,

129.5, 122.8, 120.7 (4 x s, *C*H-Ar), 81.2 (s, *C*-*t*Bu), 28.5 (s, *C*H_3-*t*Bu), 20.9, 20.7 (2 x s, *p*-*C*H_3). These data are consistent with previous reports.[2a, c, 8]

***N,N'*-di-*p*-tolyl-*O*-benzylisourea (4e).** 112 mg of DTC (0.50 mmol), 51.8 μL of benzyl alcohol (0.50 mmol), 25 ºC, 0.3 h, >99% conversion. ^{1}H NMR (400 MHz, C_6D_6) δ 7.31 (m, 2H, *o*/*m*-Ph), 7.12 (m, 2H, *m*/*o*-Ph), 7.07 (m, 1H, *p*-Ph), 7.02 (m, 4H, *H*-*p*-tol), 6.77 (br, 4H, *H*-*p*-tol), 5.96 (s, 1H, N*H*), 5.43 (s, 2H, O-C*H*$_2$), 2.16, 2.01 (2 x s, 2 x 3H, *p*-C*H*$_3$). ^{13}C{^{1}H} NMR (101 MHz, C_6D_6) δ 150.2 (s, *C*N_2), 146.2 (s, *C*-*p*-tol), 137.4 (s, *ipso*-Ph), 136.6, 132.6, 132.2 (3 x s, 3 x *C*-*p*-tol), 130.7, 129.6 (2 x s, 2 x *C*H-*p*-tol), 128.7, 128.4 (2 x s, *o*/*m*-Ph), 128.1 (s, *p*-Ph), 123.0, 121.2 (2 x s, 2 x *C*H-*p*-tol), 68.7 (s, O-*C*H_2) 20.9, 20.7 (2 x s, *p*-*C*H_3). These data are consistent with previous reports.[2b, 5, 8b, 9]

***N,N'*-di-*p*-tolyl-*O*-(1-phenyl)ethylisourea (4f).** 112 mg of DTC (0.50 mmol), 61.6 μL of 1-phenylethanol (0.50 mmol), 25 ºC, 0.2 h, >99% conversion. *Preparative scale*: 112 mg of DTC (0.50 mmol), 61.6 μL of 1-phenylethanol (0.50 mmol), in toluene (2 mL), 25 ºC, 0.2 h (colorless oil, yield: 160 mg, 93%). ^{1}H NMR (400 MHz, C_6D_6) δ 7.37 (m, 2H, *o*/*m*-Ph), 7.15 (m, 2H, *m*/*o*-Ph), 7.07 (m, 1H, *p*-Ph), 7.02 – 6.78 (m, 8H, *p*-tol), 6.47 (q, *J* = 6.5 Hz, OC*H*-CH_3, 1H), 5.87 (s, 1H, N*H*), 2.14, 2.06 (2 x s, 2 x 3H, *p*-C*H*$_3$), 1.57 (d, *J* = 6.5 Hz, 3H, OCH-C*H*$_3$). ^{13}C{^{1}H} NMR (126 MHz, C_6D_6) δ 149.3 (s, *C*N_2), 146.3 (s, *C*-*p*-tol), 143.1 (s, *ipso*-Ph), 136.7, 132.5, 132.0 (3 x s, 3 x *C*-*p*-tol), 130.6, 129.6 (2 x s, 2 x *C*H-*p*-tol), 128.6 (s, *o*/*m*-Ph), 127.7 (s, *p*-Ph), 126.6 (s, *m*/*o*-Ph), 122.9, 121.2 (2 x s, 2 x *C*H-*p*-tol), 74.5 (s, O-*C*HCH_3), 22.7 (s, O-CH*C*H_3), 20.8, 20.7 (2 x s, *p*-*C*H_3). Anal calc. for $C_{23}H_{24}N_2O$: C, 80.20; H, 7.02; N, 8.13. Found: C, 80.01; H, 6.96; N, 8.22. ESI-MS: Exact Mass: 344.19; $[M^*+H]^+$ 345.19 (Found: 345.20).

***N,N'*-di-*p*-tolyl-*O*-adamantylisourea (4g).** 112 mg of DTC (0.50 mmol), 77 mg of 1-adamantanol (0.50 mmol). 60 ºC, 7 h, >99% conversion. ^{1}H NMR (500 MHz, C_6D_6) δ 7.12 – 6.71 (m, 8H, *H*-Ar), 5.79 (s, 1H, N*H*), 2.44 (s, 6H, C*H*$_2$-Adm), 2.17, 2.10 (2 x s, 2 x 3H, *p*-C*H*$_3$), 2.03 (s, 3H, C*H*-Adm), 1.52 (m, 6H, C*H*$_2$-Adm). ^{13}C{^{1}H} NMR (101 MHz, C_6D_6) δ 148.1 (s, *C*N_2), 146.5 137.5 (2 x s, 2 x *C*-Ar), 131.7 (s, 2 x *C*-Ar), 130.6, 129.5, 122.8, 120.7 (4 x s, 4 x *C*H-Ar), 81.3 (s, *C*-Adm), 42.1, 36.7 (2 x s, *C*H_2-Adm), 31.5 (s, *C*H-Adm), 20.9 (s, *p*-*C*H_3). These data are consistent with previous reports.[2b, 10]

***N,N'*-di-*p*-tolyl-*O*-phenylisourea (4h).** 112 mg of DTC (0.50 mmol), 47 mg of phenol (0.50 mmol), 60 ºC, 1 h, >99% conversion. ^{1}H NMR (500 MHz, C_6D_6) δ 7.30 – 6.70 (m, 13H, *H*-Ar), 6.30 – 5.50 (br, 1H, N*H*), 2.07 (br, 6H, *p*-C*H*$_3$). ^{13}C{^{1}H} NMR (126 MHz, C_6D_6) δ 157.5 (s, *C*-Ar), 153.6 (br, *C*N_2), 132.8 (br, *C*-Ar), 129.8 (br, *C*H-Ar), 129.6 (s,

*C*H-Ar), 124.9 (br, *C*H-Ar), 121.9 (br, *C*H-Ar), 20.8 (s, *p*-*C*H_3). These data are consistent with previous reports.[2a, c, 8]

***N,N'*-di-*p*-tolyl-*O*-(2,6-dimethyl)phenylisourea (4i).** 112 mg of DTC (0.50 mmol), 62 mg of 2,6-dimethylphenol (0.50 mmol), 25 ºC, 0.3 h, >99% conversion. ^{1}H NMR (400 MHz, C_6D_6) δ 6.91 (br, 11H, Ar), 5.94 (br, 1H, N*H*), 2.30 (s, 6H, *o*-C*H₃*), 2.11 (br, 6H, *p*-C*H₃*). ^{13}C{^{1}H} NMR (101 MHz, C_6D_6) δ 150.2 (s, *ipso*-Mes), 148.4 (br, *C*N_2), 145.9, 136.5, 133.0, 132.2 (4 x br, 4 x *C*-*p*-tol), 131.1 (s, *o*-Mes), 130.4, 129.8 (2 x br, 2 x *C*H-*p*-tol), 128.9 (s, *m*-Mes), 125.6 (s, *p*-Mes), 122.7, 121.5 (2 x br, 2 x *C*H-*p*-tol), 20.8 (s, *p*-*C*H_3), 16.9 (s, *o*-*C*H_3). These data are consistent with previous reports.[8, 10]

***N,N'*-di-*p*-tolyl-*O*-(2,6-di-*tert*-butylphenyl)isourea (4j).** 112 mg of DTC (0.50 mmol), 104 mg of 2,6-di-*tert*-butylphenol (0.50 mmol), 25 ºC, 0.3 h (88% conversion, isomer ratio = 74:26). ^{1}H NMR (500 MHz, C_6D_6) δ 7.82 – 6.43 (m, Ar, *both*), 6.06 (s, 1H, N*H*, *maj*), 5.49 (s, 1H, N*H*, *min*), 2.18 (s, 3H, *p*-CH_3, *min*), 2.14, 2.05 (2 x s, 2 x 3H, *p*-CH_3, *maj*), 2.04 (s, 3H, *p*-CH_3, *min*), 1.51 (s, 18H, *t*Bu, *maj*), 1.37 (s, 18H, *t*Bu, *min*).

***N,N'*-di-*p*-tolyl-*O*-(2-methylthio)ethylisourea (4k).** 112 mg of DTC (0.50 mmol), 43.5 µL of 2-(methylthio)ethanol (0.50 mmol), 25 ºC, 3 h (96% conversion). *Preparative scale*: 224 mg of DTC (1.00 mmol), 93.0 µL of 2-(methylthio)ethanol (1.06 mmol), toluene, 25 ºC, 6 h (colorless oil, yield: 300 mg, 95%). ^{1}H NMR (500 MHz, C_6D_6) δ 7.02 (m, 4H, *H*-Ar), 6.84 (m, 4H, *H*-Ar), 5.88 (s, 1H, N*H*), 4.48 (t, *J* = 6.7 Hz, 2H, O-CH_2), 2.56 (t, *J* = 6.7 Hz, 2H, S-CH_2), 2.17, 2.05 (2 x s, 2 x 3H, *p*-CH_3), 1.74 (s, 3H, S*Me*). ^{13}C{^{1}H} NMR (126 MHz, C_6D_6) δ 150.0 (s, *C*N_2), 146.2, 136.6, 132.6, 132.2 (4 x s, 4 x *C*-Ar), 130.7, 129.6, 122.9, 121.3 (4 x s, 4 x *C*H-Ar), 65.4 (s, O*C*H_2), 32.9 (s, S*C*H_2), 20.9, 20.7 (2 x s, *p*-*C*H_3), 15.4 (s, S*Me*). Anal calc. for $C_{18}H_{22}N_2OS$: C, 68.75; H, 7.05; N, 8.91. Found: C, 68.88; H, 6.99; N, 8.79. ESI-MS: Exact Mass: 314.15; $[M^*+H]^+$ 315.15 (Found: 315.15).

***N,N'*-di-*p*-tolyl-*O*-methyleneisourea dimer (4l).** 112 mg of DTC (0.50 mmol), 14.1 µL of ethylene glycol (0.25 mmol), 25 ºC, 7 h, >99% conversion. *Preparative scale*: 224 mg of DTC (1.00 mmol), 28.2 µL of ethylene glycol (0.50 mmol), 23 h, toluene (white solid, yield: 230 mg, 91%). ^{1}H NMR (500 MHz, C_6D_6) δ 7.00 (m, 8H, *H*-Ar), 6.80 (s, 8H, *H*-Ar), 5.90 (s, 2H, N*H*), 4.66 (s, 4H, O-CH_2), 2.16, 2.06 (2 x s, 2 x 6H, *p*-CH_3). ^{13}C{^{1}H} NMR (126 MHz, C_6D_6) δ 149.9 (s, *C*N_2), 146.1, 136.6, 132.3, 132.1 (4 x s, 4 x *C*-Ar), 130.7, 129.7, 123.0, 120.9 (4 x s, 4 x *C*H-Ar), 65.0 (s, O*C*H_2), 20.9, 20.7 (2 x s, *p*-*C*H_3). Anal calc. for $C_{32}H_{34}N_4O_2$: C, 75.86; H, 6.76; N, 11.06. Found: C, 75.57; H, 6.81; N, 11.18. ESI-MS: Exact Mass: 506.27; $[M^*+H]^+$ 507.27 (Found: 507.27).

***N,N'*-bis-(2,6-diisopropylphenyl)-*O*-methylisourea (5a).** 185 mg of *N,N'*-bis-(2,6-diisopropylphenyl)carbodiimide (0.50 mmol), 21.0 µL of methanol (0.50 mmol), 80 ºC, 120 h (97% conversion). <u>*Preparative scale*</u>: 185 mg of *N,N'*-bis-(2,6-diisopropylphenyl)carbodiimide (0.50 mmol), 25.0 µL of methanol (0.55 mmol), in C_6D_6 (0.5 mL) at 80 ºC, 7 days (170 mg, white solid, 89%). Crystals of **5a** suitable for an X-ray diffraction analysis were grown by slow evaporation from a saturated solution in hexane at 25 ºC. ^{1}H NMR (500 MHz, C_6D_6) δ 7.26 (d, *J* = 7.6 Hz, 2H, *m*-Ar), 7.16 (t, *J* = 7.6 Hz, 1H, *p*-Ar), 7.11 (t, *J* = 7.7 Hz, 1H, *p*-Ar), 7.01 (d, *J* = 7.7 Hz, 2H, *m*-Ar), 4.78 (s, 1H, N*H*), 3.63 (s, 3H, O*Me*), 3.42 (sept, *J* = 6.9 Hz, 2H, C*H-i*Pr), 3.18 (sept, *J* = 6.9 Hz, 2H, C*H-i*Pr), 1.40 (d, *J* = 6.9 Hz, 6H, CH_3-*i*Pr), 1.33 (d, *J* = 6.8 Hz, 6H, CH_3-*i*Pr), 1.20, 1.10 (2 x br, 2 x 6H, CH_3-*i*Pr). ^{13}C{^{1}H} NMR (126 MHz, C_6D_6) δ 149.8 (s, *C*N_2), 146.9 (s, *o*-Ar), 143.3 (s, *ipso*-Ar), 140.4 (s, *o*-Ar), 132.8 (s, *ipso*-Ar), 128.1, 123.9 (2 x s 2 x *p*-Ar), 123.7, 123.4 (2 x s 2 x *m*-Ar), 53.4 (s, O*Me*), 28.71, 28.66 (2 x s, 2 x *C*H-*i*Pr), 24.6 (s, *C*H_3-*i*Pr), 23.9 (br, *C*H_3-*i*Pr), 22.7 (s, *C*H_3-*i*Pr). These data (^{1}H NMR) are consistent with previous reports.[11] ESI-MS: Exact Mass: 394.30; $[M^*+H]^+$ 395.30 (Found: 395.31).

Synthesis of [ZnEt(O*t*Bu)]$_4$ (6). The synthesis of this compound follows a modified literature procedure.[12] In the glovebox, 1 ml of 1.0 M solution of $ZnEt_2$ (1.0 mmol) was added to a cold solution (-20 ºC) of *t*BuOH (95.5 µL, 1.0 mmol) in hexane (4 mL) and the solution was stirred for 30 min and allowed to reach room temperature. After that time, the solvent was removed under vacuum to yield of [ZnEt(O*t*Bu)]$_4$ (**6**) as a white solid (165 mg, 98%). ^{1}H NMR (500 MHz, C_6D_6) δ 1.56 (t, *J* = 8.1 Hz, 3H, CH_3-Et), 1.31 (s, 9H, CH_3-*t*Bu), 0.62 (d, *J* = 8.1 Hz, 2H, CH_3-Et). ^{13}C{^{1}H} NMR (126 MHz, C_6D_6) δ 73.2 (s, *C*-*t*Bu), 32.3 *C*H_3-tBu), 13.4 (s, *C*H_3-Et), 5.6 (s, *C*H_2-Et).

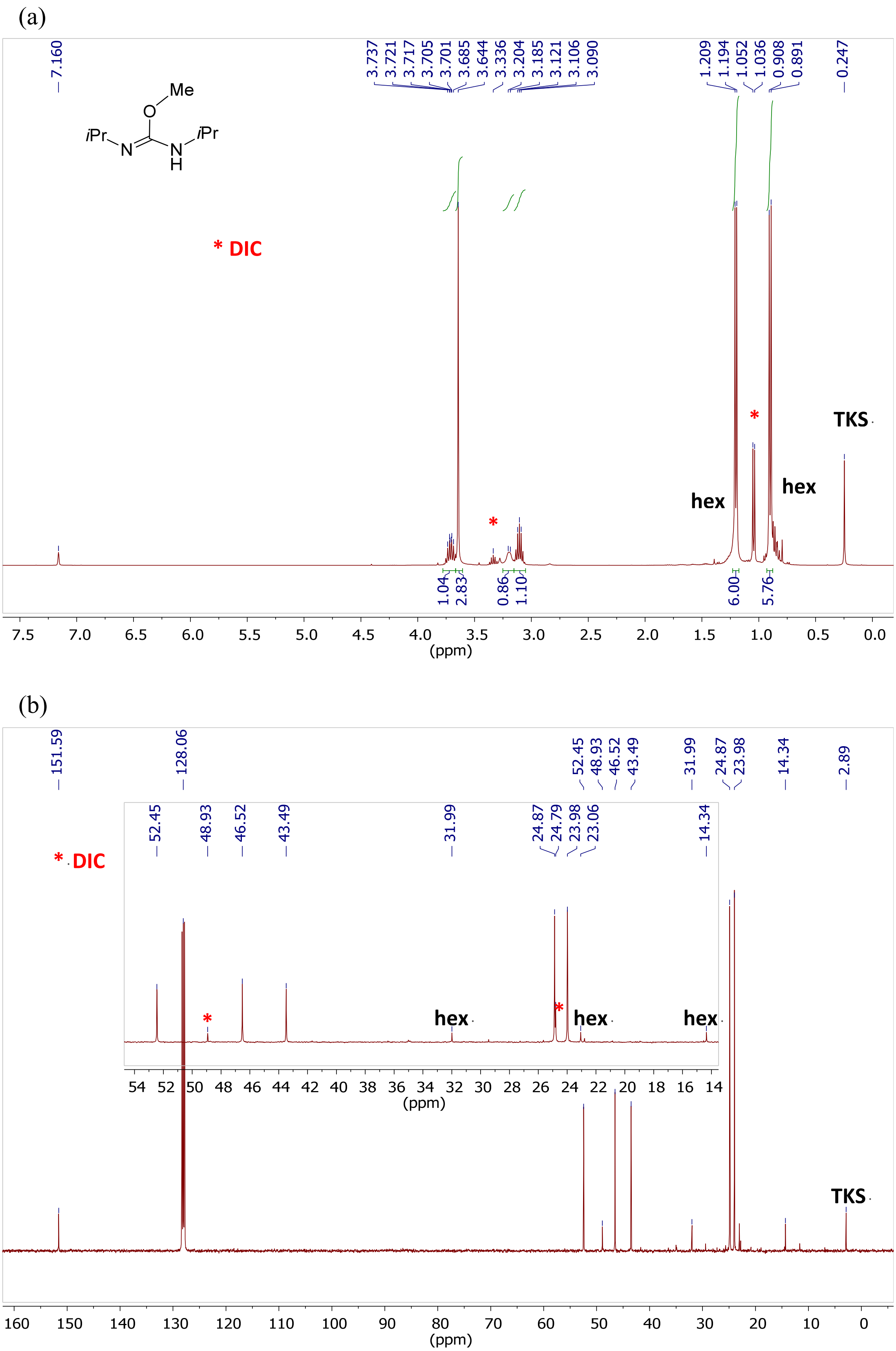

Figure S1. ^{1}H (a) and ^{13}C{^{1}H} (b) NMR in C_6D_6 of compound **1a** (reaction crude; hex = hexane).

(a)

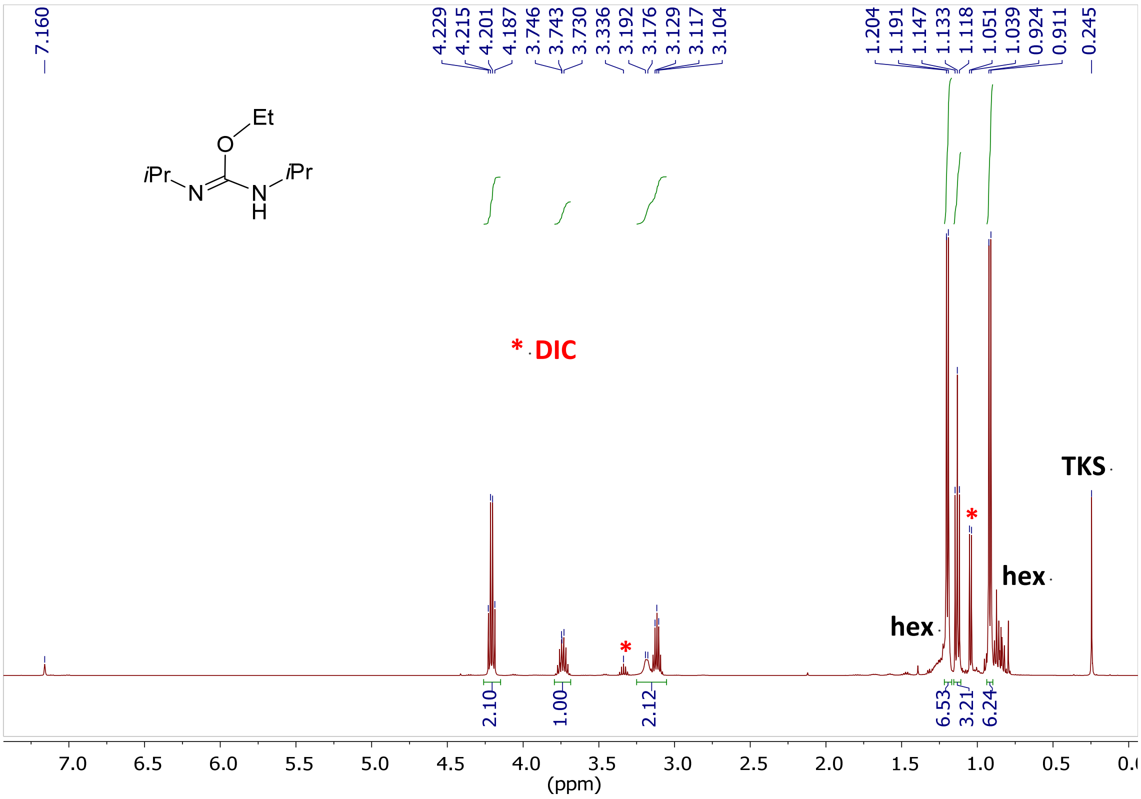

(b)

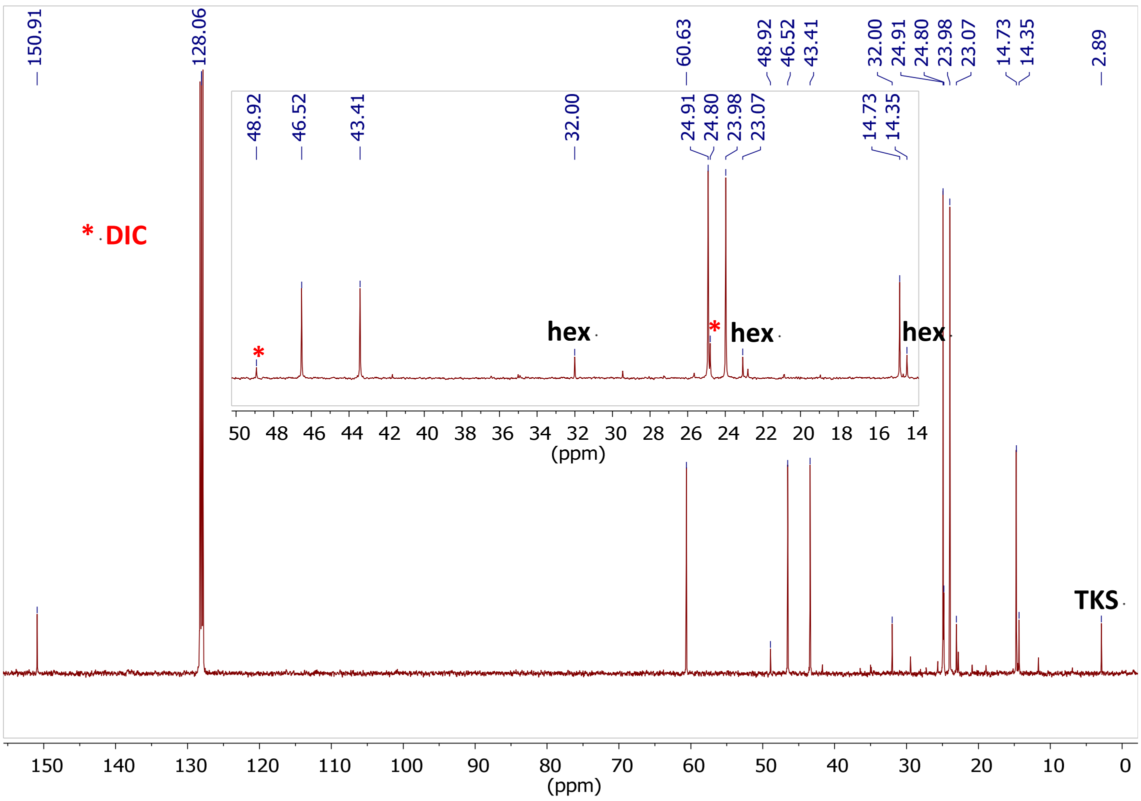

Figure S2. ^{1}H (a) and ^{13}C{^{1}H} (b) NMR in C_6D_6 of compound **1b** (reaction crude).

(a)

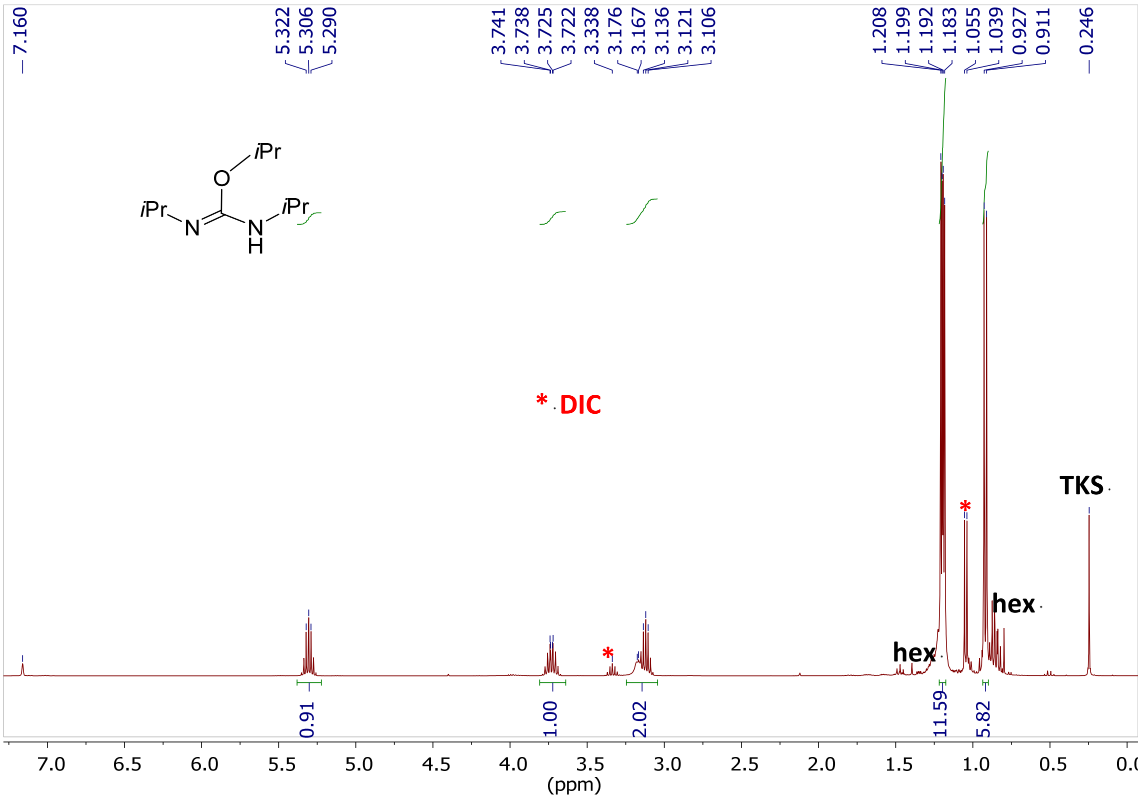

(b)

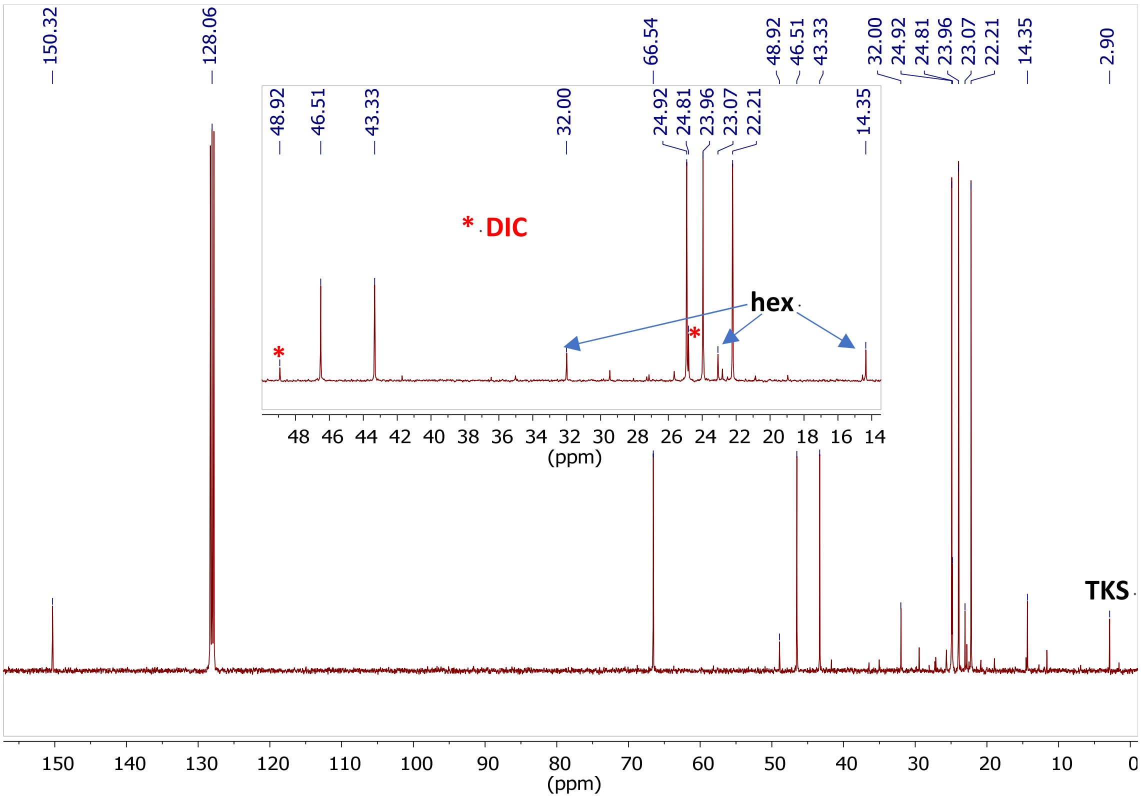

Figure S3. ^{1}H (a) and $^{13}C\{^{1}H\}$ (b) NMR in C_6D_6 of compound **1c** (reaction crude).

(a)

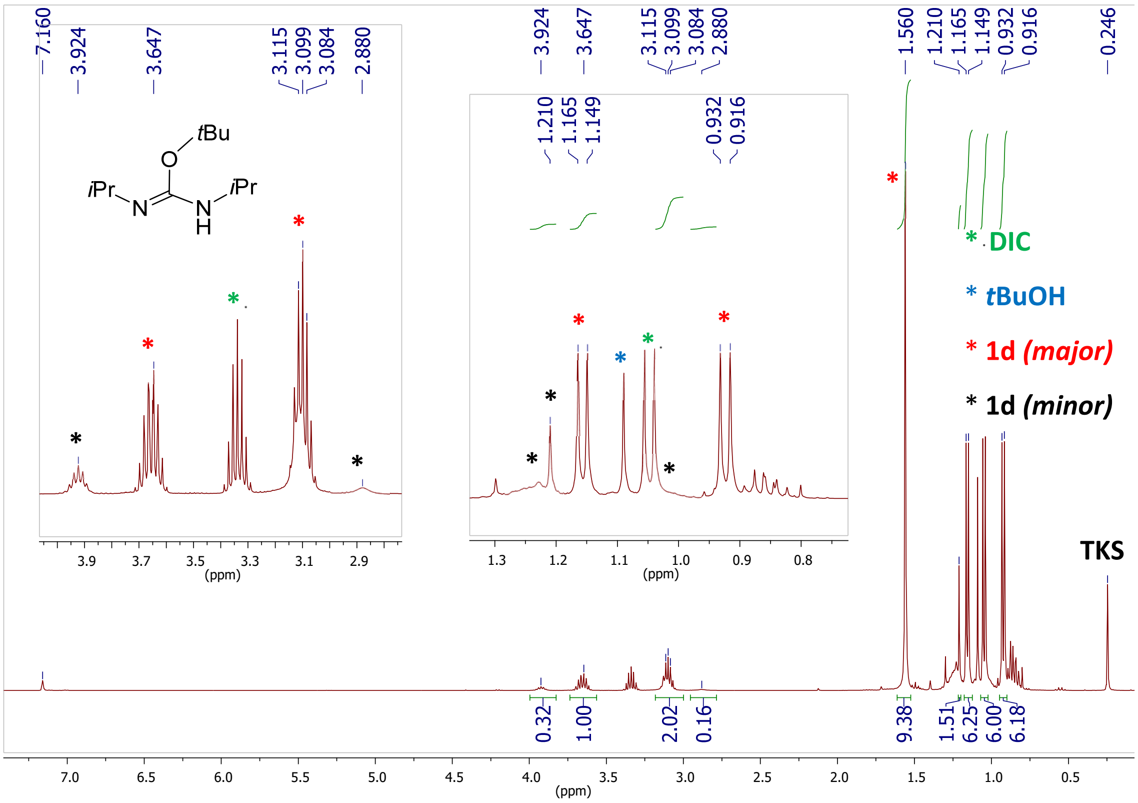

tBu
O
iPr
N
N
H
iPr
* DIC
* tBuOH
* 1d (major)
* 1d (minor)
TKS
(ppm)

(b)

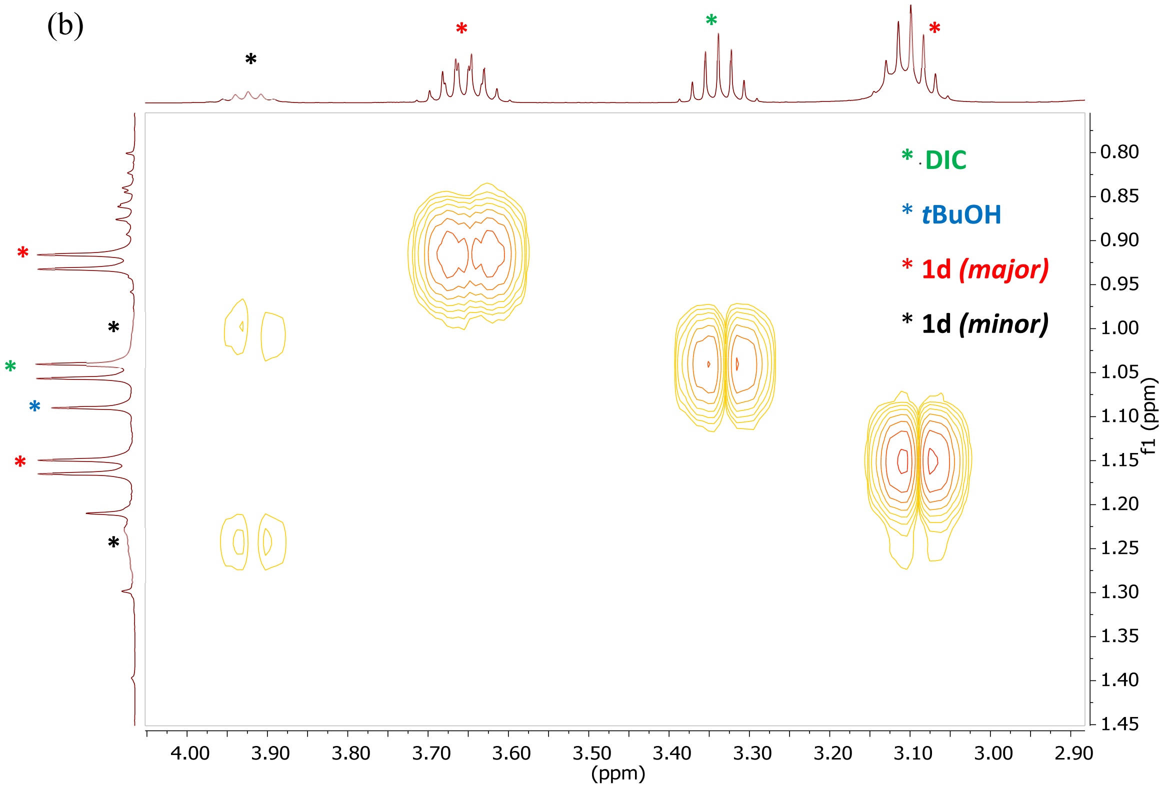

* DIC
* tBuOH
* 1d (major)
* 1d (minor)
f1 (ppm)
(ppm)

(c)

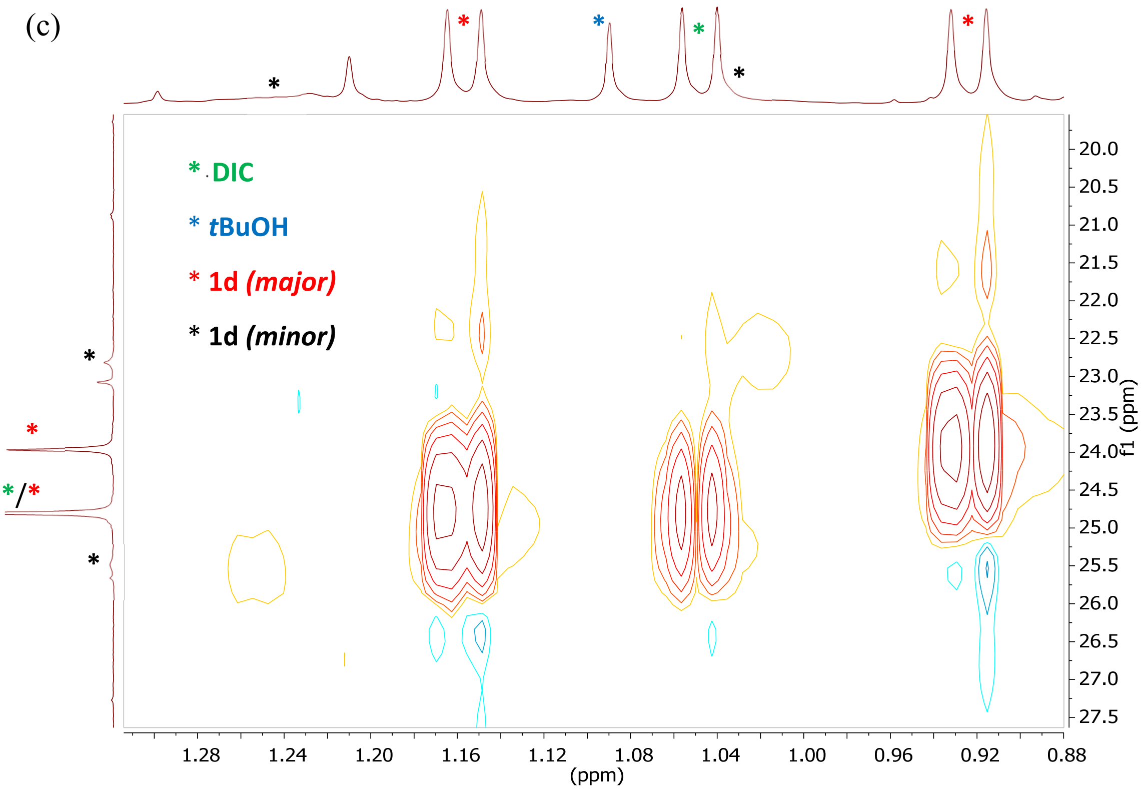

(d)

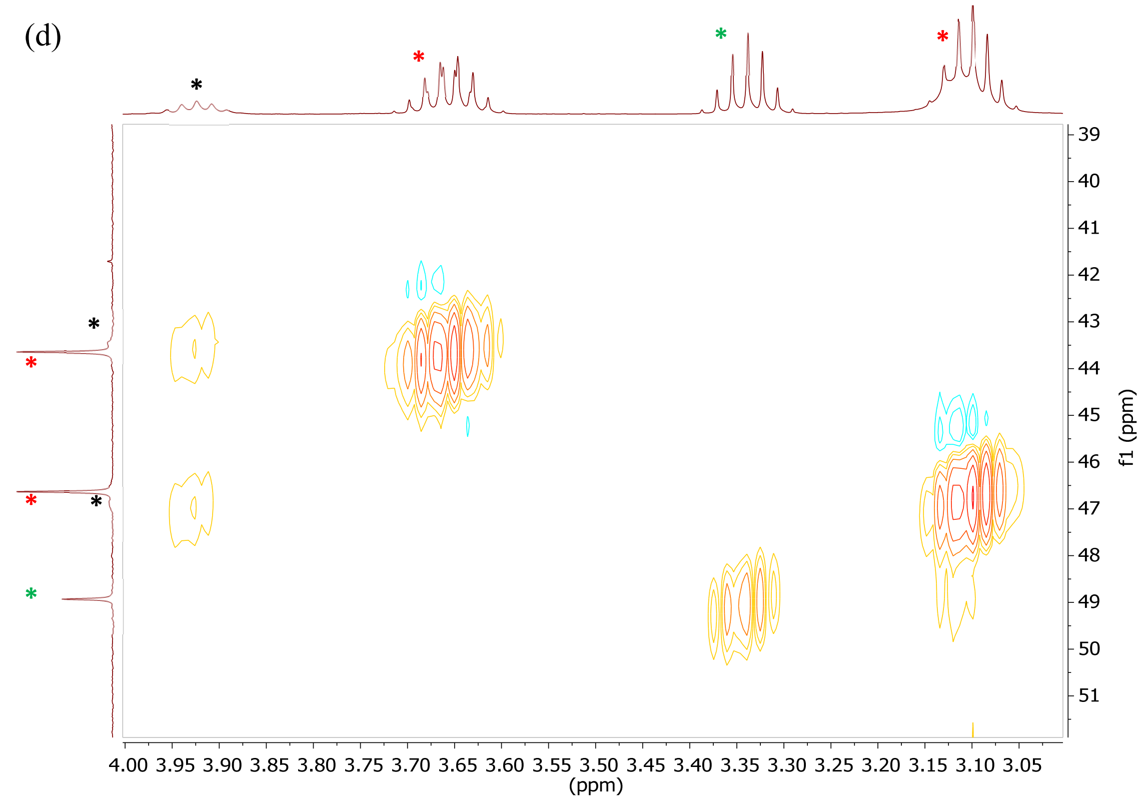

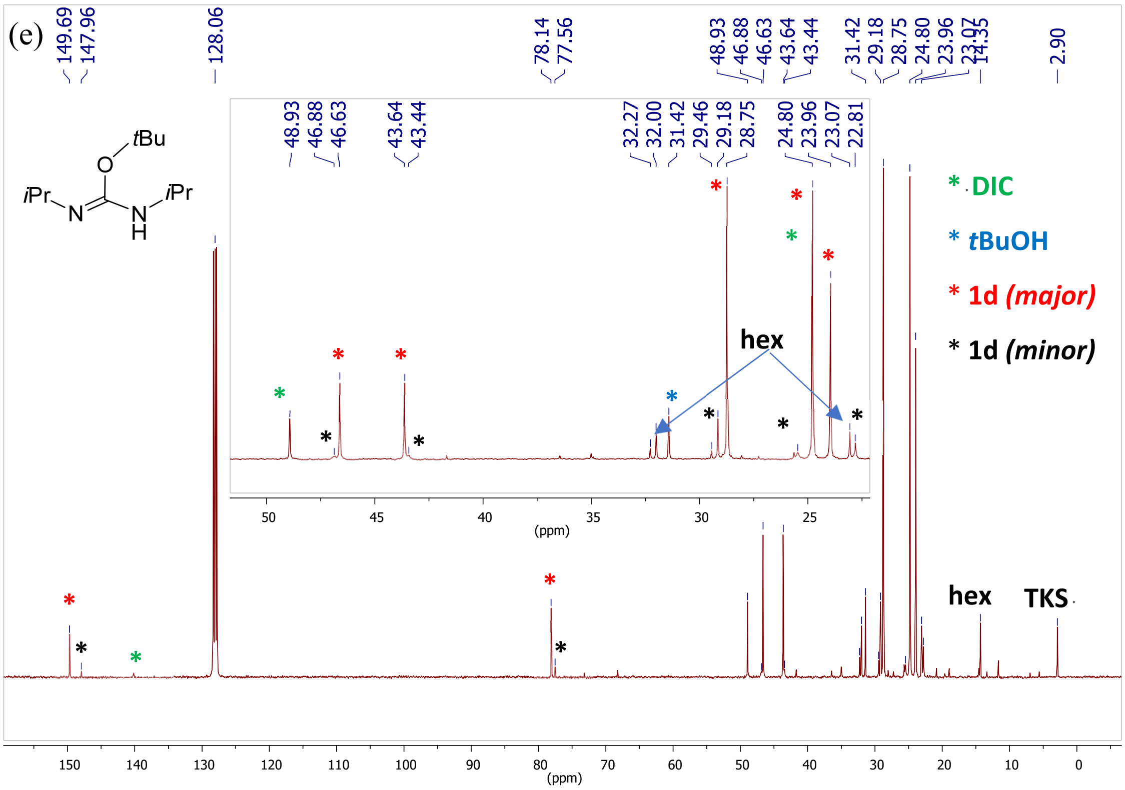

Figure S4. ^{1}H (a), ^{1}H-^{1}H COSY (b), ^{1}H-^{13}C HSQC (c-d) and ^{13}C{^{1}H} (e) NMR in C_6D_6 of compound **1d** (reaction crude, isomer mixture detected).

(a)

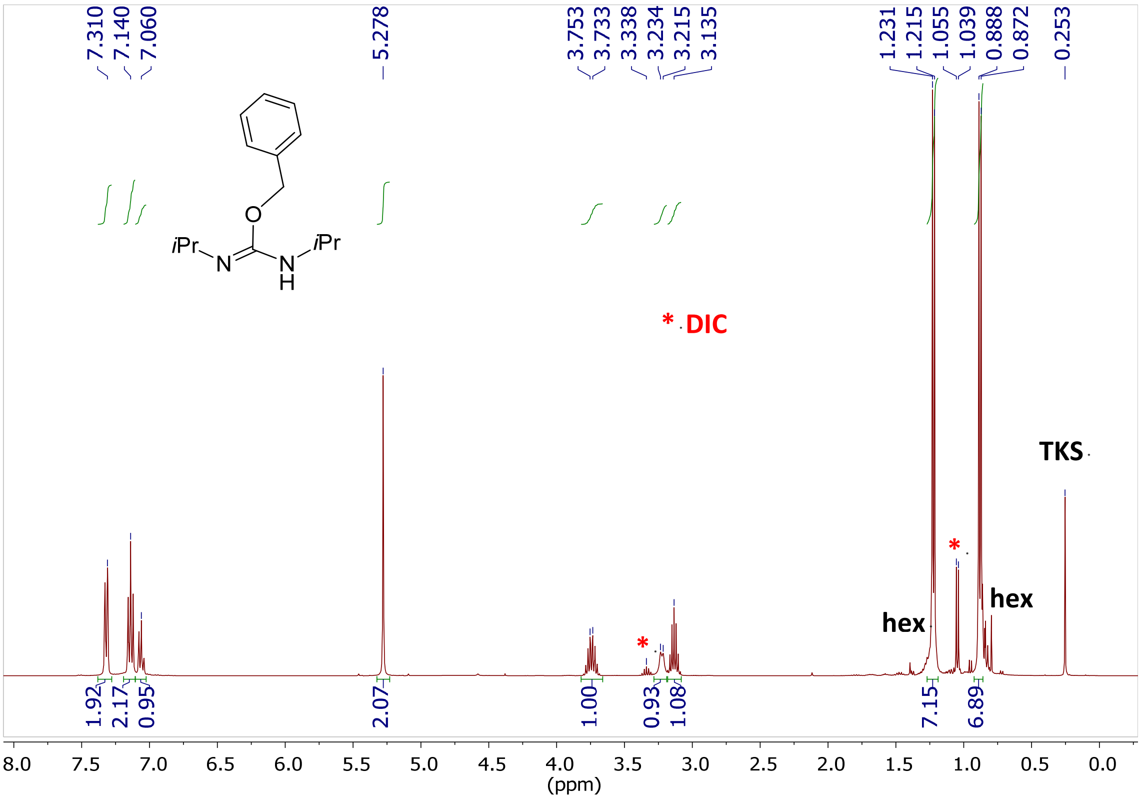

(b)

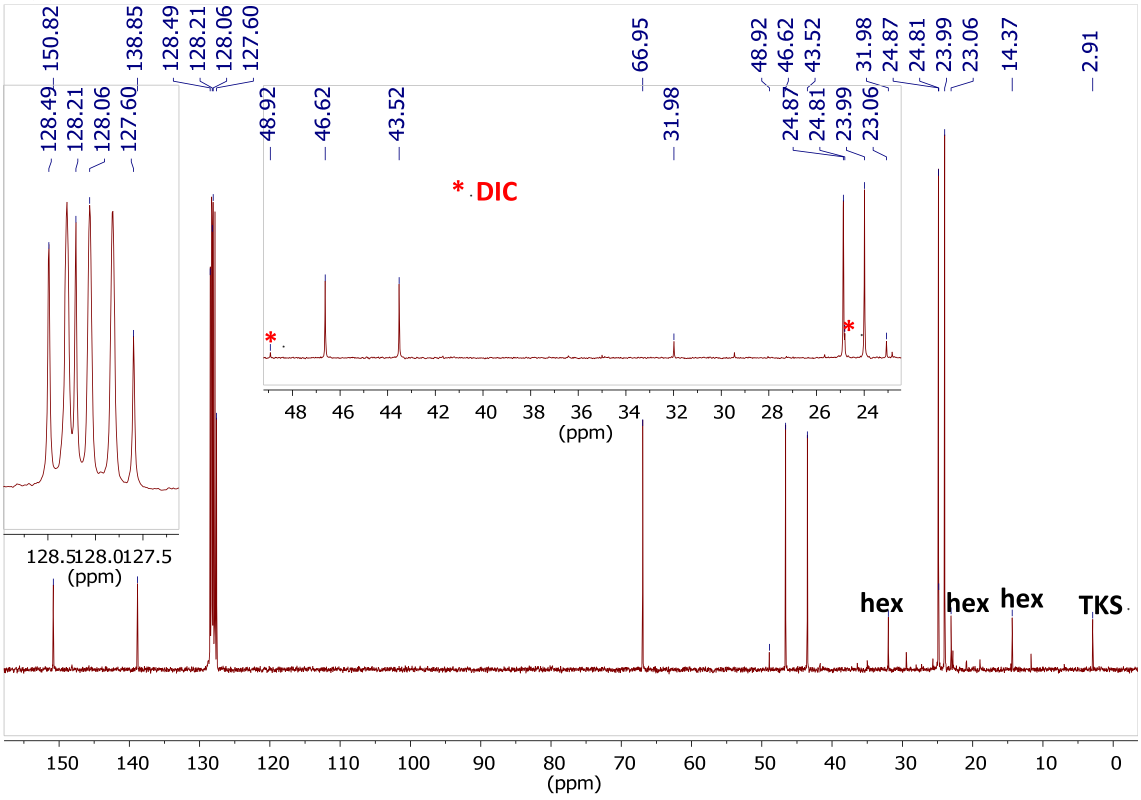

Figure S5. ^{1}H (a) and $^{13}C\{^{1}H\}$ (b) NMR in C_6D_6 of compound **1e** (reaction crude).

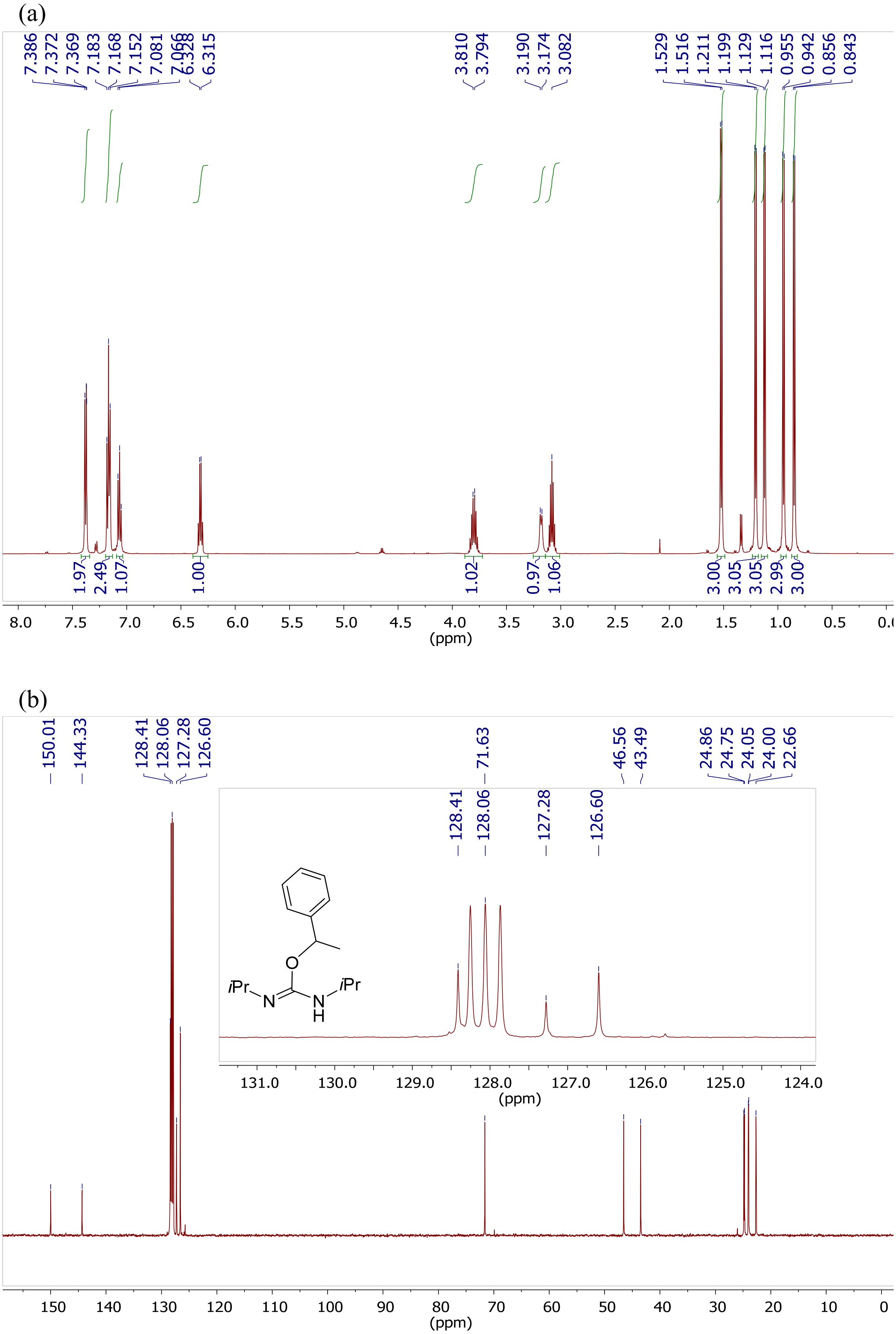

Figure S6. ^{1}H (a) and ^{13}C{^{1}H} (b) NMR in C_6D_6 of compound **1f** (isolated product).

(a)

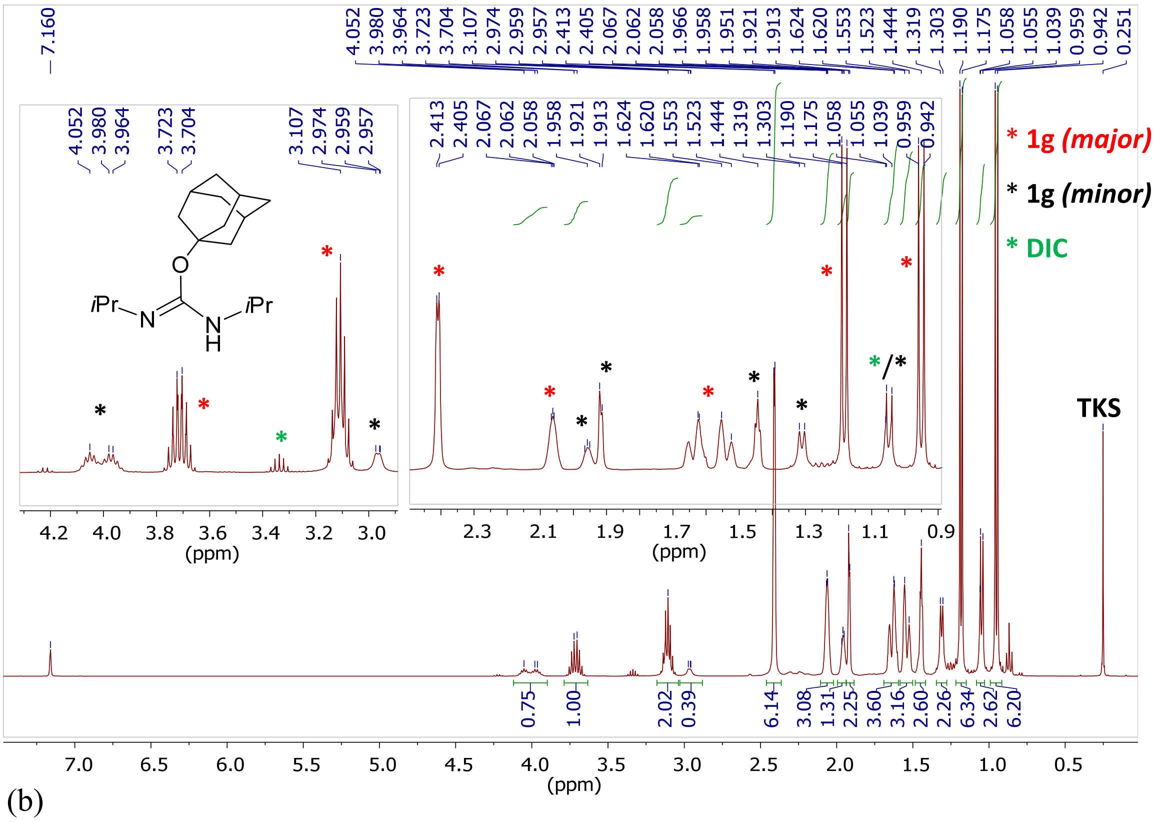

(b)

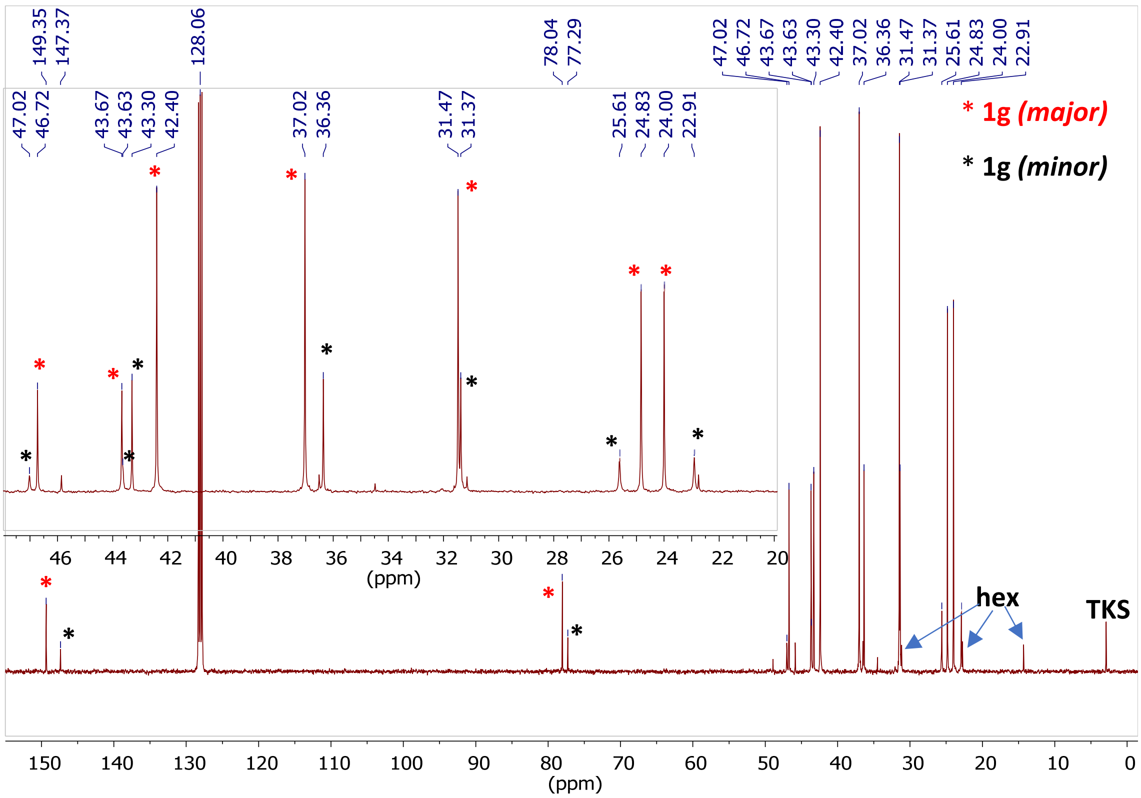

Figure S7. ^{1}H (a) and ^{13}C{^{1}H} (b) NMR in C_6D_6 of compound **1g** (reaction crude after solvent evaporation, isomer mixture detected).

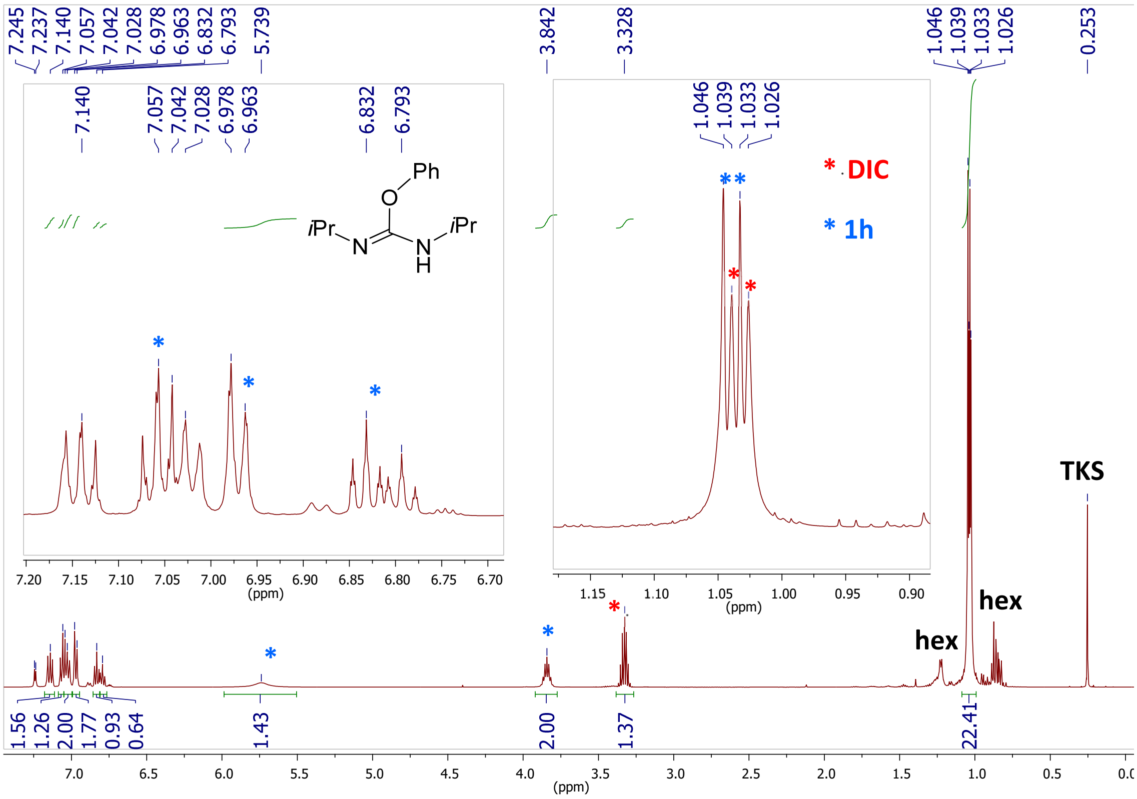

Figure S8. ^{1}H NMR in C_6D_6 of compound **1h** (reaction crude).

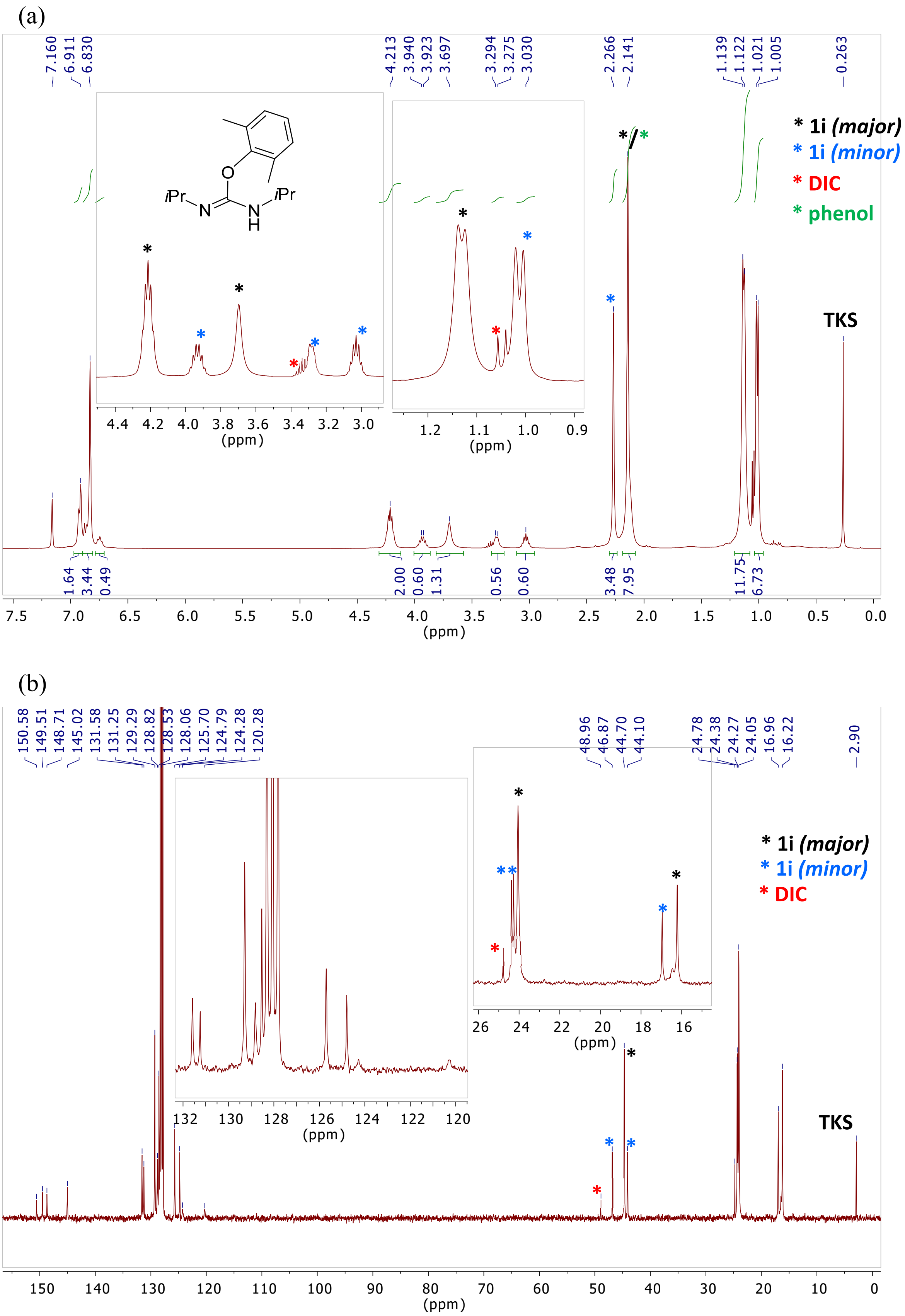

Figure S9. ^{1}H (a) and $^{13}C\{^{1}H\}$ (b) NMR in C_6D_6 of compound **1i** (reaction crude after solvent evaporation, isomer mixture detected).

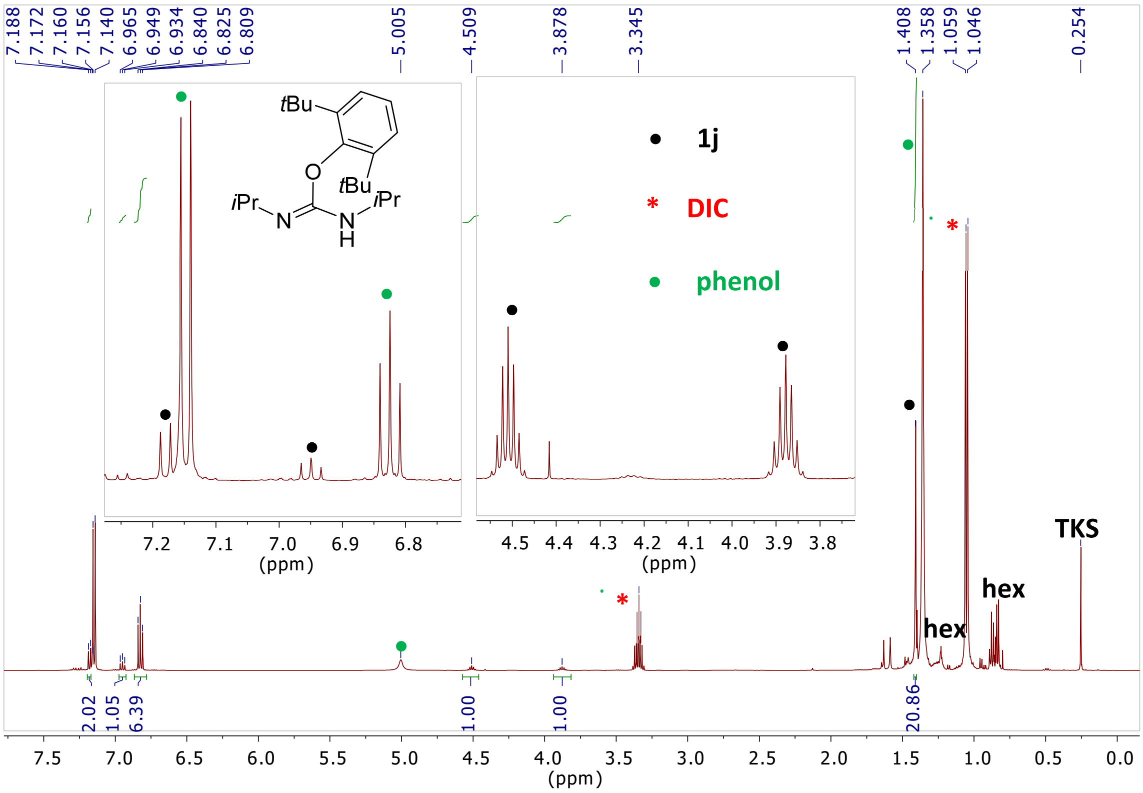

Figure S10. ^{1}H NMR in C_6D_6 of compound **1j** (reaction crude).

(a)

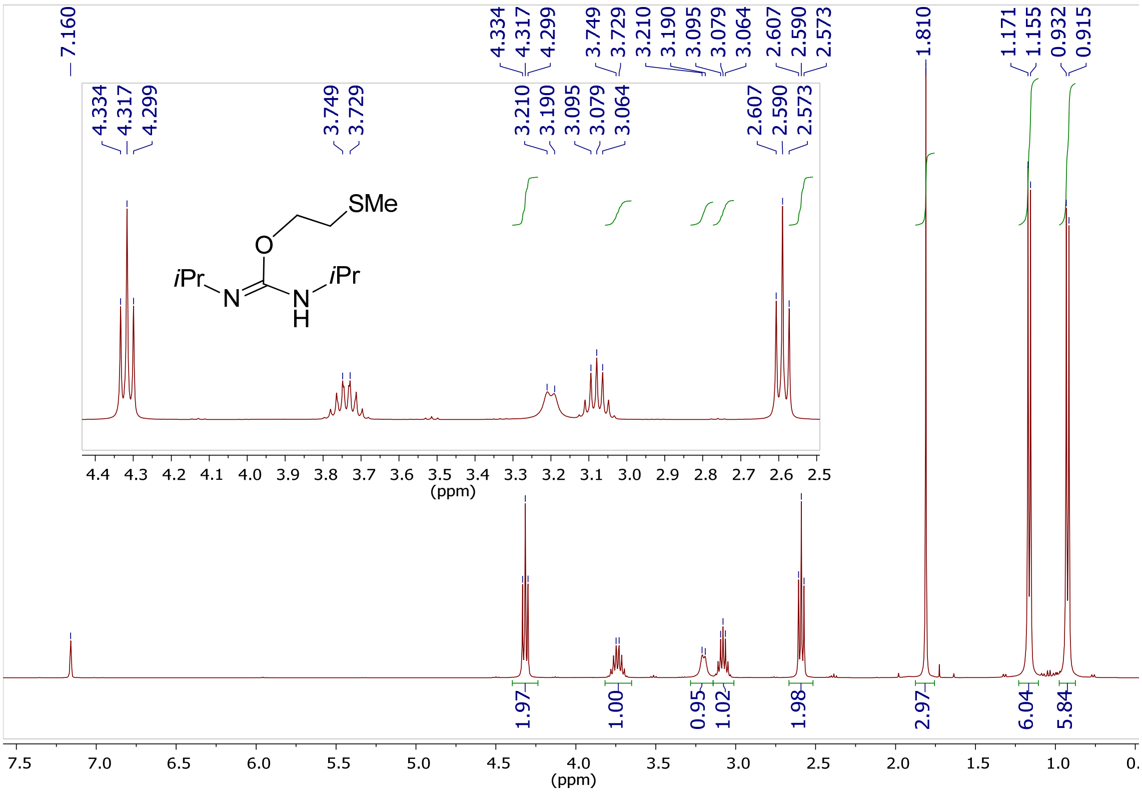

(b)

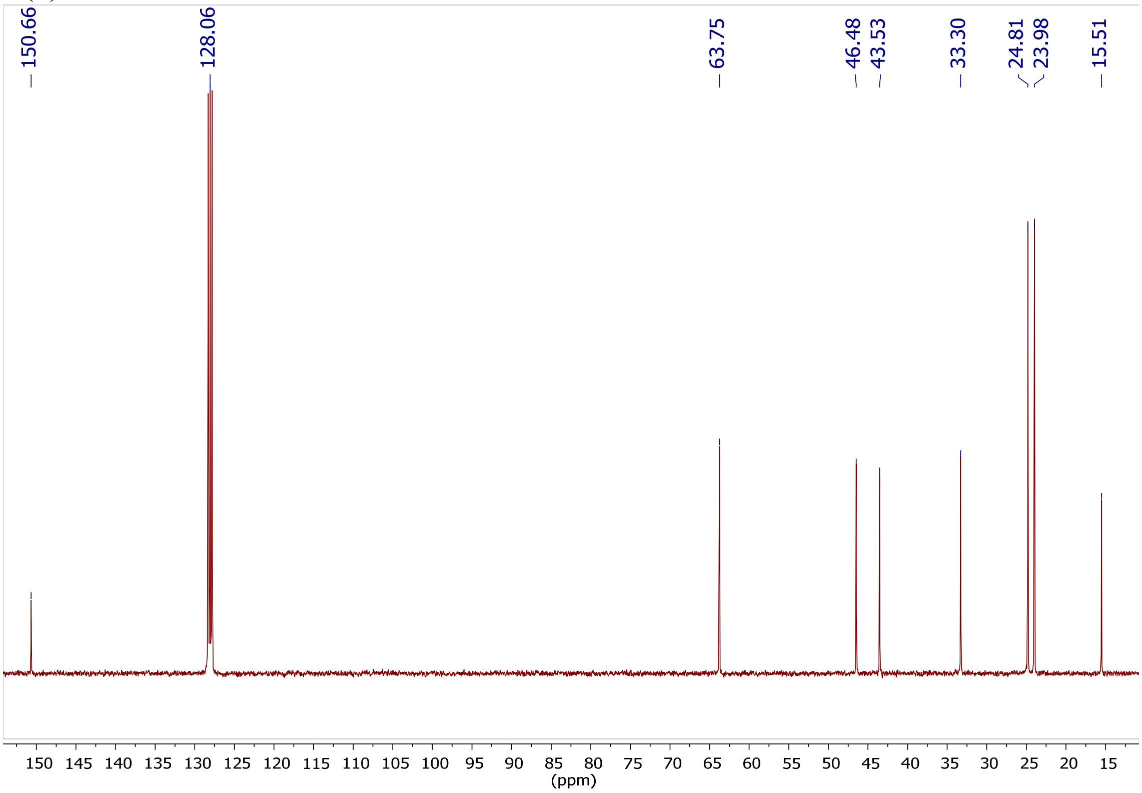

Figure S11. ^{1}H (a) and $^{13}C\{^{1}H\}$ (b) NMR in C_6D_6 of compound **1k** (isolated product).

(a)

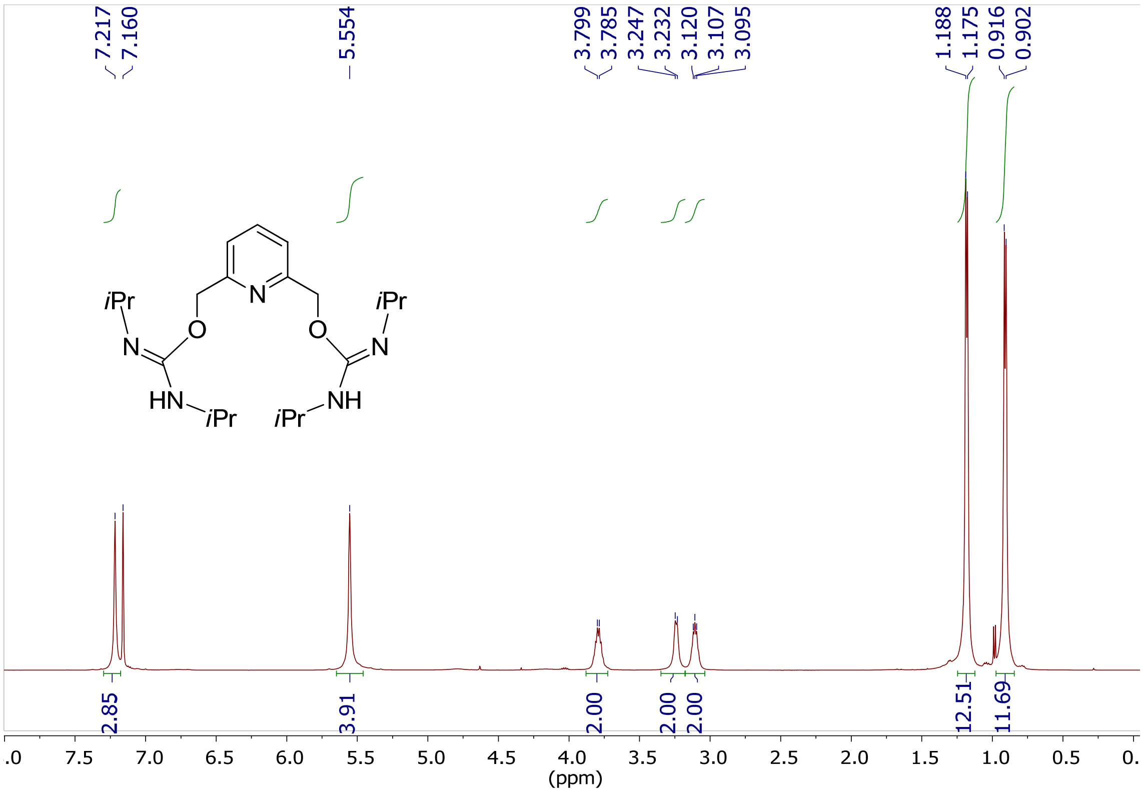

(b)

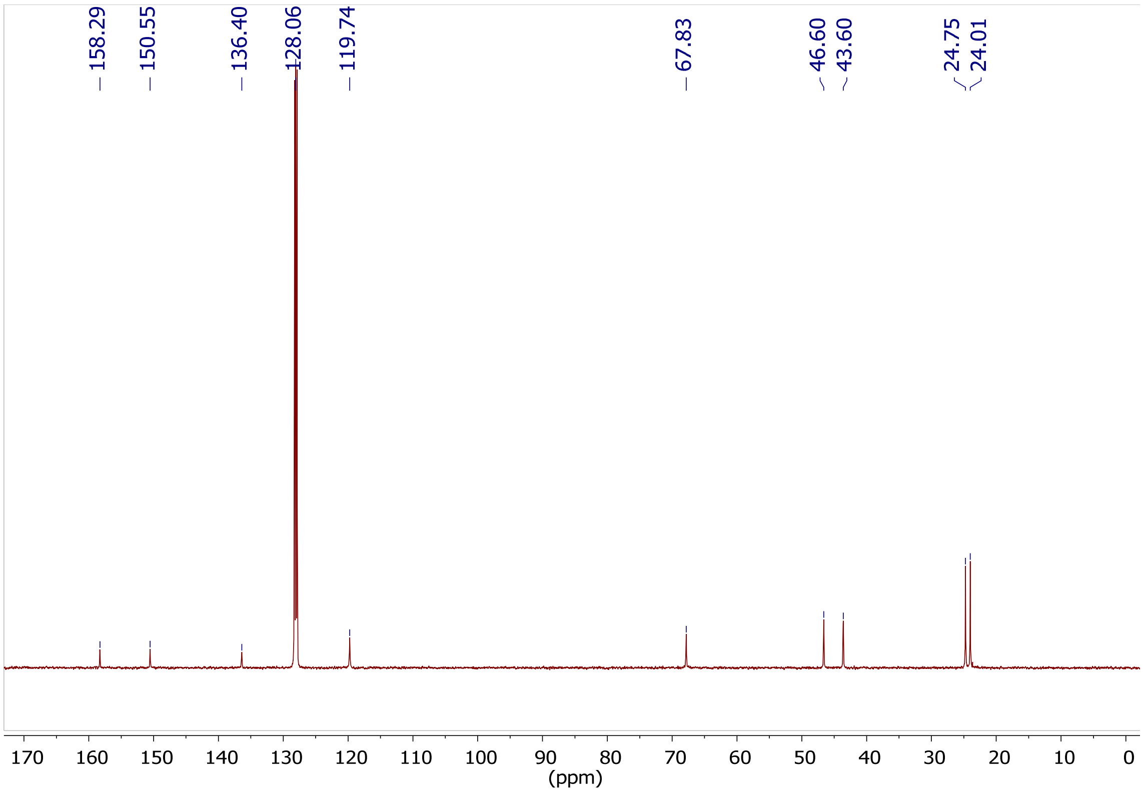

Figure S12. ^{1}H (a) and $^{13}C\{^{1}H\}$ (b) NMR in C_6D_6 of compound **1l** (isolated product).

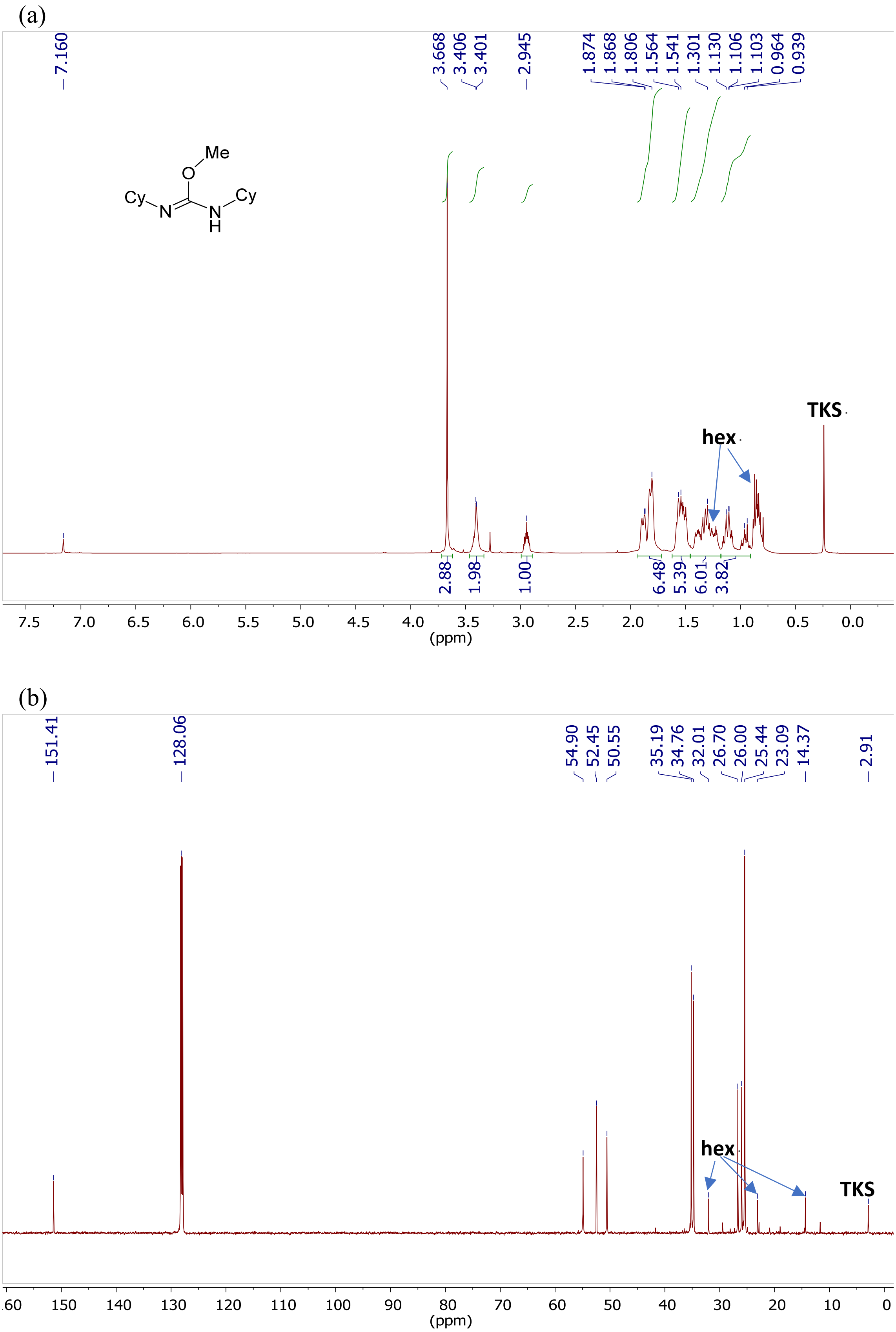

Figure S13. ^{1}H (a) and ^{13}C{^{1}H} (b) NMR in C_6D_6 of compound **2a** (reaction crude).

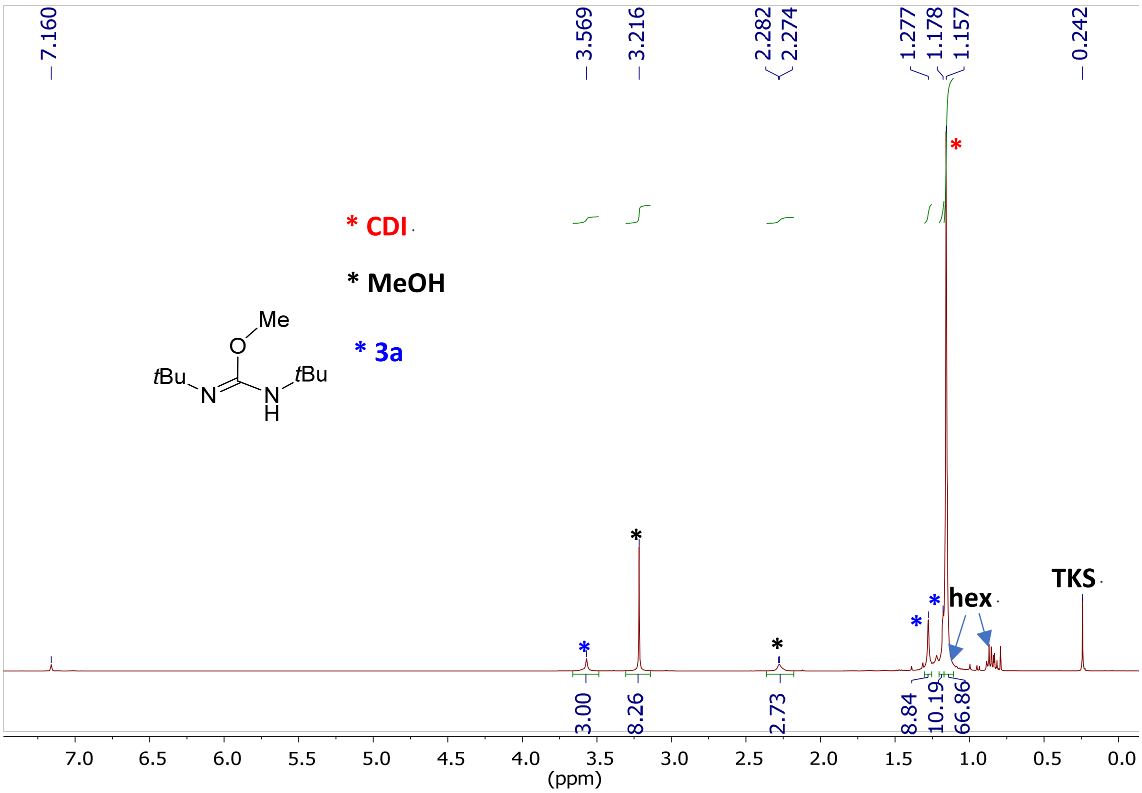

Figure S14. ^{1}H NMR in C_6D_6 of compound **3a** (reaction crude).

(a)

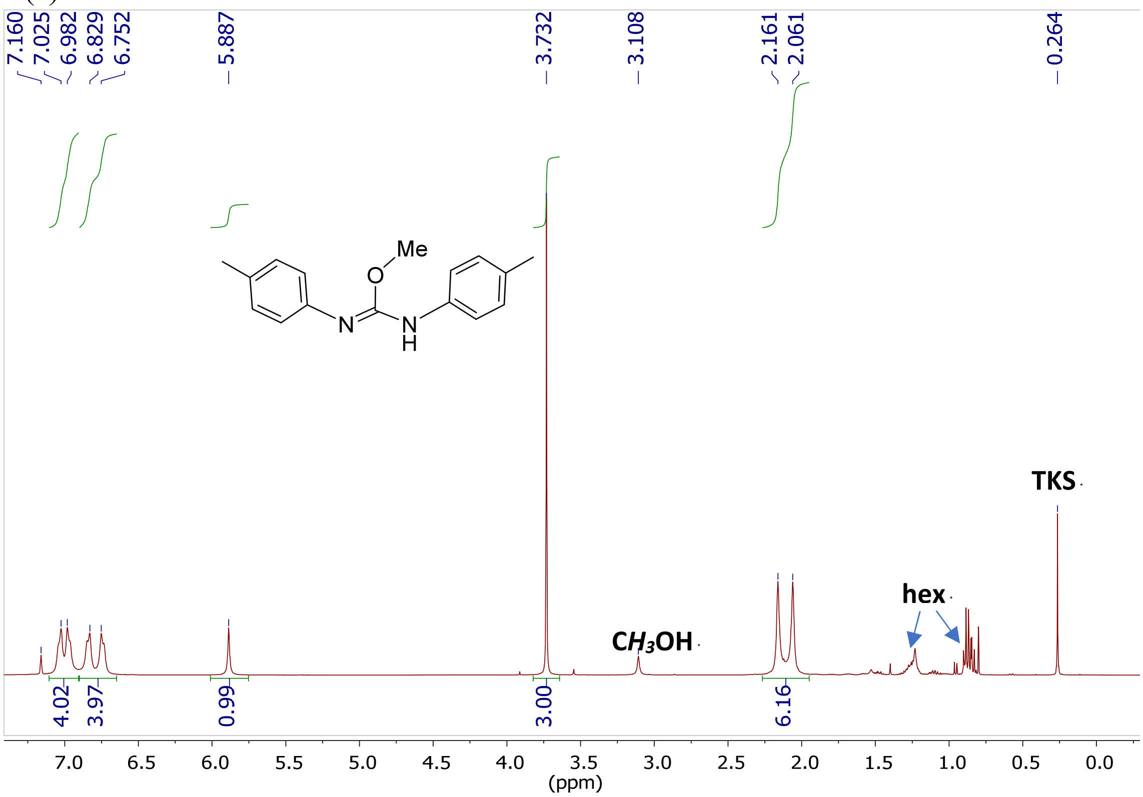

(b)

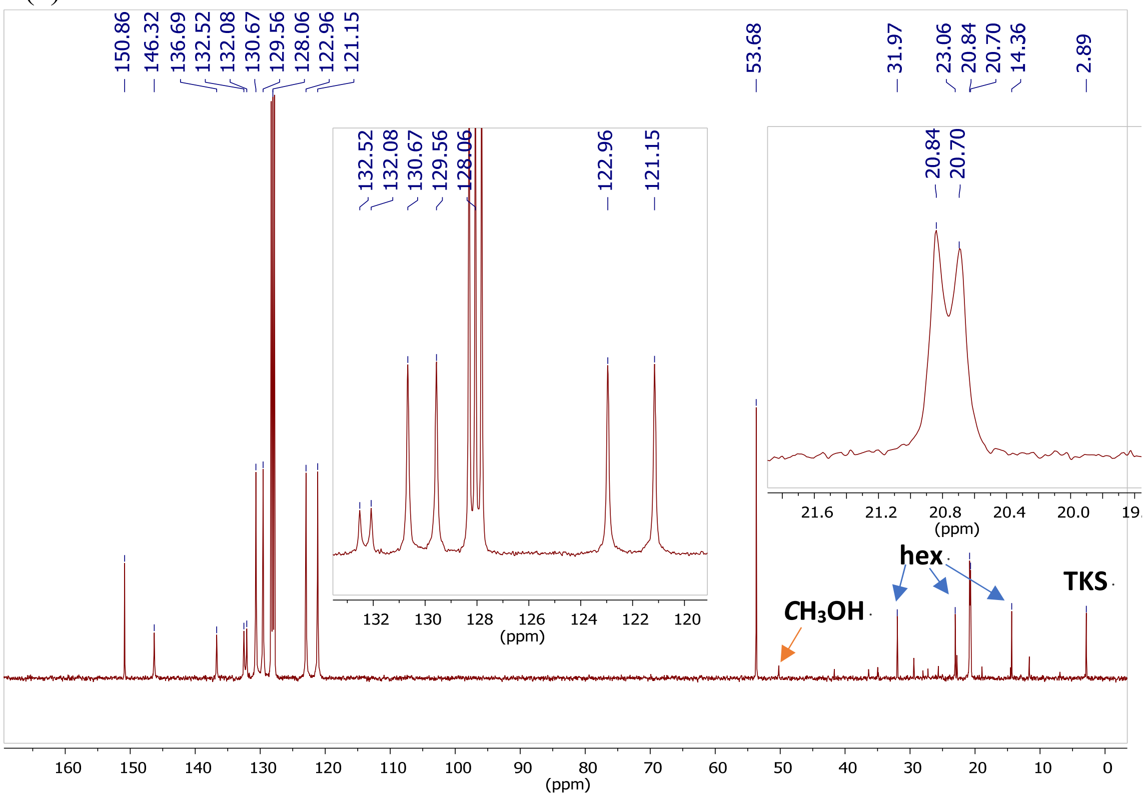

Figure S15. ^{1}H (a) and ^{13}C{^{1}H} (b) NMR in C_6D_6 of compound **4a** (reaction crude).

(a)

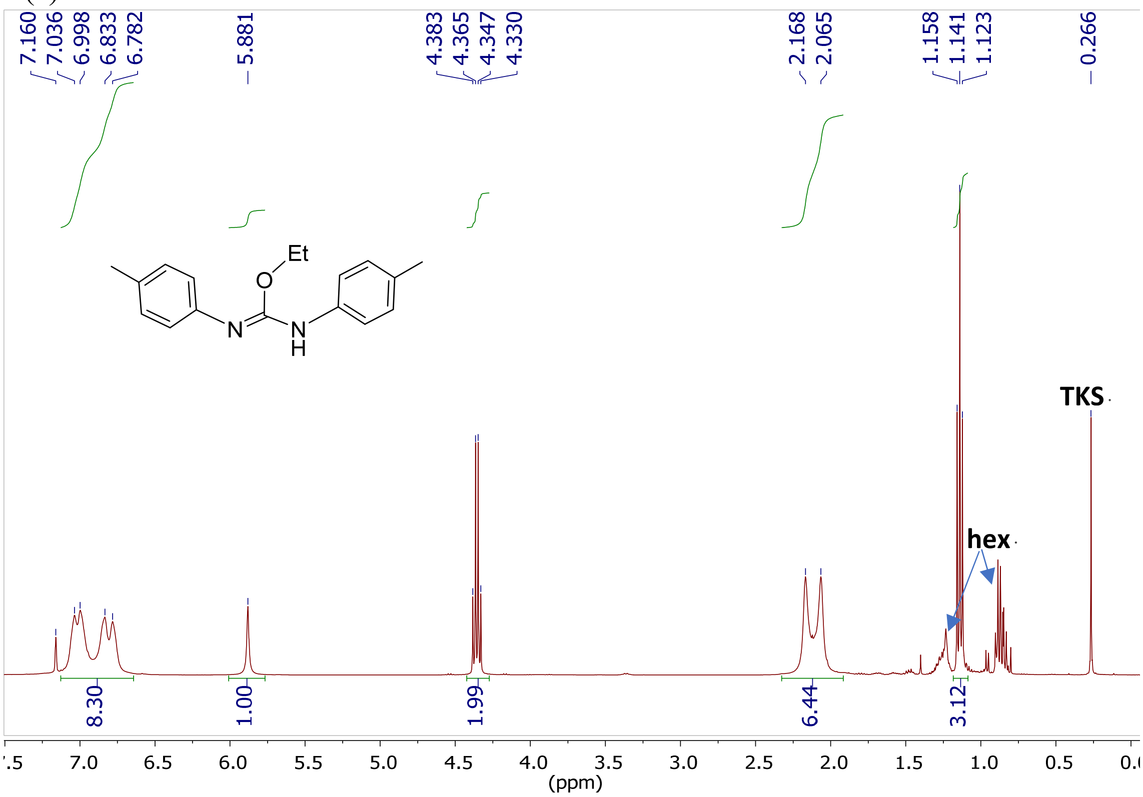

(b)

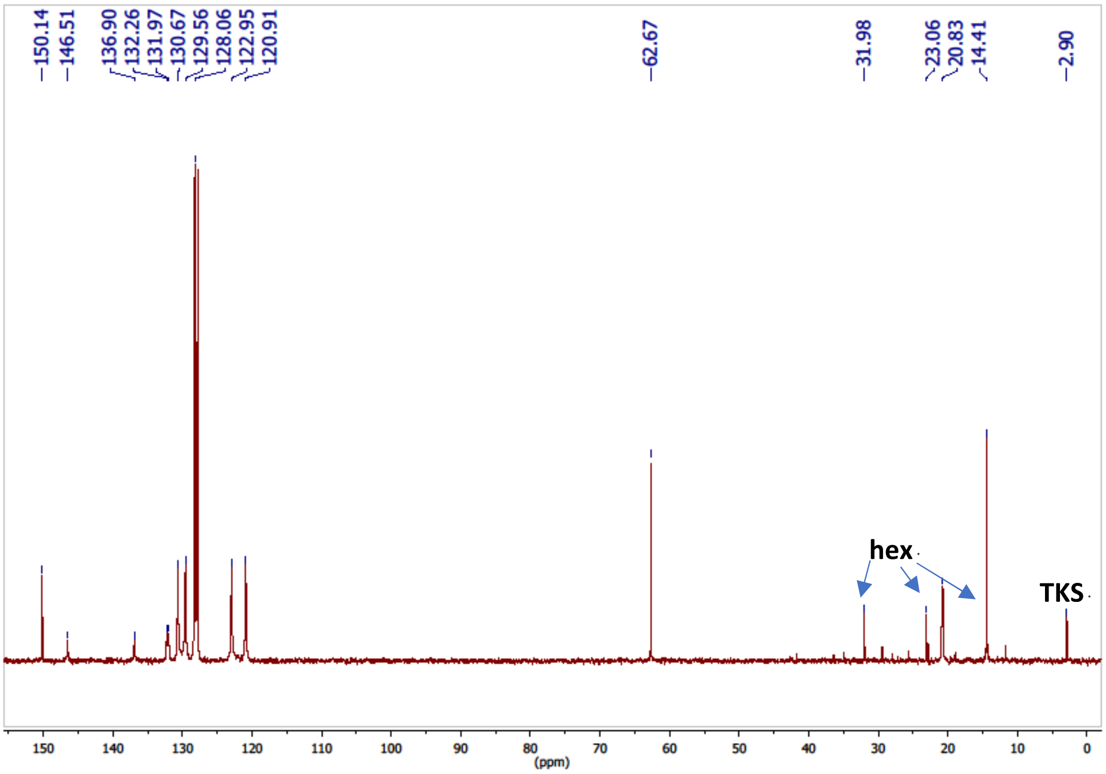

Figure S16. ^{1}H (a) and $^{13}C\{^{1}H\}$ (b) NMR in C_6D_6 of compound **4b** (reaction crude).

(a)

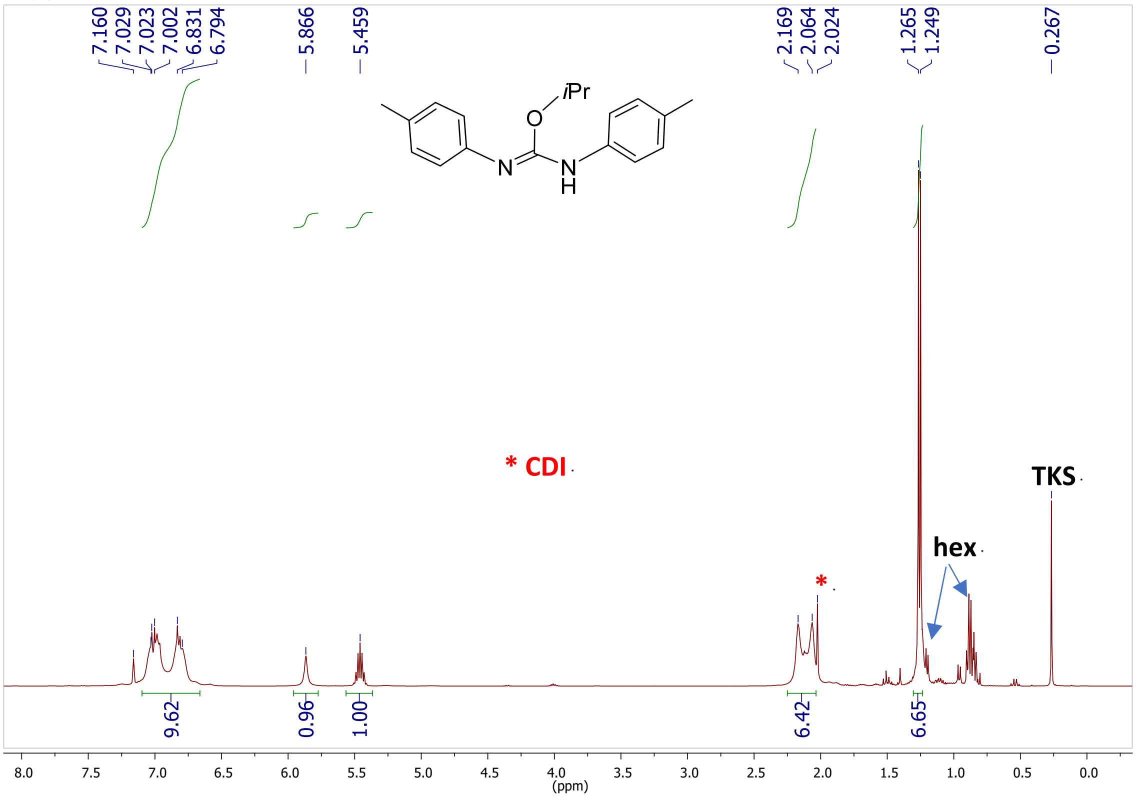

(b)

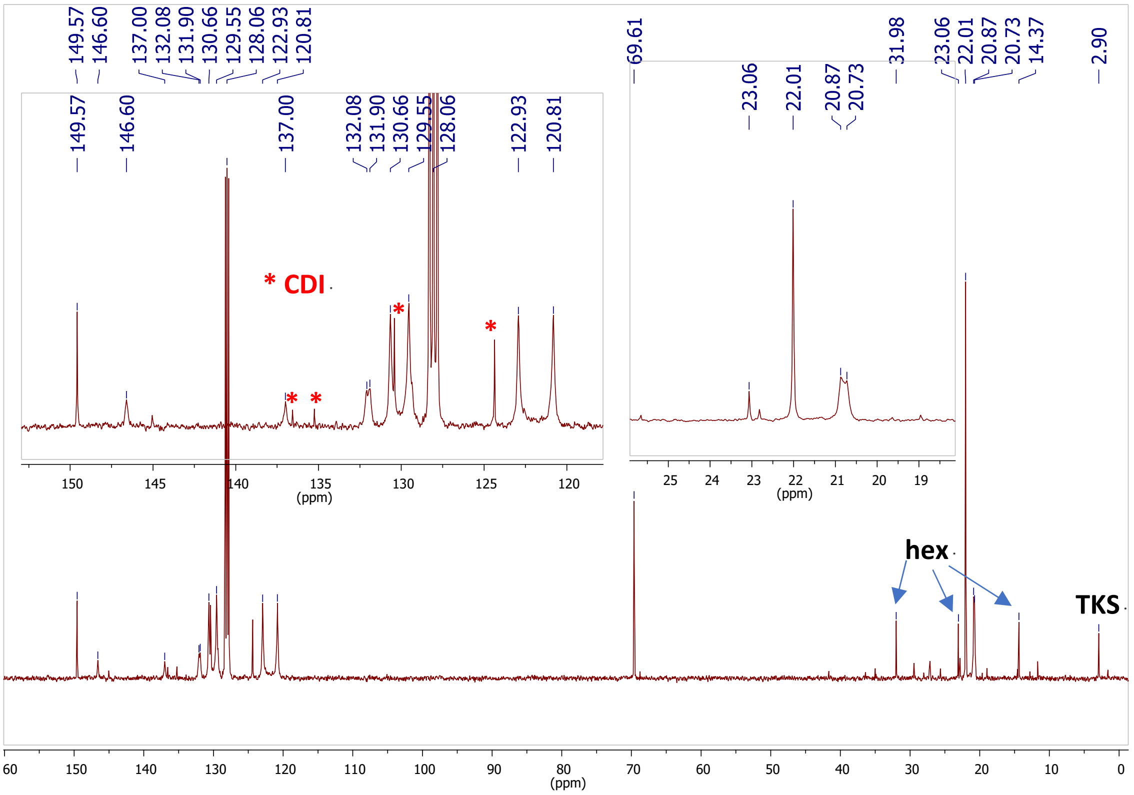

Figure S17. ^{1}H (a) and $^{13}C\{^{1}H\}$ (b) NMR in C_6D_6 of compound **4c** (reaction crude).

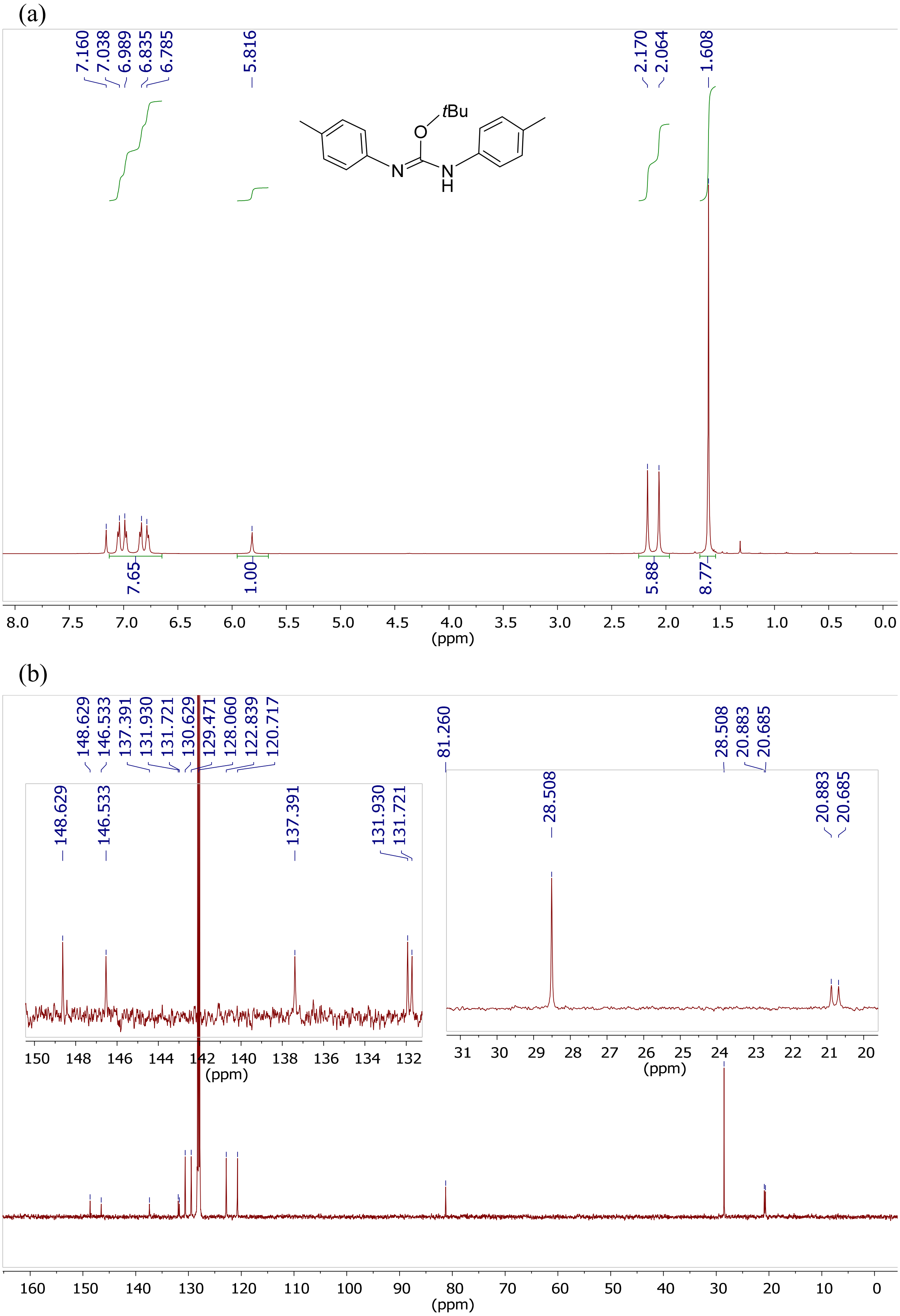

Figure S18. ^{1}H (a) and $^{13}C\{^{1}H\}$ (b) NMR in C_6D_6 of compound **4d** (isolated product).

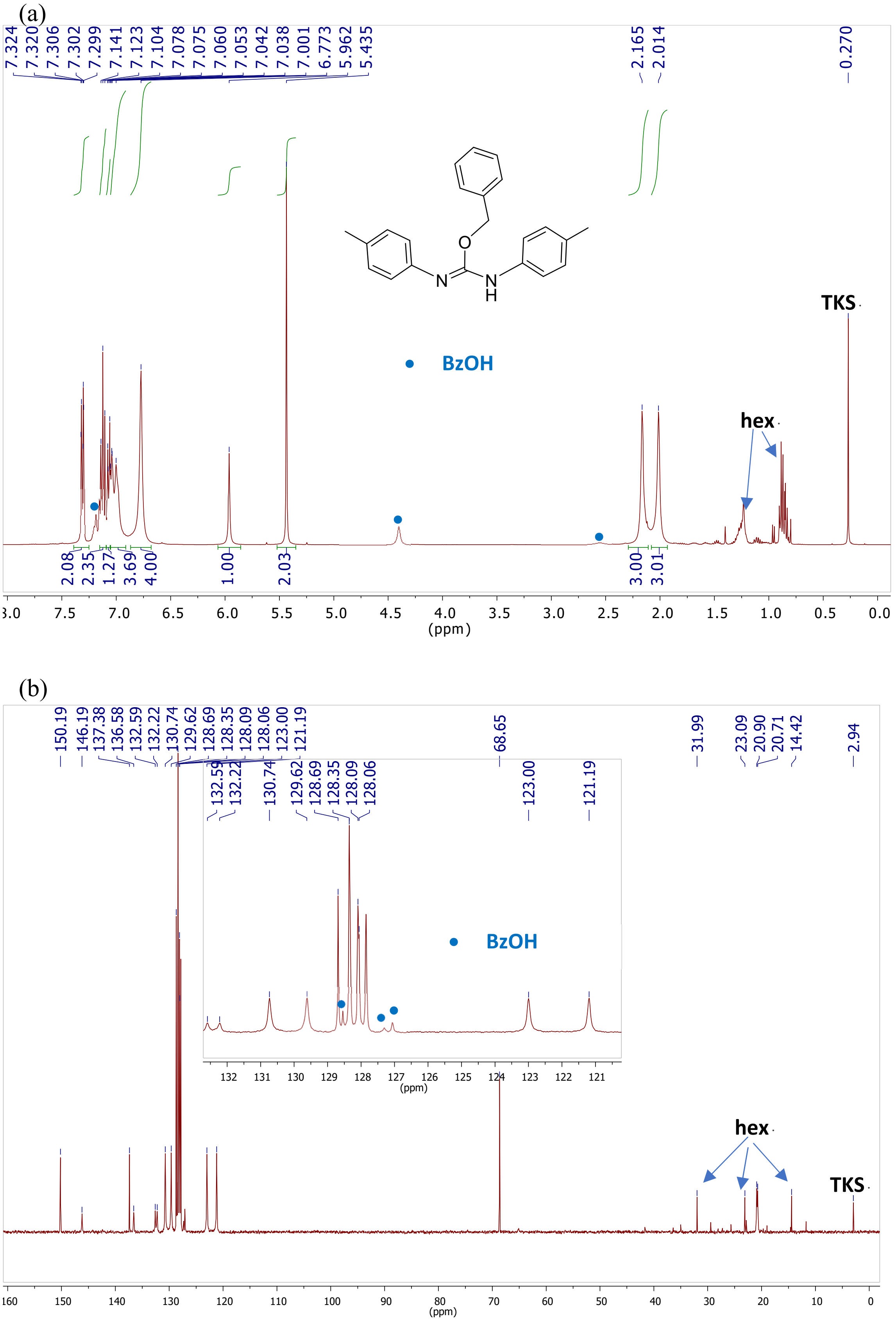

Figure S19. ^{1}H (a) and $^{13}C\{^{1}H\}$ (b) NMR in C_6D_6 of compound **4e** (reaction crude).

(a)

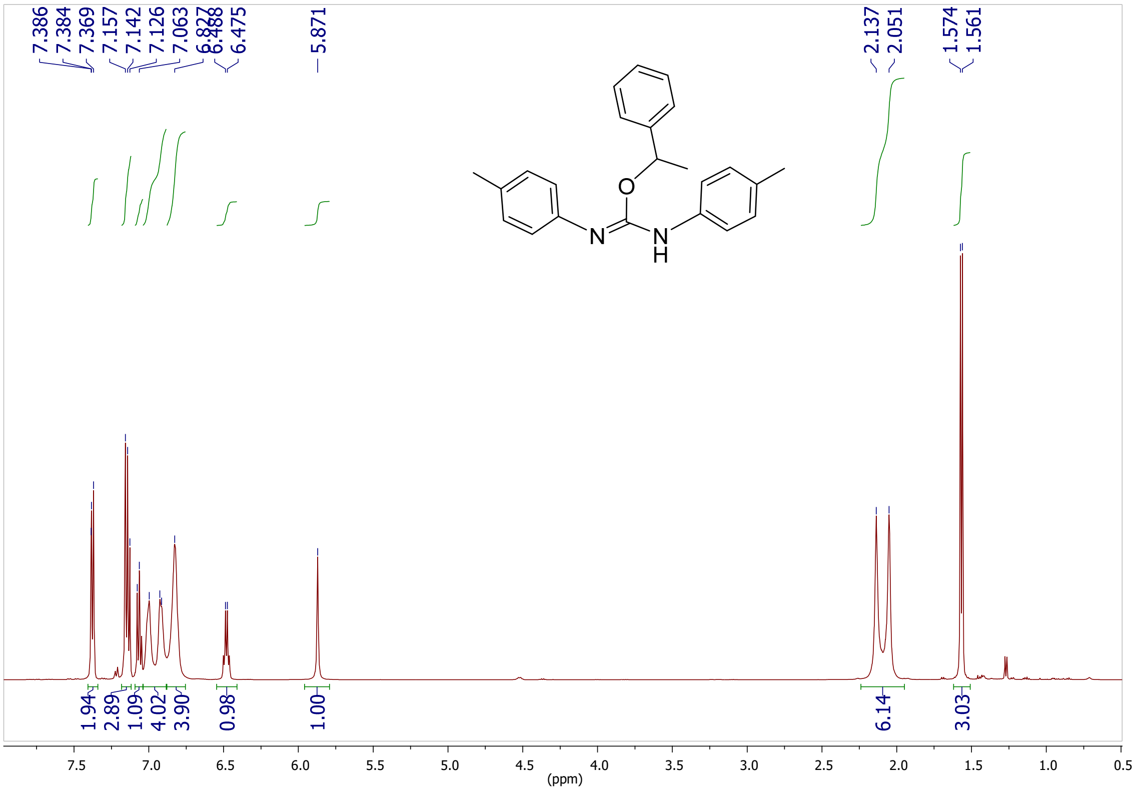

(b)

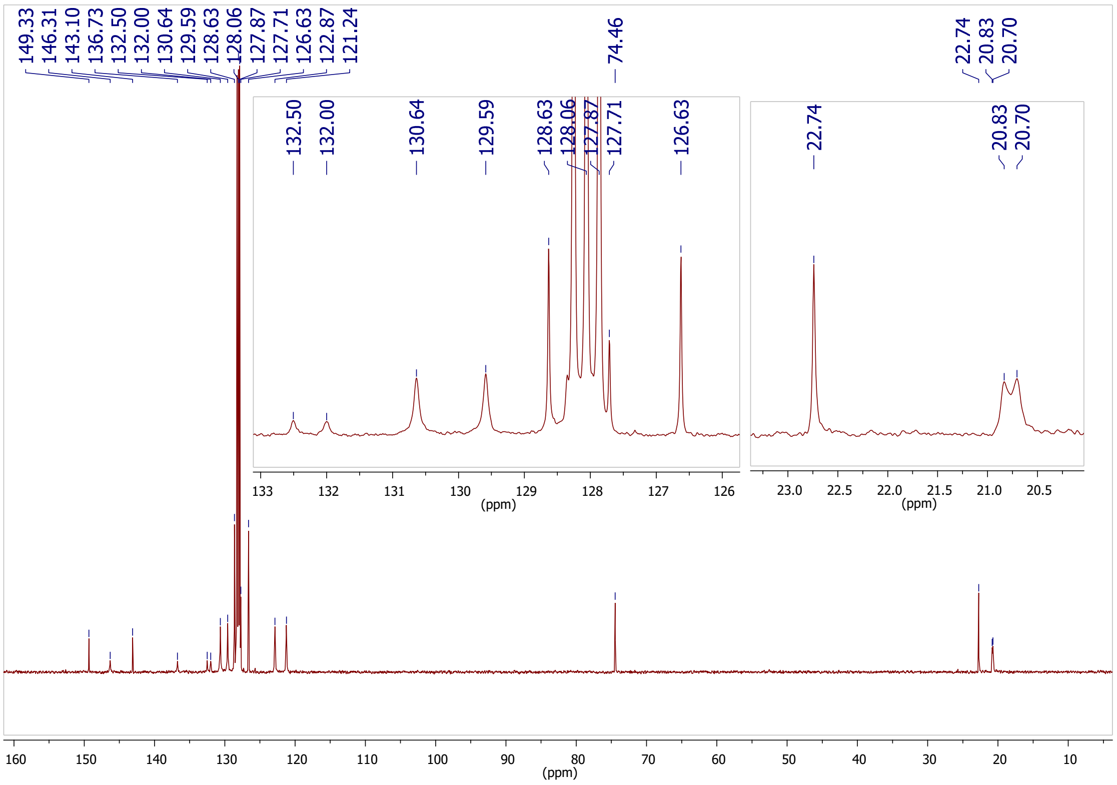

Figure S20. ^{1}H (a) and ^{13}C{^{1}H} (b) NMR in C_6D_6 of compound **4f** (isolated product).

(a)

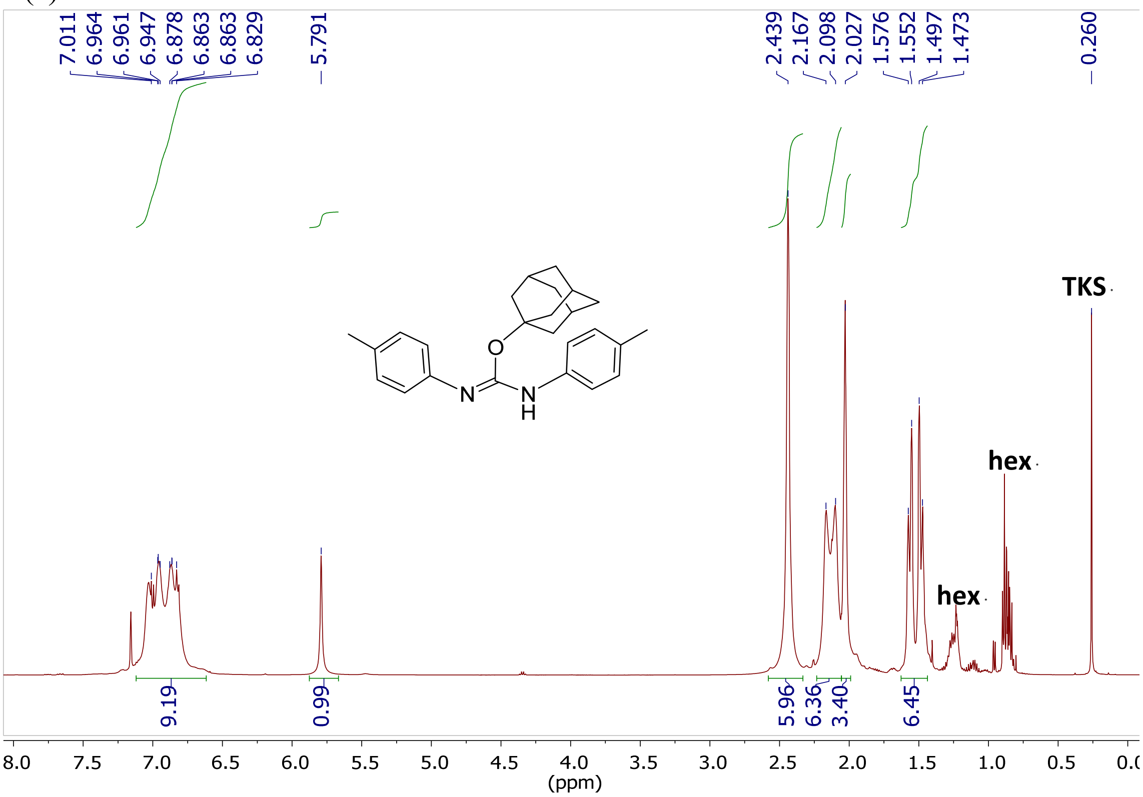

(b)

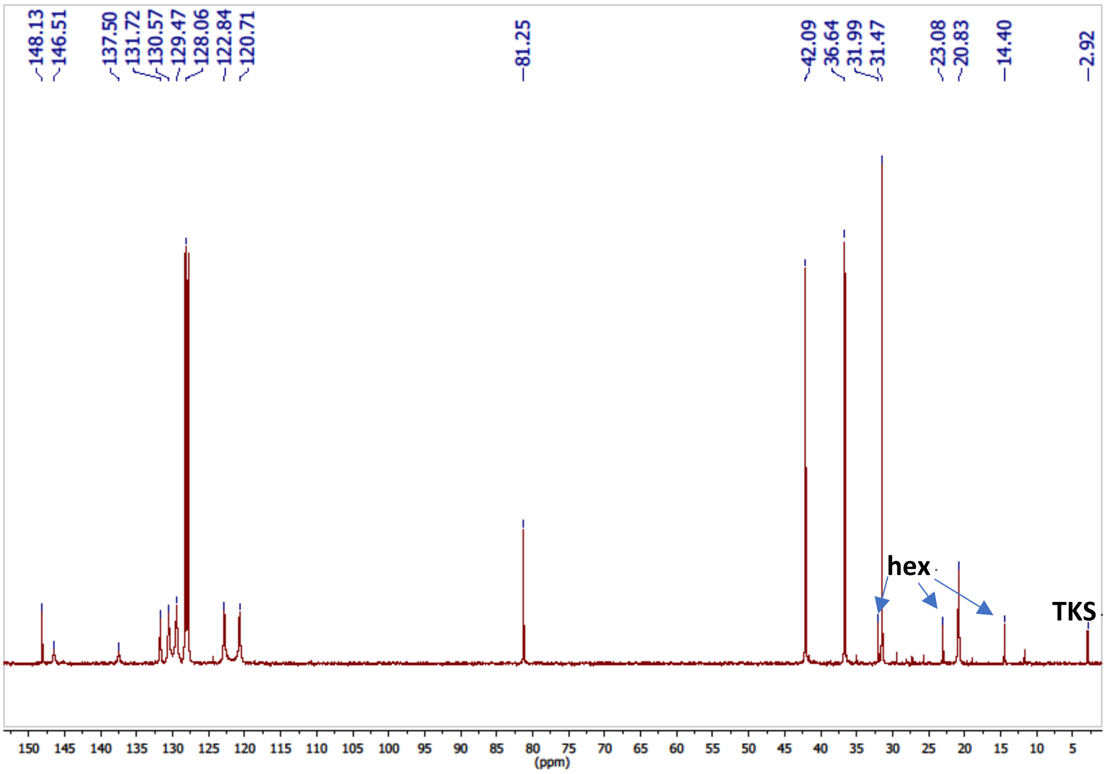

Figure S21. ^{1}H (a) and $^{13}C\{^{1}H\}$ (b) NMR in C_6D_6 of compound **4g** (reaction crude).

(a)

(b)

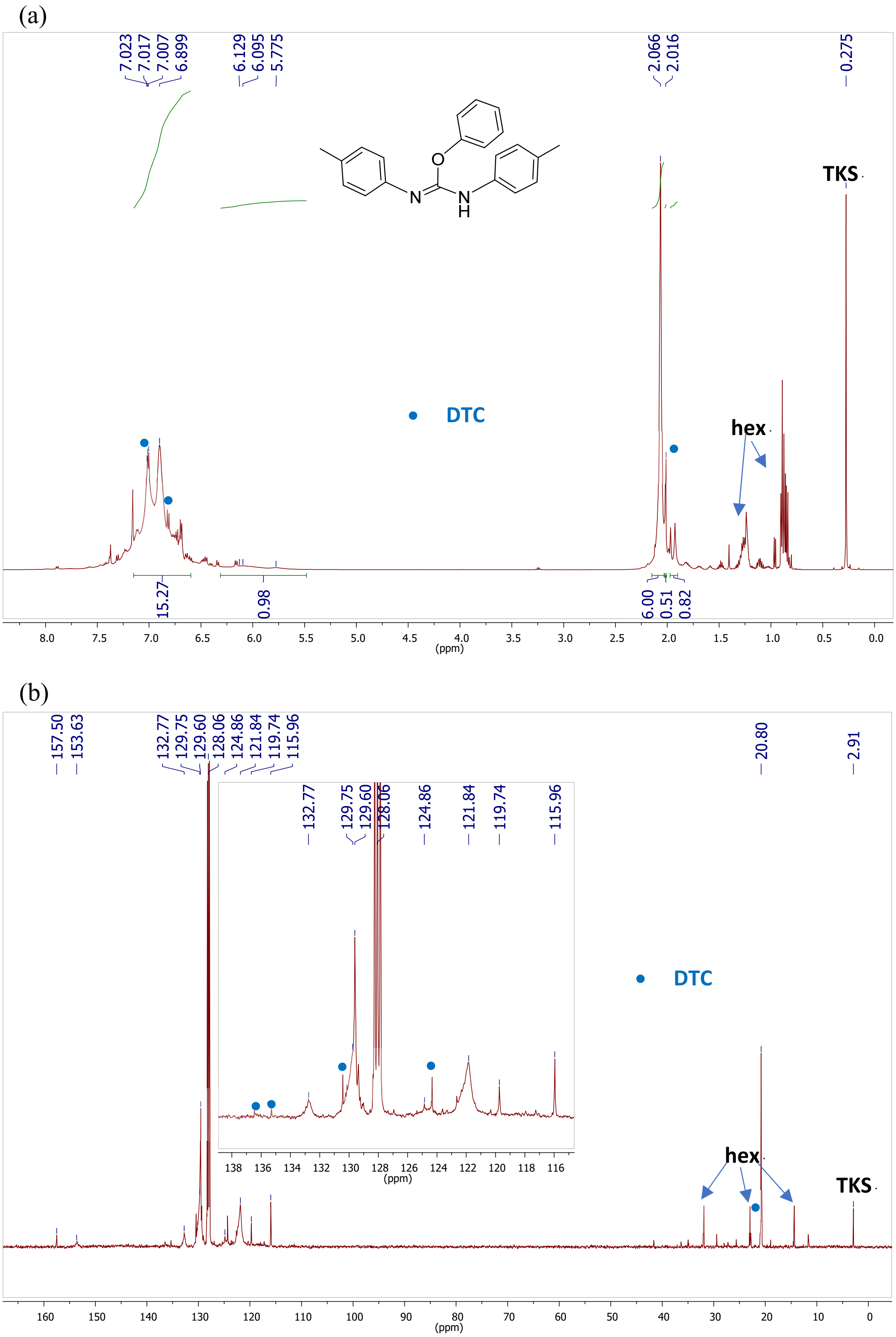

Figure S22. ^{1}H (a) and ^{13}C{^{1}H} (b) NMR in C_6D_6 of compound **4h** (reaction crude).

(a)

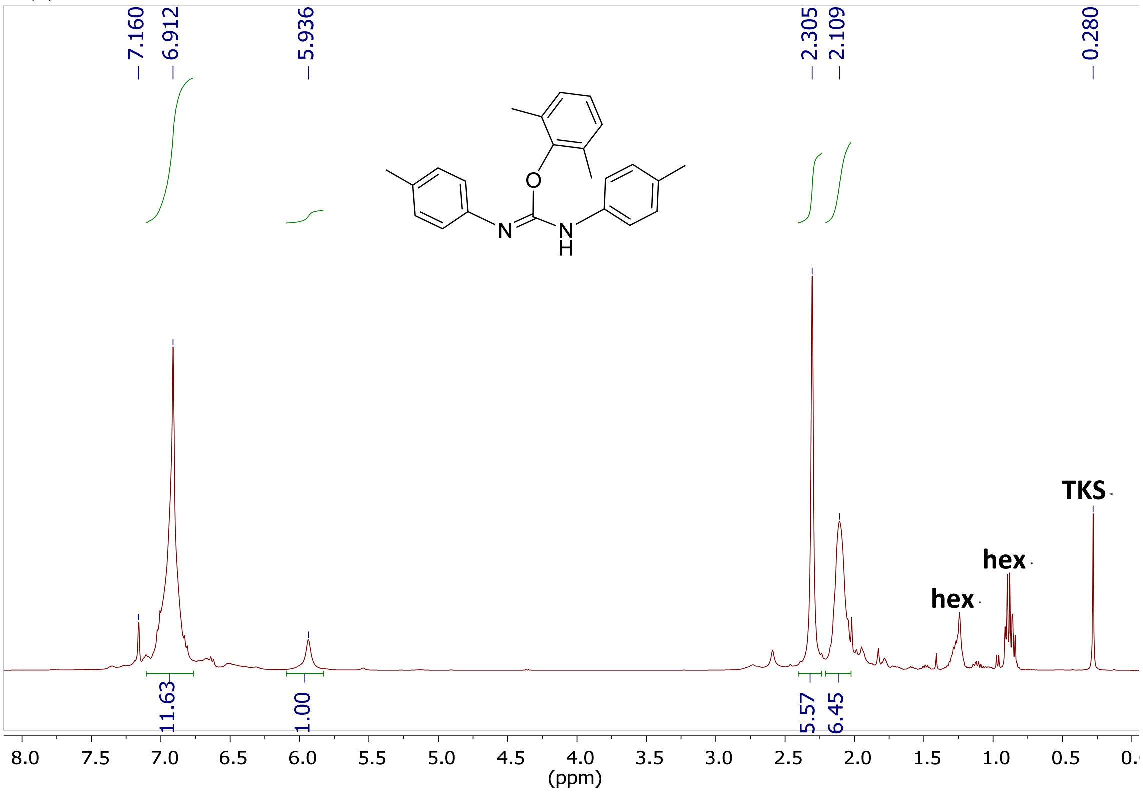

(b)

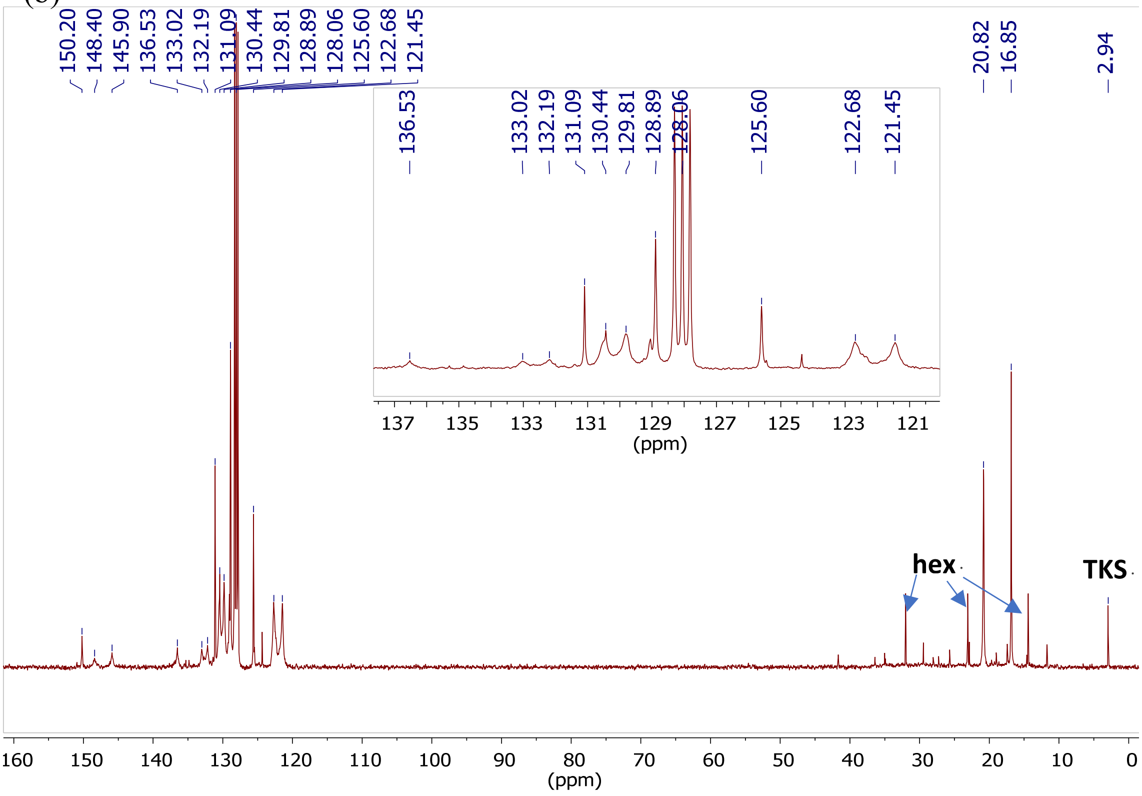

Figure S23. ^{1}H (a) and ^{13}C{^{1}H} (b) NMR in C_6D_6 of compound **4i** (reaction crude).

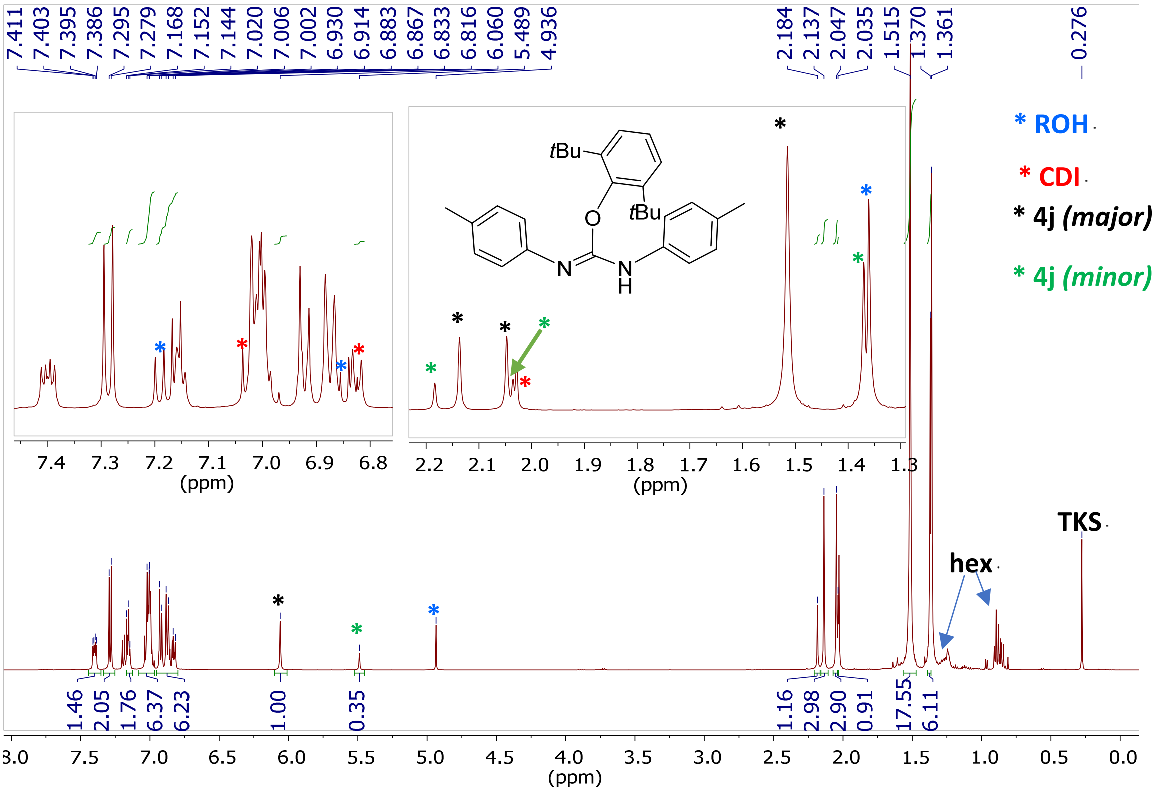

Figure S24. ^{1}H NMR in C_6D_6 of compound **4j** (reaction crude, isomer mixture).
[R = 2,6-*t*Bu_2-C_6H_3]

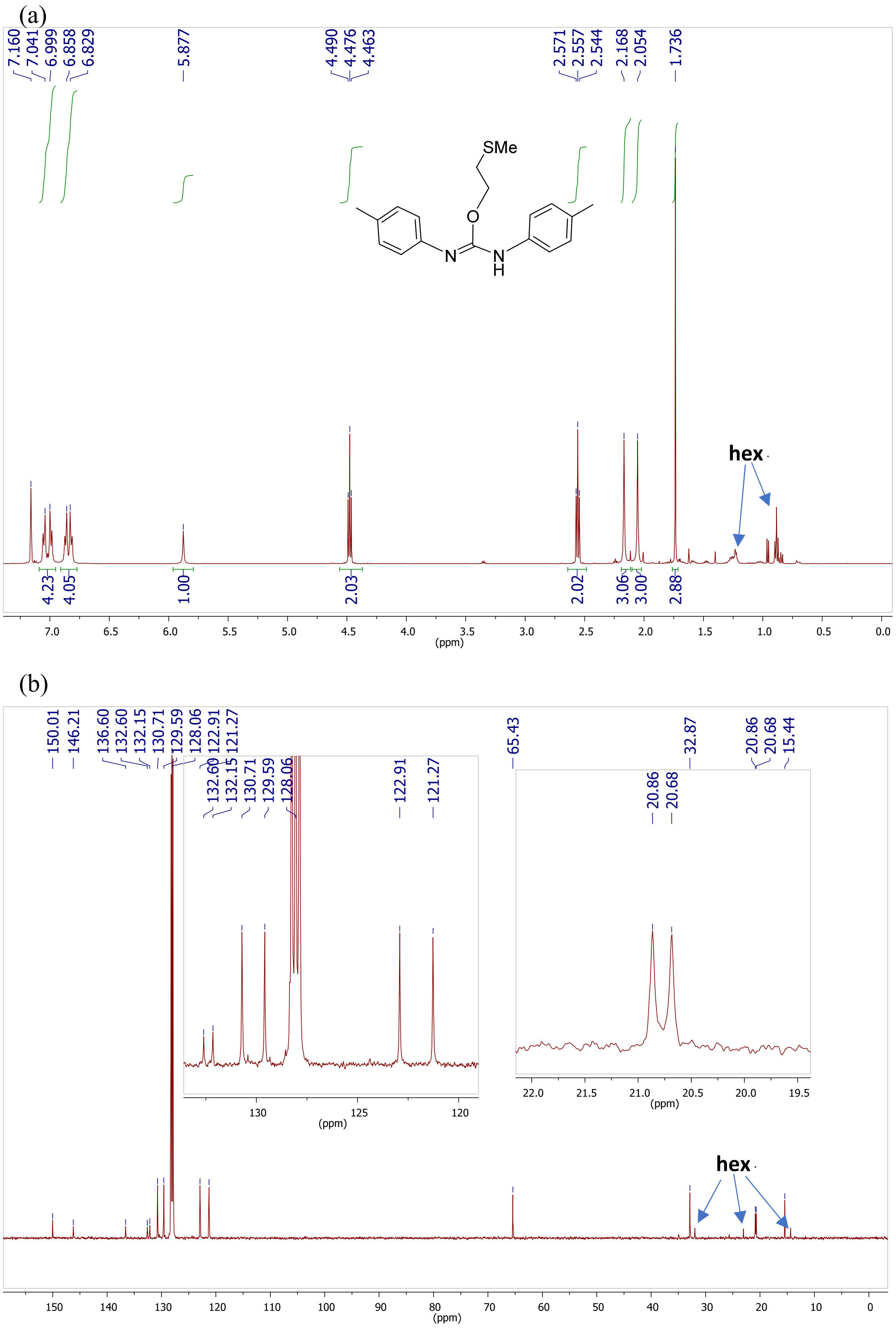

Figure S25. ^{1}H (a) and ^{13}C{^{1}H} (b) NMR in C_6D_6 of compound **4k** (isolated product).

(a)

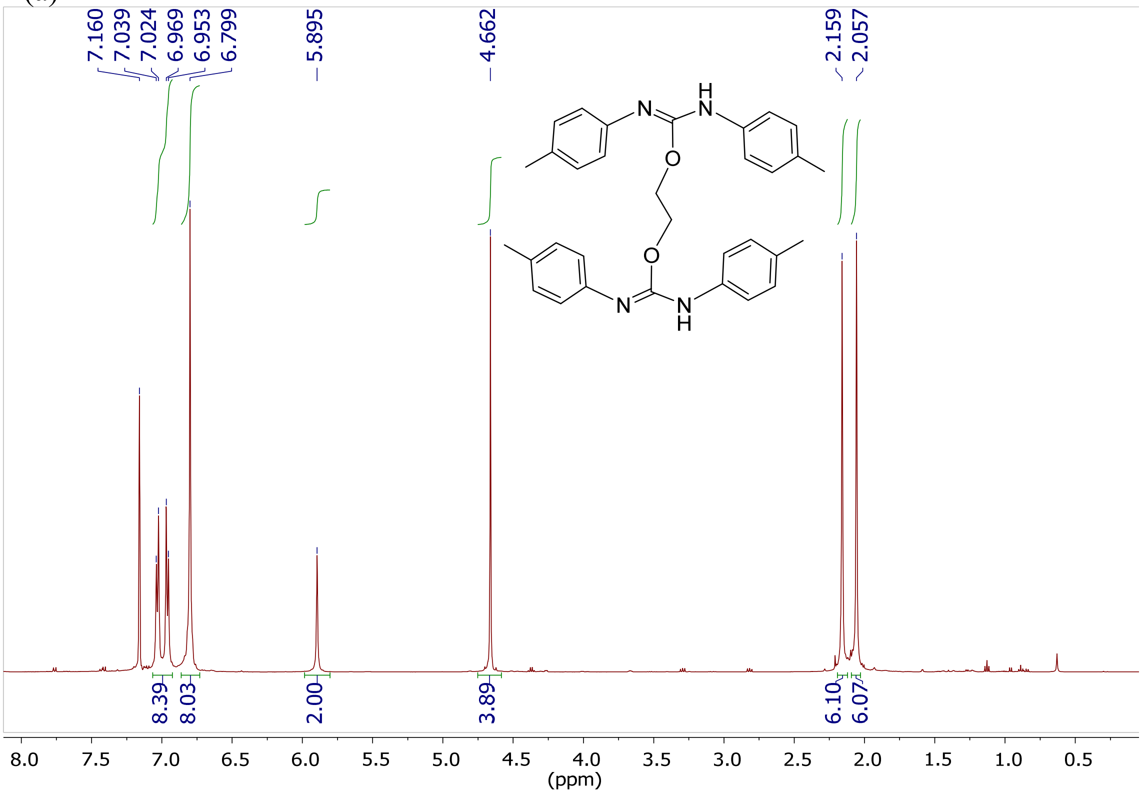

(b)

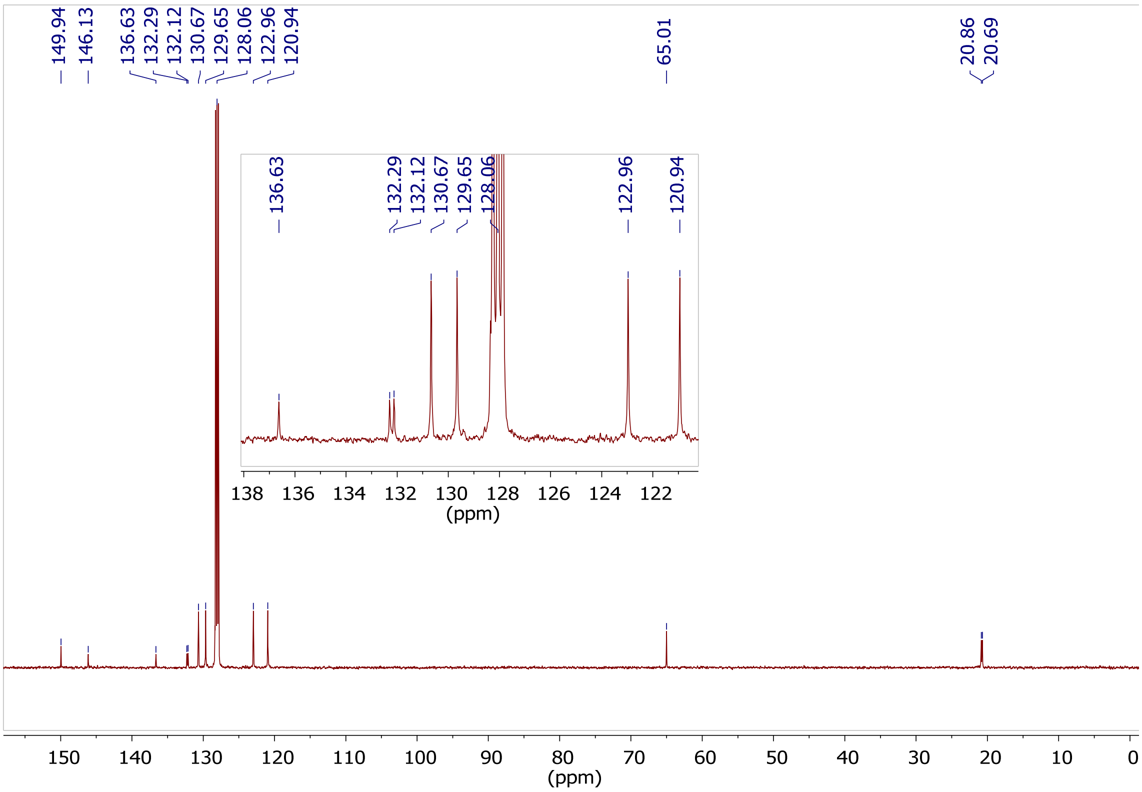

Figure S26. ^{1}H (a) and $^{13}C\{^{1}H\}$ (b) NMR in C_6D_6 of compound **4l** (isolated product).

(a)

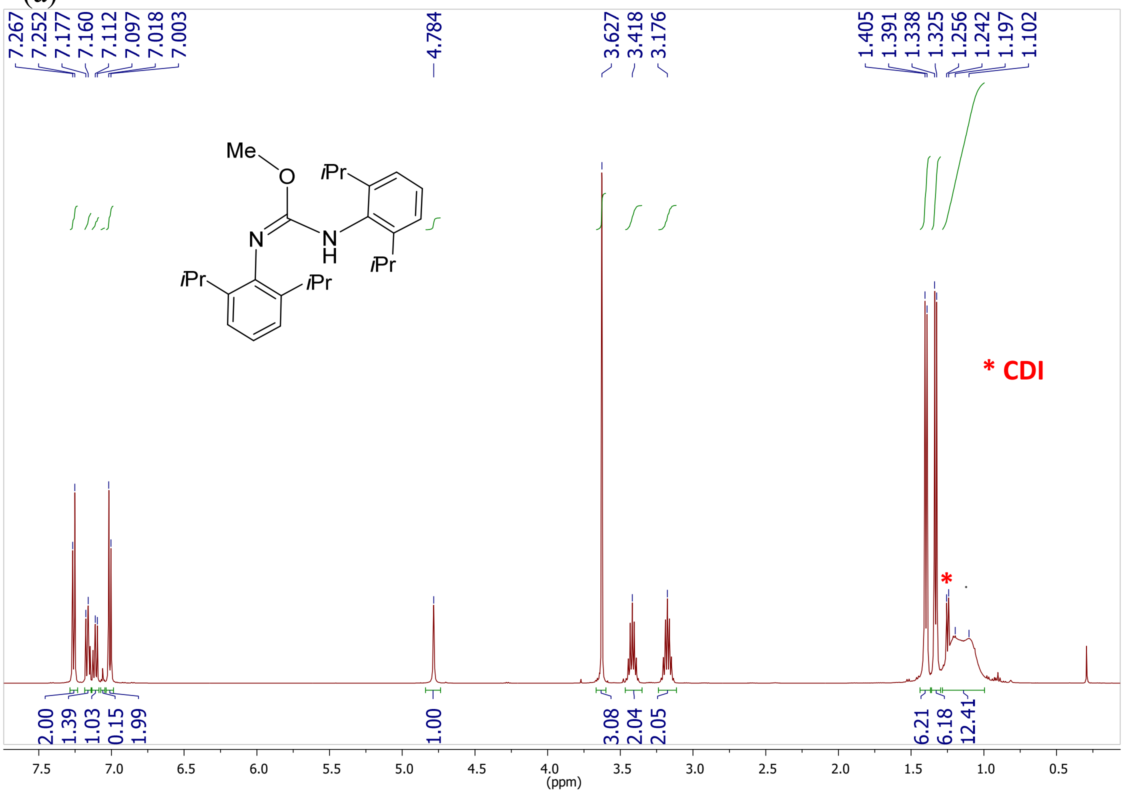

(b)

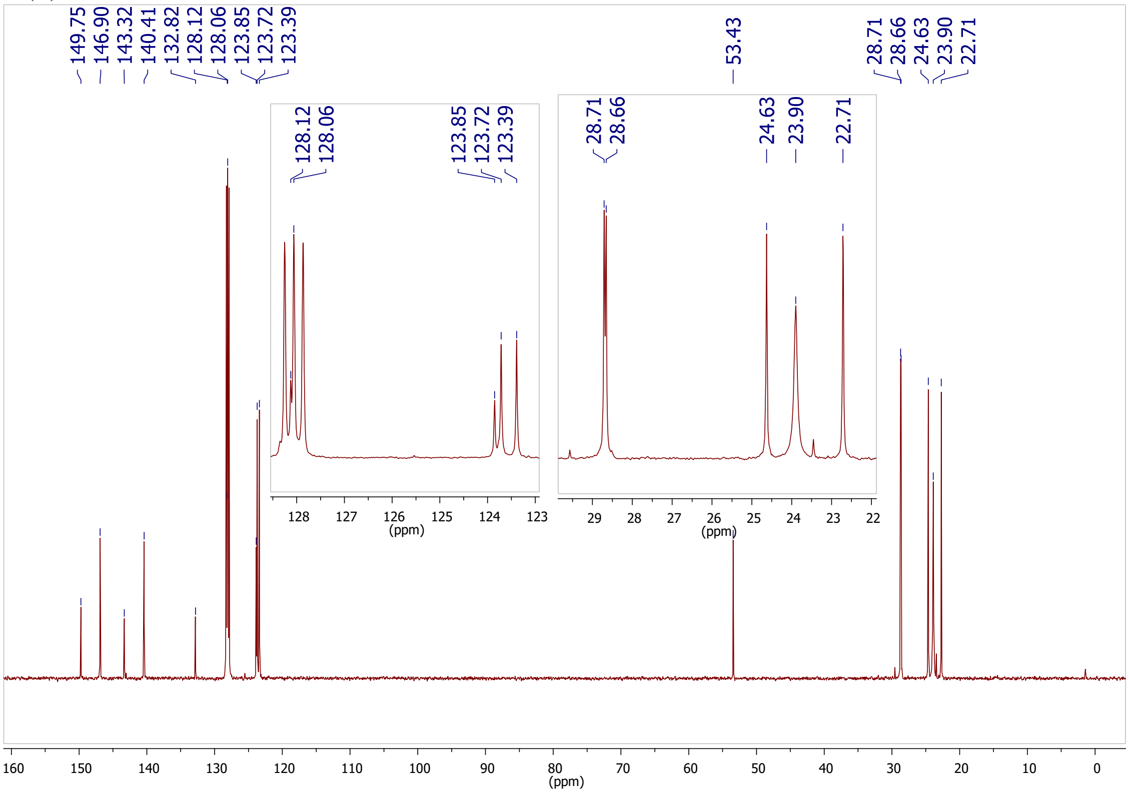

Figure S27. ^{1}H (a) and ^{13}C{^{1}H} (b) NMR in C_6D_6 of compound **5a** (isolated product).

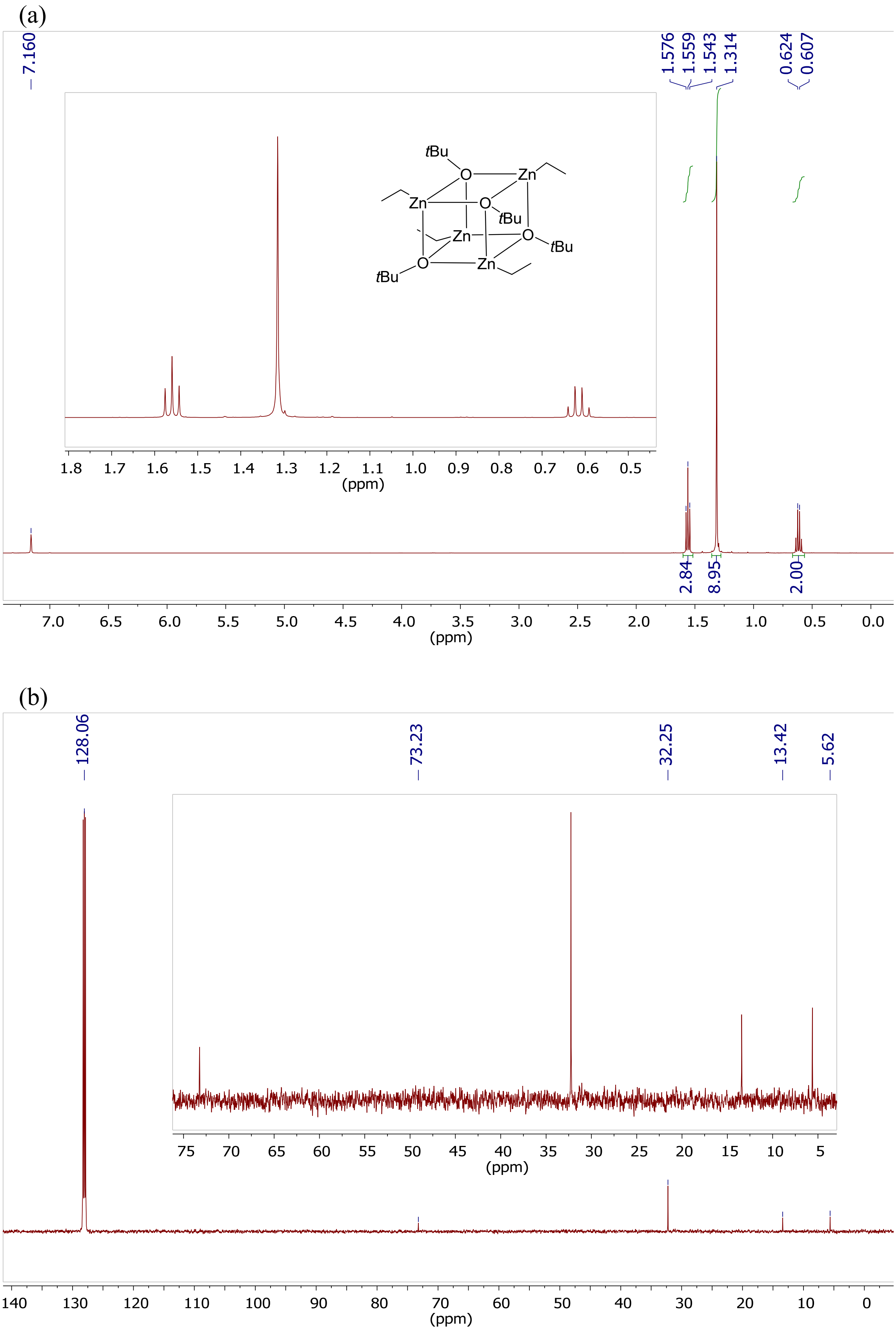

Figure S28. ^1H (a) and $^{13}C\{^1H\}$ (b) NMR in C_6D_6 of compound **6**.

Kinetic studies. All kinetics experiments were performed in a similar fashion. A measured amount of DTC was dissolved in C_6D_6. Then, *t*BuOH (neat) and the internal standard TKS (50.0 μL of a 0.2 M stock solution, 0.01 mmol) were added and charged to a J. Young NMR tube. Finally, the appropriate amount of a $ZnEt_2$ solution in hexane (1 M) was added and more C_6D_6 was added to reach a final volume of 0.8 mL. The NMR tube was then taken outside the glovebox and the sample was frozen in a cold acetone bath before starting the ^{1}H NMR experiments. All experiments were carried out by varying the concentration of one of the reagents (0.31 – 1.57 M) or catalyst (0.016 – 0.063 M) while keeping the others constant. The reaction progress was monitored by ^{1}H NMR every 20-30 s up to ca. 1 h, and product formation (**4d**) was measured integrating the NH signal (ca. 5.88 ppm) and/or the *t*Bu signal (ca. 1.59 ppm) vs. the internal standard. Reaction rates were determined by least-squares fit of initial product concentration vs. time. Plots of initial product concentration vs time for different initial concentrations of DTC, *t*BuOH and catalyst ($ZnEt_2$) are shown in Figures S29-31. Measuring initial reaction rates at different temperatures (Figure S32) allowed the calculation of activation parameters $\Delta H^{\ddagger}$, $\Delta S^{\ddagger}$ and E_a from the Eyring and the Arrhenius plots (Figures S33-S34). Labelling experiments were also carried out using *t*BuOD. The experiments were performed using [DTC] = [*t*BuOD] = 0.63 M and [cat] = 0.031 M at 298 K. Product formation was measured integrating the signal at 1.59 ppm owing to the *t*Bu group. Reaction rates were determined by least-squares fit of initial product concentration vs. time. The Kinetic Isotope Effect (KIE) was obtained by dividing the slopes obtained in these experiments by those obtained under the same conditions using *t*BuOH instead (Figure S35), to give a value of 2.6(5).

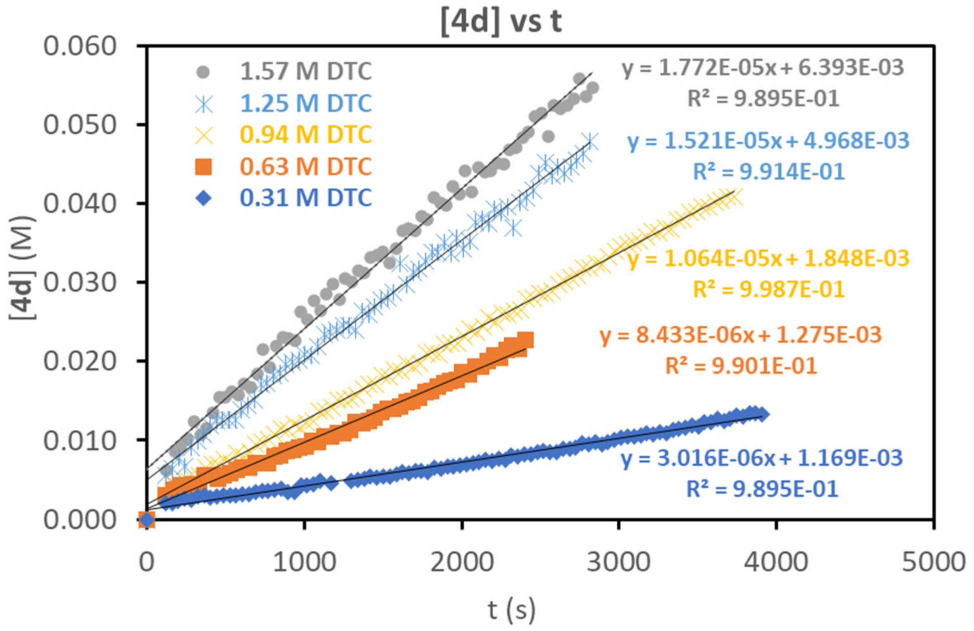

Figure S29. Plots of the initial concentration of **4d** vs. time varying DTC initial concentration (Reaction conditions: [DTC] = 0.31 M, 0.63 M, 0.94 M, 1.25 M, 1.57 M; [*t*BuOH] = 0.63 M; [$ZnEt_2$] = 0.016 M; T = 298 K)

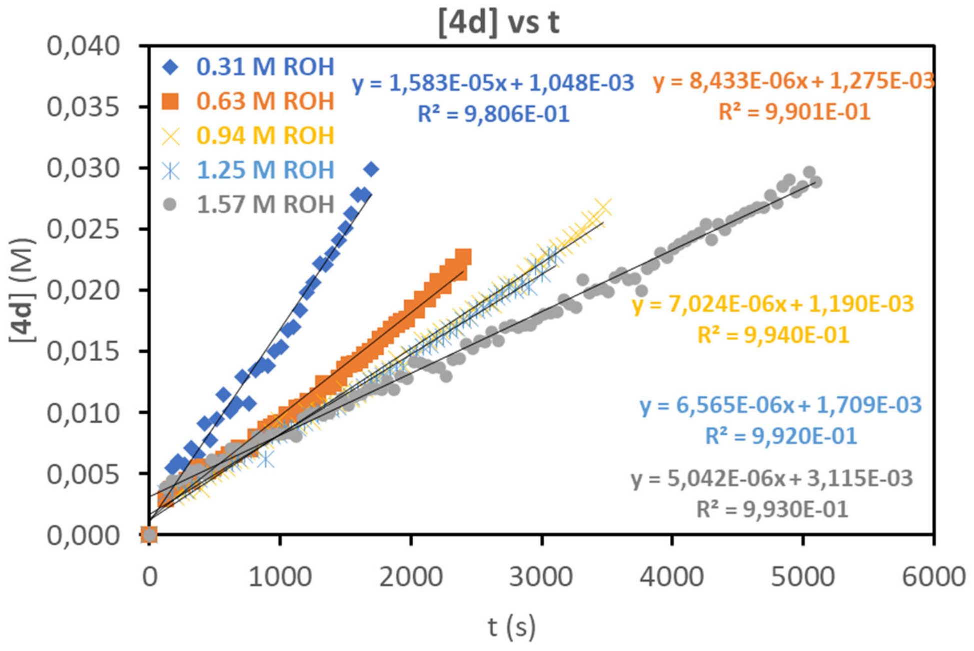

Figure S30. Plots of the initial concentration of **4d** vs. time varying *t*BuOH initial concentration (Reaction conditions: [*t*BuOH] = 0.31 M, 0.63 M, 0.94 M, 1.25 M, 1.57 M; [DTC] = 0.63 M; [$ZnEt_2$] = 0.016 M; T = 298 K).

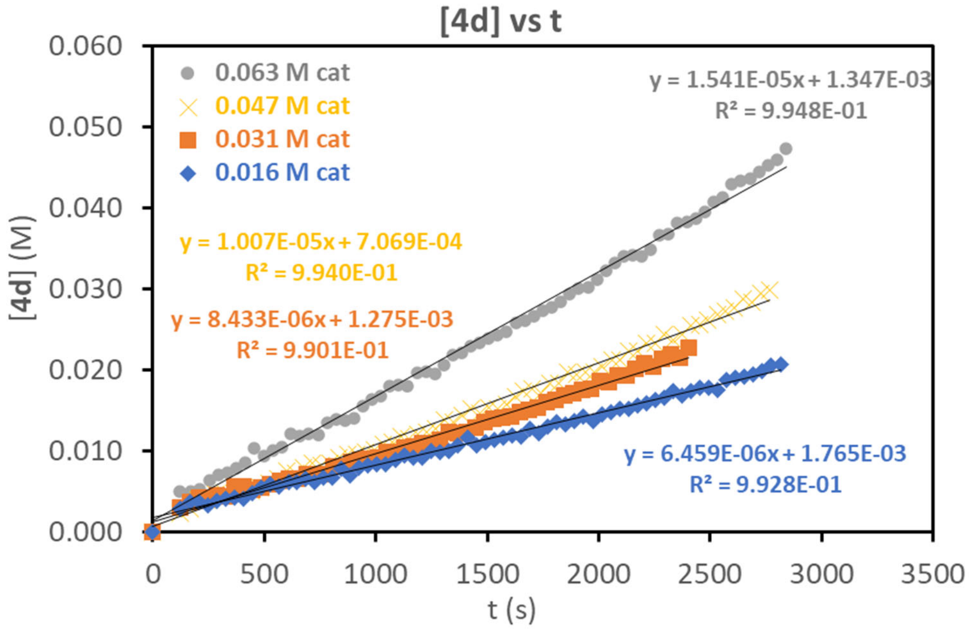

Figure S31. Plots of the initial concentration of **4d** vs. time varying catalyst initial concentration (Reaction conditions: [*t*BuOH] = 0.63 M; [DTC] = 0.63 M; [$ZnEt_2$] = 0.016 M. 0.031 M, 0.047 M, 0.063 M; T = 298 K).

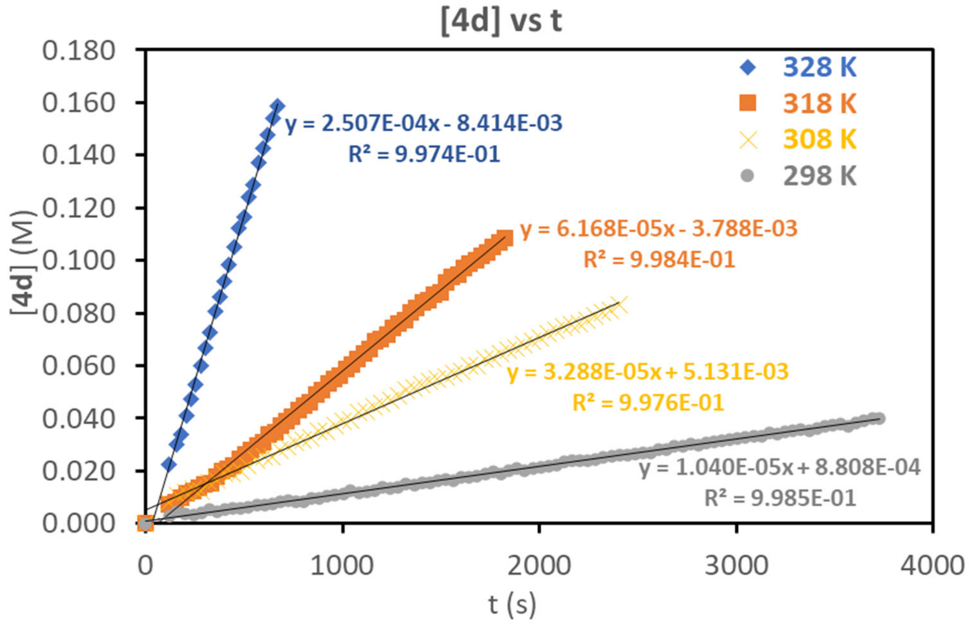

Figure S32. Plots of the initial concentration of **4d** vs. time varying reaction temperature (Reaction conditions: [*t*BuOH] = 0.63 M; [DTC] = 0.63 M; [$ZnEt_2$] = 0.031 M; T = 298 K, 308 K, 318 K, 328 K).

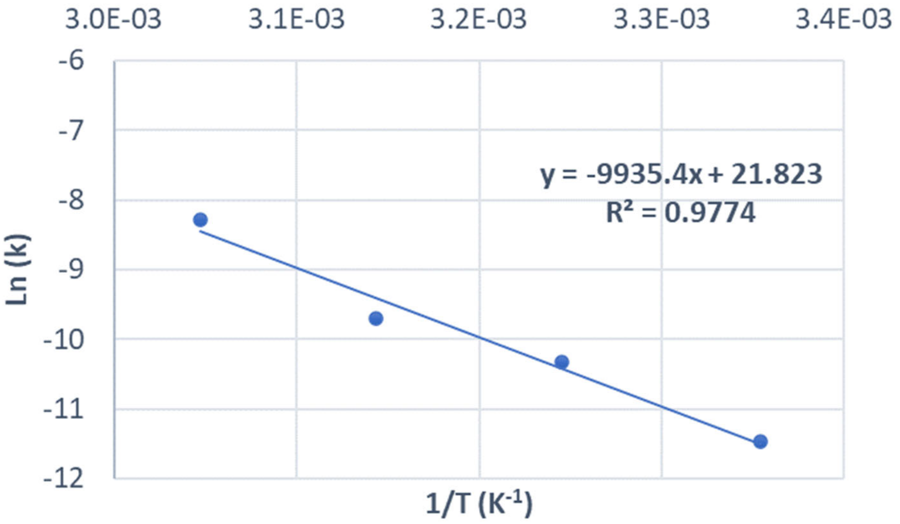

Figure S33. Arrhenius plot (ln k vs 1/T) (Reaction conditions: [*t*BuOH] = 0.63 M; [DTC] = 0.63 M; [$ZnEt_2$] = 0.031 M; T = 298 K, 308 K, 318 K, 328 K).

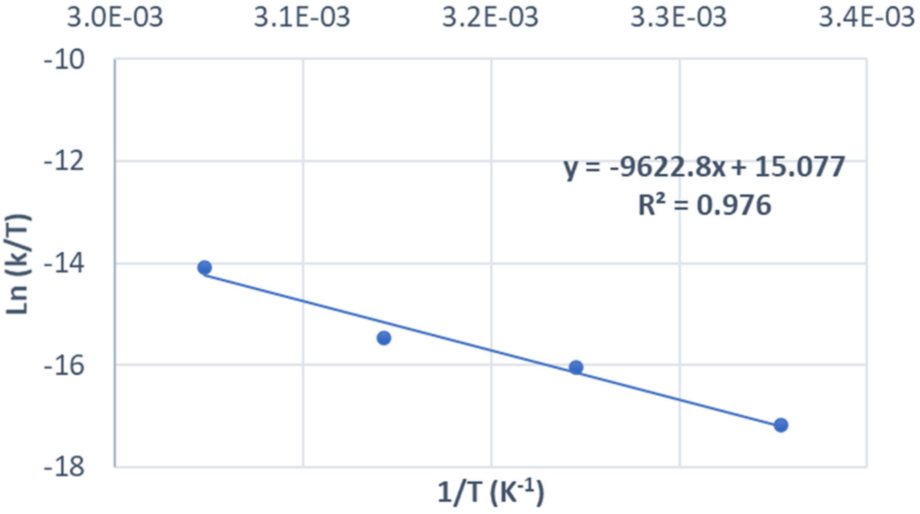

Figure S34. Eyring plot (ln k/T vs 1/T) (Reaction conditions: [*t*BuOH] = 0.63 M; [DTC] = 0.63 M; [$ZnEt_2$] = 0.031 M; T = 298 K, 308 K, 318 K, 328 K).

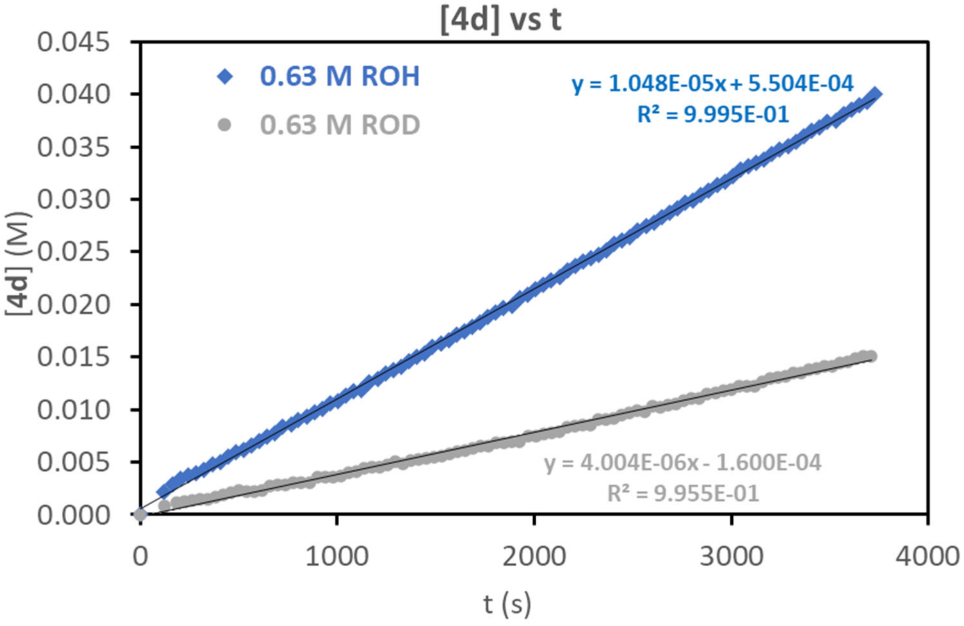

Figure S35. Comparing slopes in plots of the initial concentration of **4d** vs. time using *t*BuOD or *t*BuOH (Reaction conditions: [*t*BuOH] or [*t*BuOD] = 0.63 M; [DTC] = 0.63 M; [$ZnEt_2$] = 0.031 M; T = 298 K).

Stoichiometric experiments

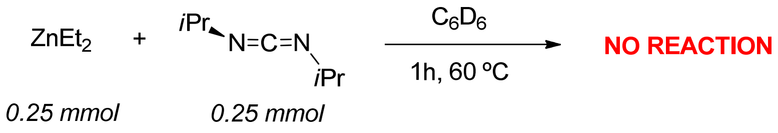

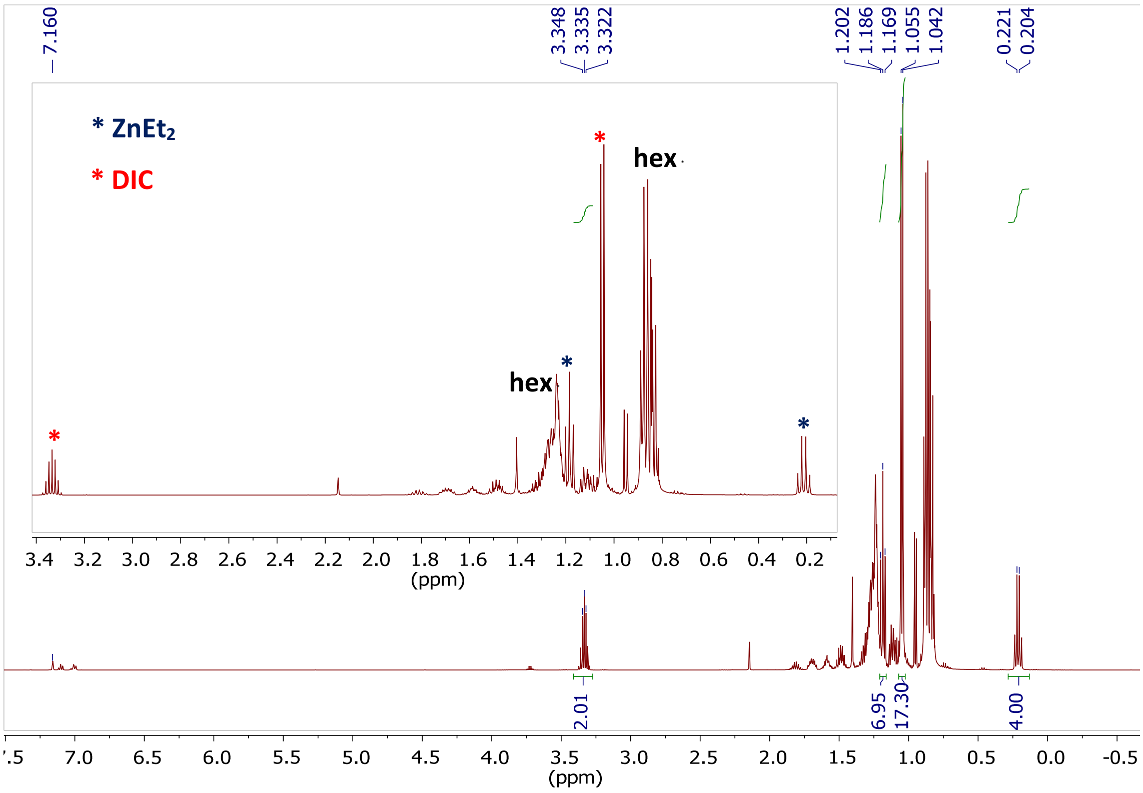

Figure S36. ^{1}H NMR in C_6D_6 for the stoichiometric reaction between $ZnEt_2$ and $C(N\textit{i}Pr)_2$ after 1h at 60 ºC.

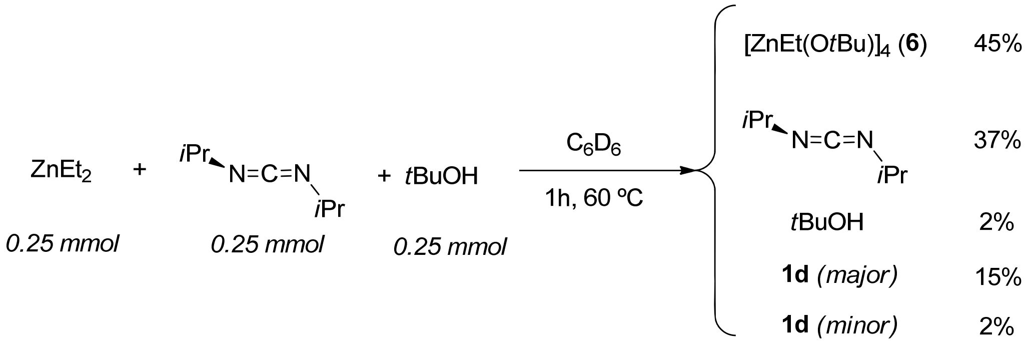

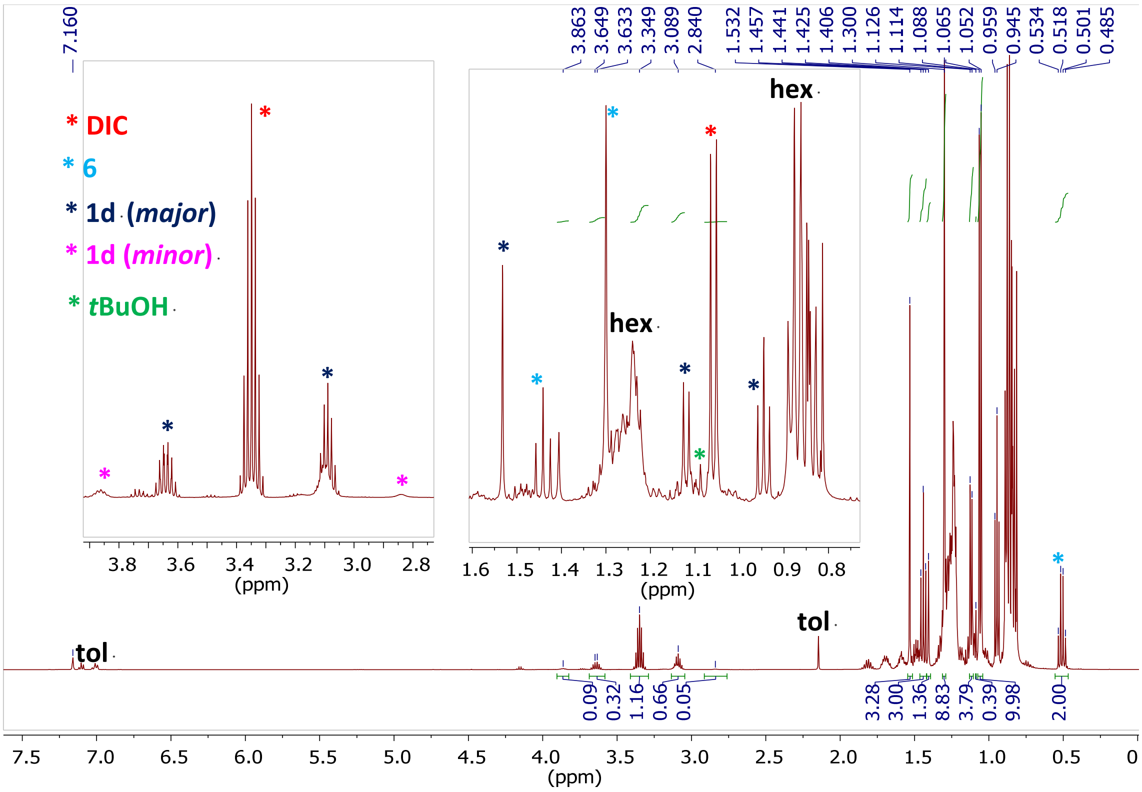

Figure S37. ^{1}H NMR in C_6D_6 for the stoichiometric reactions between $ZnEt_2$, $C(N\textit{i}Pr)_2$ and *t*BuOH after 1 h at 60 ºC.

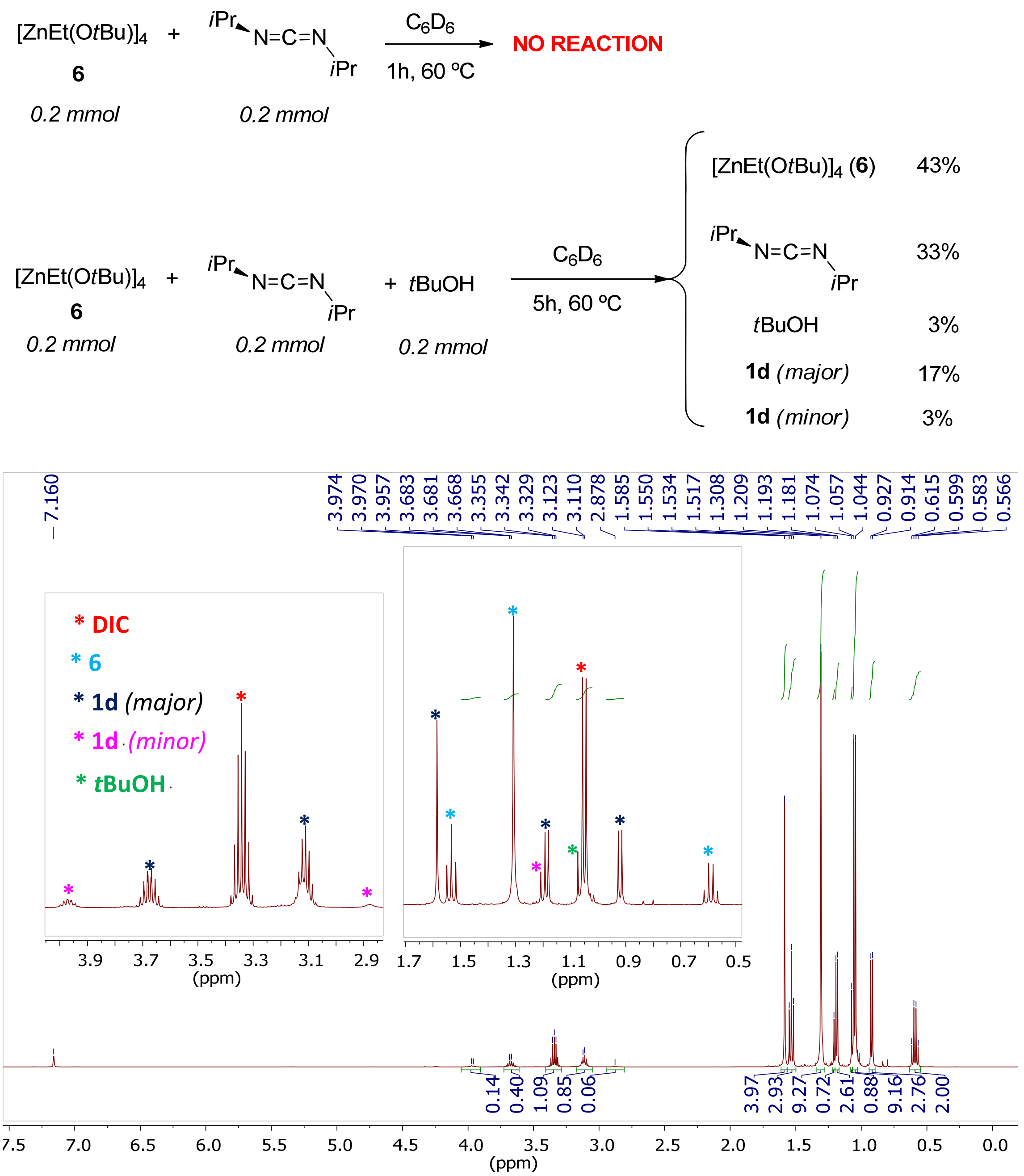

Figure S38. ^{1}H NMR in C_6D_6 for the stoichiometric reaction between [ZnEt(O*t*Bu)]$_4$ (**6**), C(N*i*Pr)$_2$ and *t*BuOH after 5 h at 60 ºC

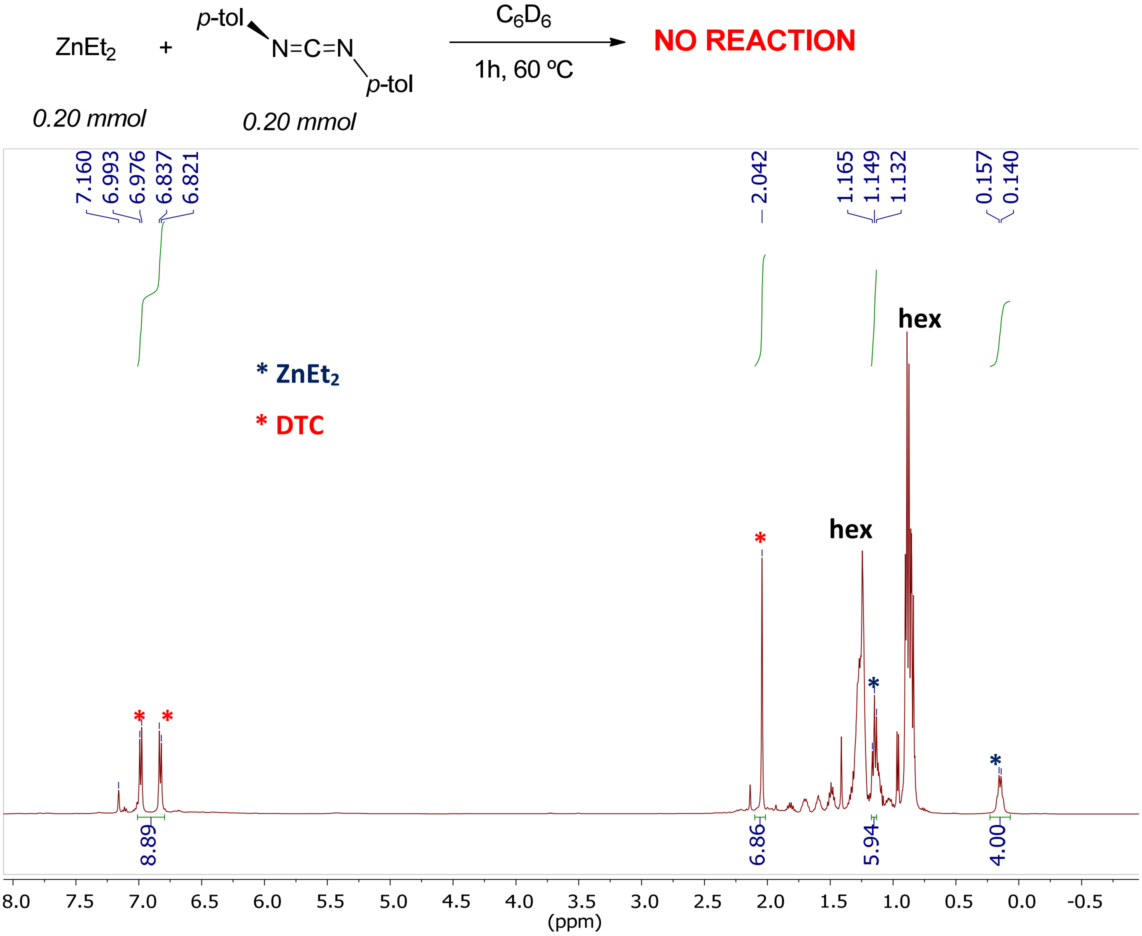

Figure S39. ^{1}H NMR in C_6D_6 for the stoichiometric reaction between $ZnEt_2$ and C(N*p*-tol)$_2$ after 1 h at 60 ºC.

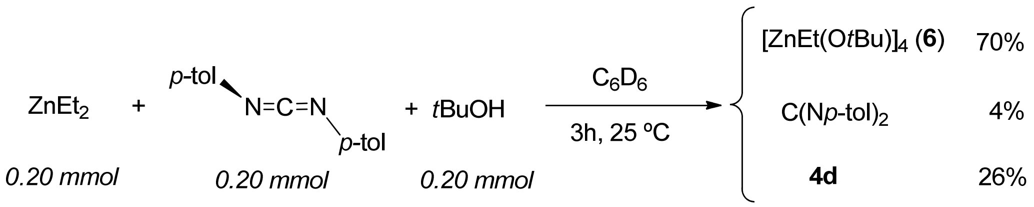

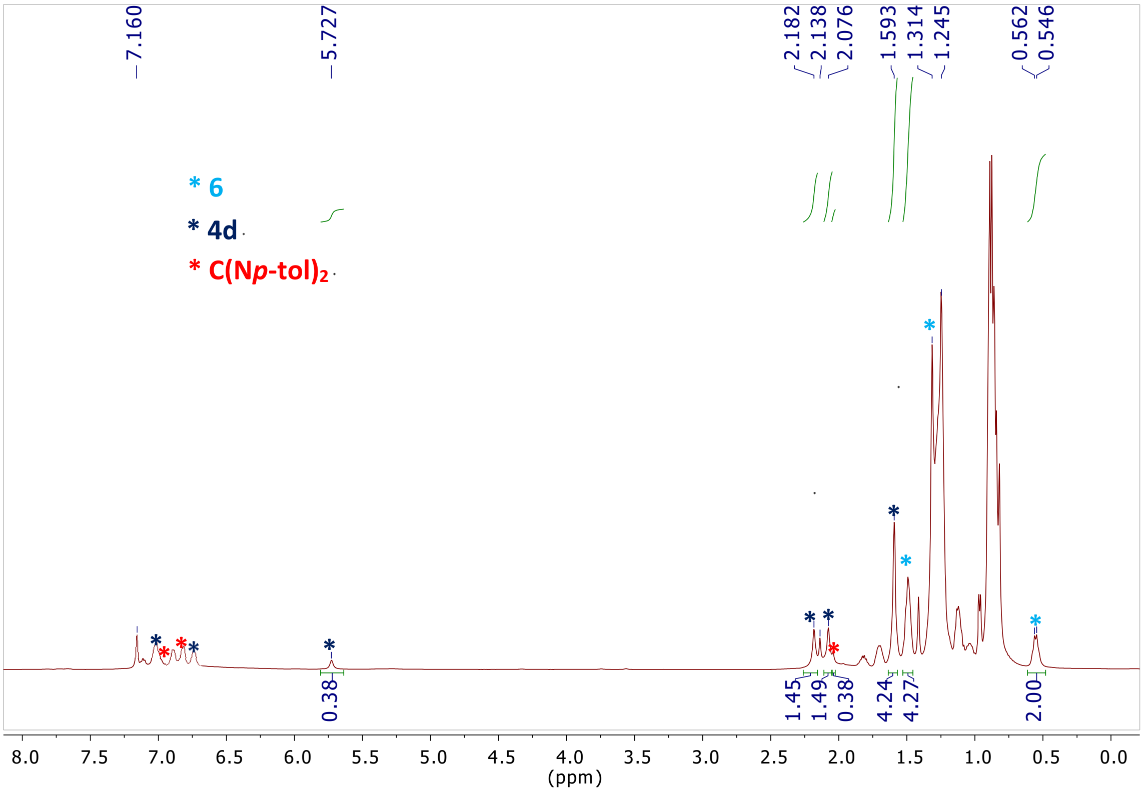

Figure S40. ^{1}H NMR in C_6D_6 for the stoichiometric reaction between $ZnEt_2$ and C(N*p*-tol)$_2$ and *t*BuOH after 3 h at 25 ºC.

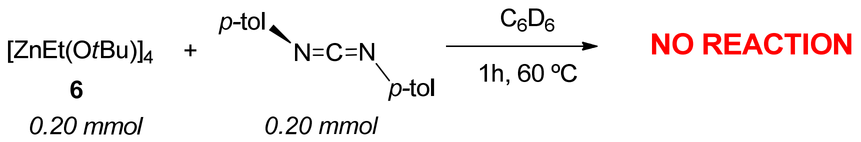

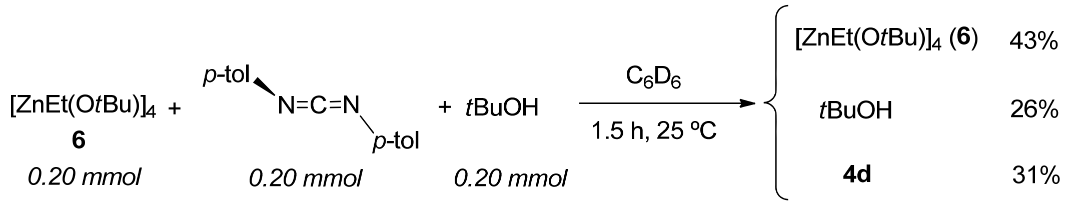

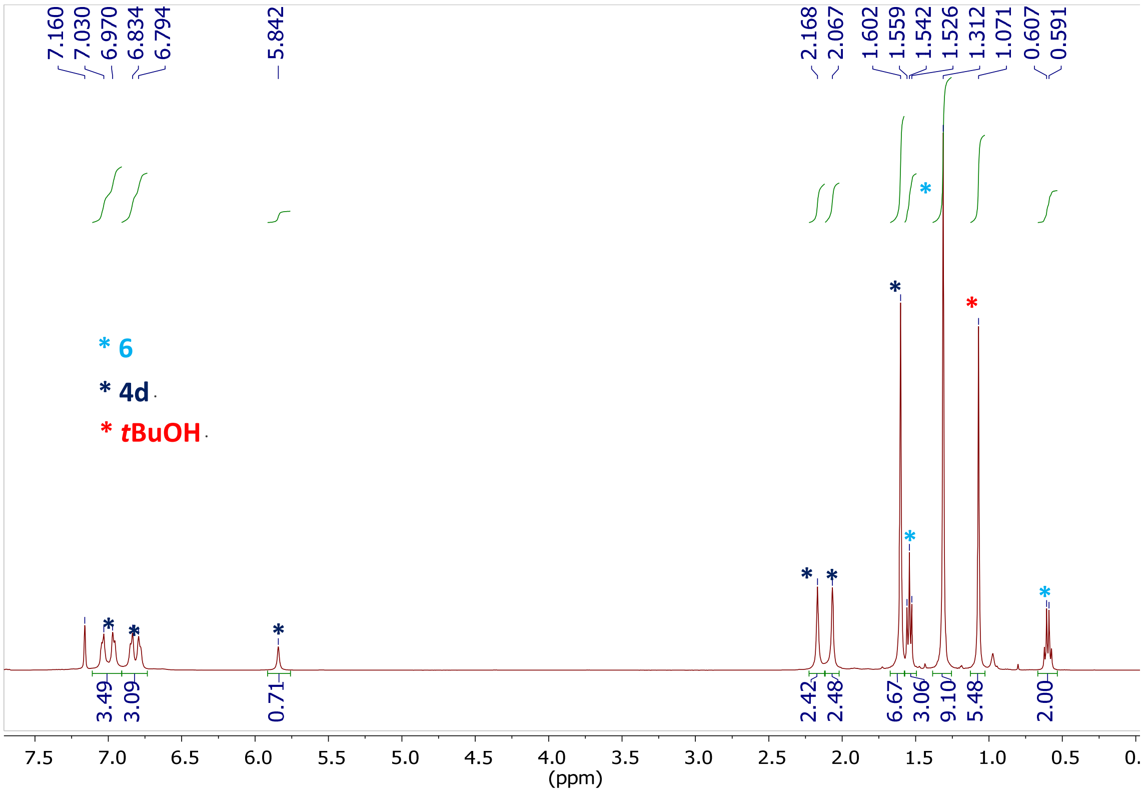

Figure S41. ^{1}H NMR in C_6D_6 for the stoichiometric reaction between [ZnEt(O*t*Bu)]$_4$ (**6**), C(N*p*-tol)$_2$ and *t*BuOH after 1.5 h at 25 ºC.

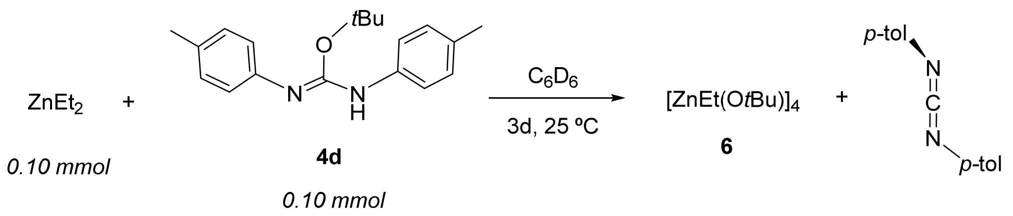

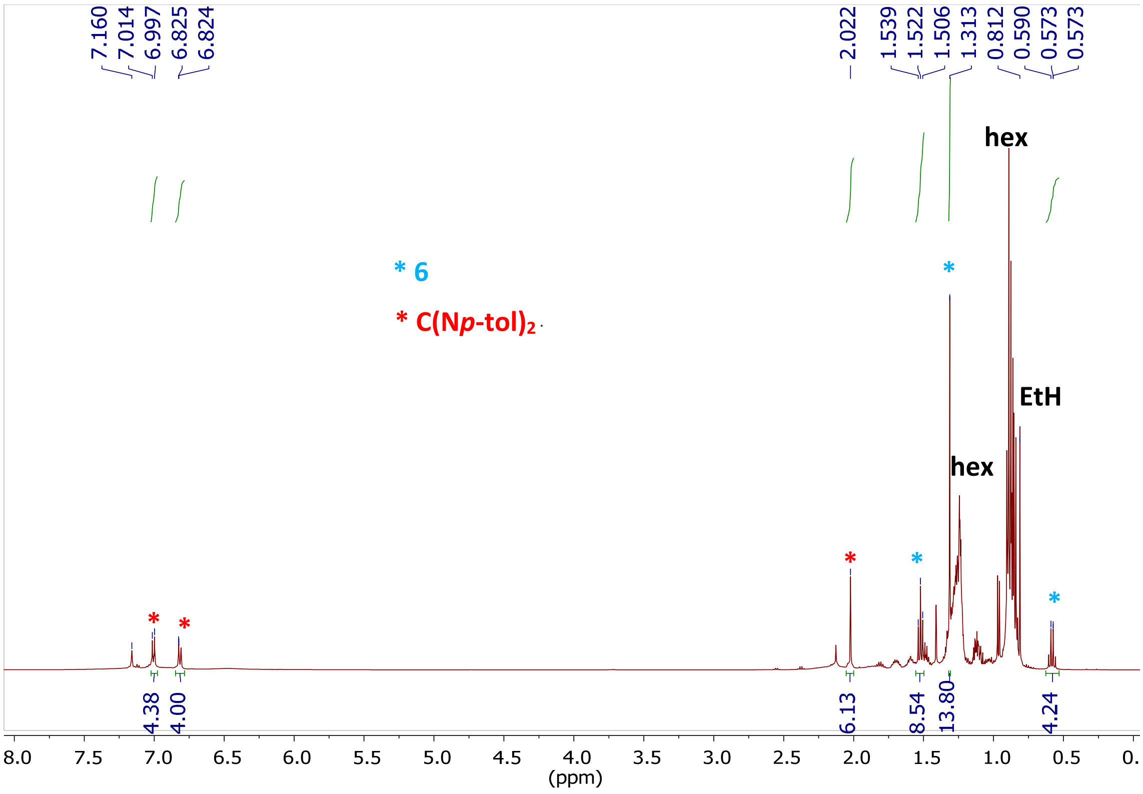

Figure S42. (a) ^{1}H NMR in C_6D_6 for the stoichiometric reaction between $ZnEt_2$ and **4d** after 3 days at 25 ºC.

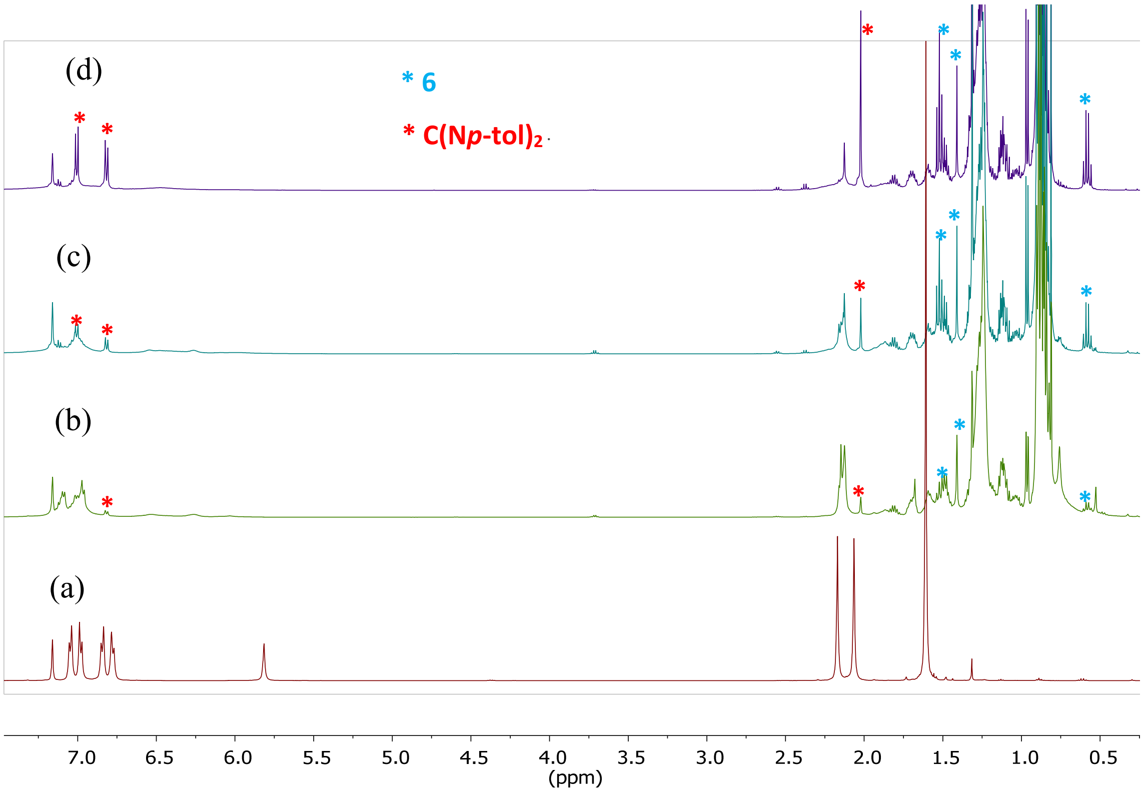

Figure S43. ^{1}H NMR stacked plot of reaction evolution of $ZnEt_2$ and **4d** in C_6D_6 at 25 ºC: (a) **4d**; (b) **4d** + $ZnEt_2$, 10 min, (c) **4d** + $ZnEt_2$, 2 h, (d) **4d** + $ZnEt_2$, 3 days.

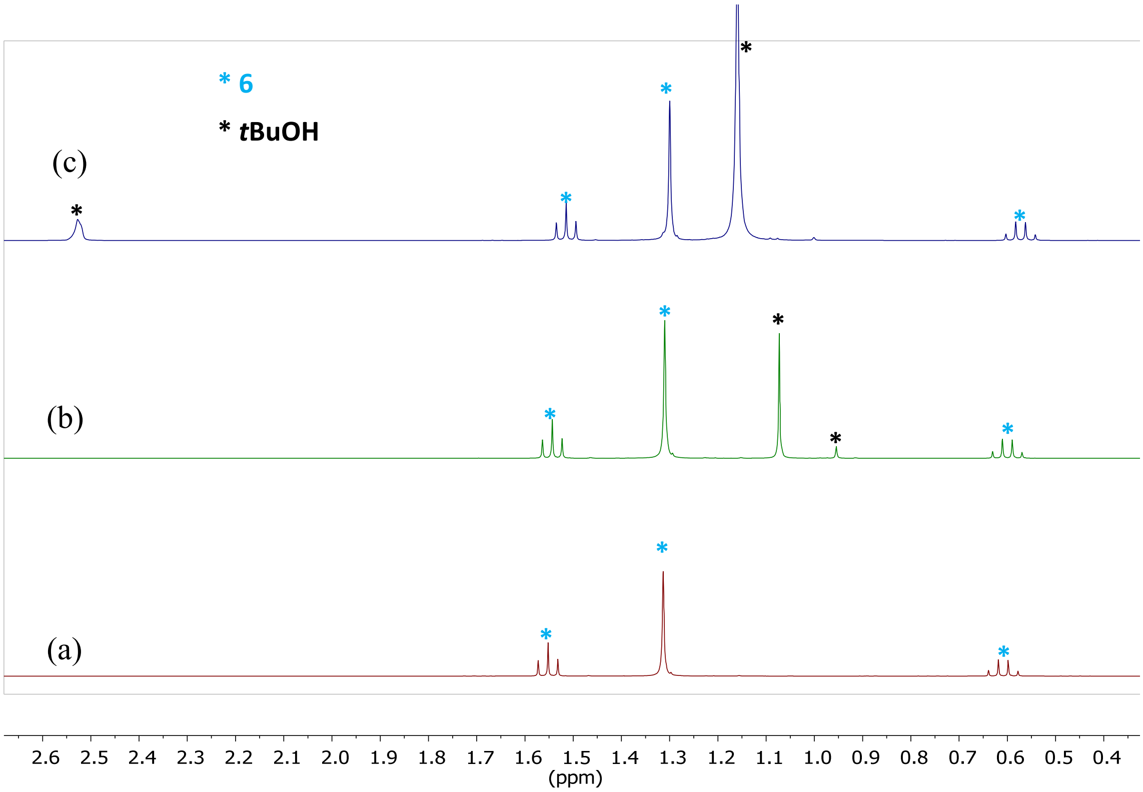

Figure S44. ^{1}H NMR stacked plot in C_6D_6 of: (a) $[ZnEt(O\textit{t}Bu)]_4$ (**6**, 0.20 mmol); (b) **6** + *t*BuOH (1 eq.); (c) **6** + *t*BuOH (5 eq.)

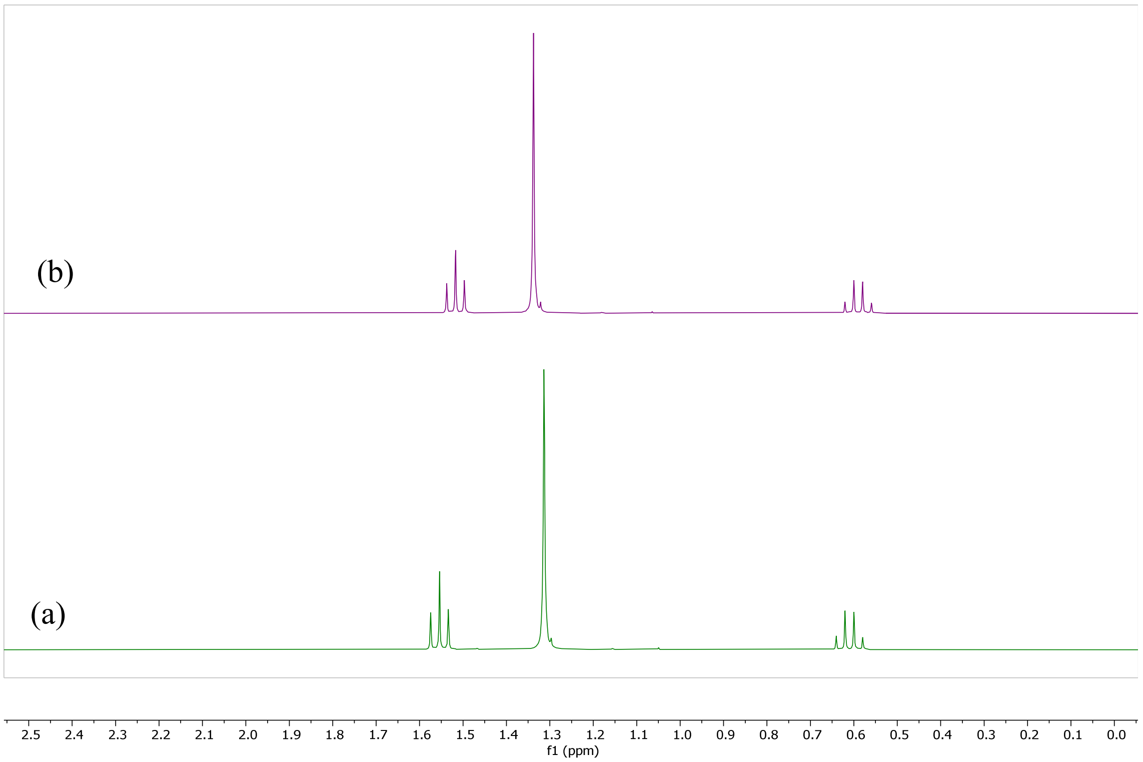

Figure S45. ^{1}H NMR of compound **6** in C_6D_6 at (a) 25 ºC and (b) 60 ºC.

X-ray crystal determination. X-ray data collection for compound **5a** was performed on a Bruker KAPPA series II diffractometer with APEX II area-detector system equipped with graphite monochromated Mo-Kα radiation (λ = 0.71073 Å). After data collection and integration with the Bruker SAINT software package,[13] absorption correction (SADABS)[14] were applied to the collected data as well as corrections for Lorentz and polarization effects. The structures were solved by SHELXT,[15] completed with different Fourier syntheses, and refined with full-matrix least-squares using SHELXL minimizing $\omega(Fo^2 - Fc^2)^2$,[16] using Olex 2.[17] Weighted R factors (Rw) and all goodness of fit S are based on F^2; conventional R factors (R) are based on F. All non-hydrogen atoms were refined with anisotropic displacement parameters. The hydrogen atom positions were calculated geometrically and were allowed to ride on their parent carbon or nitrogen atoms with fixed isotropic U. All scattering factors and anomalous dispersion factors are contained in the SHELXTL 6.10 program library. Details of the structure determination and refinement of compounds are summarized in Table S2. Crystallographic data (excluding structure factors) for the structure reported in this paper has been deposited with the Cambridge Crystallographic Data Centre with deposition numbers CCDC 2172359.

Table S2. Crystallographic data and structure refinement details for compound **5a**.

Compound	5a
Chem. form.	C26 H38 N2 O
CCDC	2172359
Form. Weight	394.58
Cryst. system	monoclinic
Space group	$P\ 2_1/n$
a (Å)	10.5925(4)
b (Å)	12.1575(5)
c (Å)	20.1117(8)
α (°)	90
β (°)	98.1100(10)
γ (°)	90
V (Å^3)	2564.05(18)
Z	4
GOF[a]	1.057
R_{int}	0.0398
R_1[b] / wR_2[c] [I > 2σ(I)]	0.0465 / 0.1238 [d]
R_1[b] / wR_2[c] [all data]	0.0806 / 0.1451

[a] $S = [\Sigma w(F_0^2 - F_c^2)^2 / (N_{obs} - N_{param})]^{1/2}$, $w = 1/[\sigma^2(F_0^2) + (aP)^2 + bP]$ where $P = F_0^2 + 2F_c^2) / 3$. [b] $R_1 = \Sigma||F_0|-|F_c|| / \Sigma|F_0|$ [c] $wR_2 = [\Sigma w(F_0^2 - F_c^2)^2 / \Sigma wF_0^2]^{1/2}$. [d] a = 0.0740, b = 0.3260.

DFT calculations. All the calculations were carried out by using the Gaussian09 suite of programs[18] and the M06L functional.[19] A pruned numerical integration grid (99,590) was used for all the calculations via the keyword Int = Ultrafine. All the elements (N, O, C and H) were described with the 6-31G* basis.[20] Geometry optimizations were performed under no symmetry restrictions by using initial coordinates derived from the Xray data of related compounds. Frequency analysis was performed for all the stationary points to ensure that a minimum structure with no imaginary frequencies was achieved in each case.

From CH_3OH	
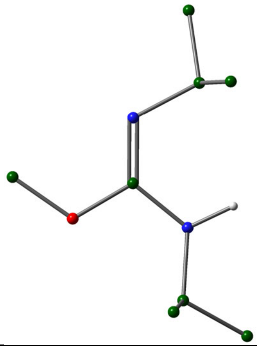	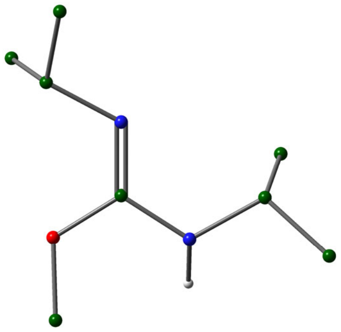
***anti*-1a**	***syn*-1a** (+3.3 kcal·mol^{-1})
From tBuOH	
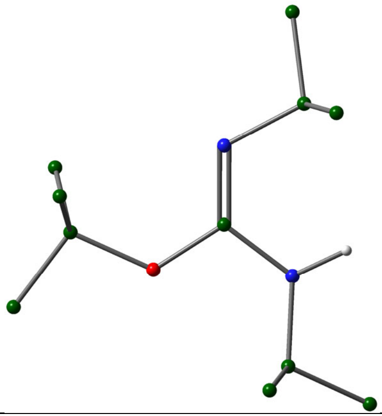	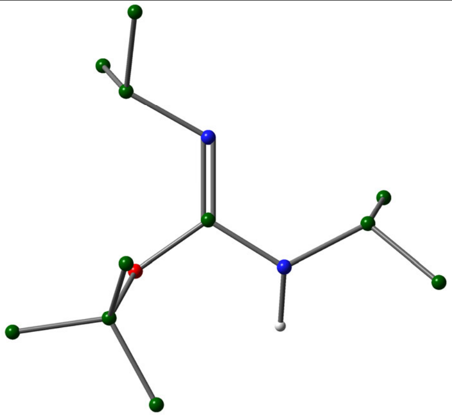
***anti*-1d**	***syn*-1d** (+1.7 kcal·mol^{-1})
From 1-adamantanol	
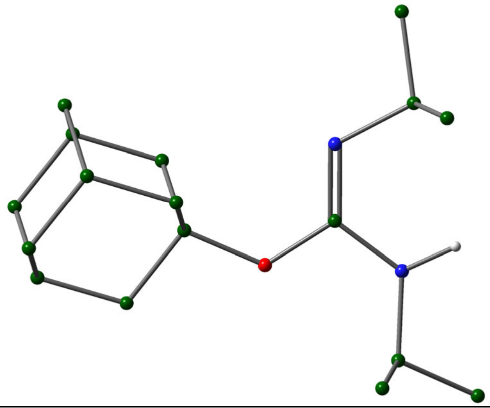	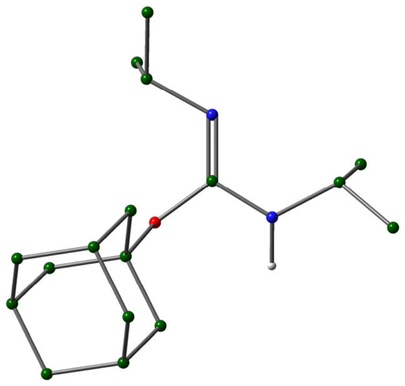
***anti*-1g**	***syn*-1g** (+0.7 kcal·mol^{-1})

<table>
<tr><td colspan="2">From 2,6-$Me_2C_5H_3OH$</td></tr>
<tr><td>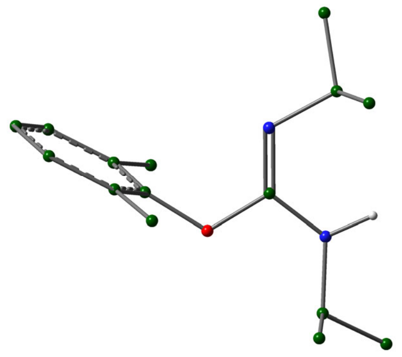</td><td>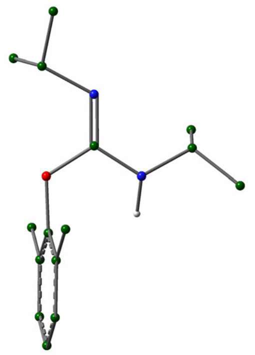</td></tr>
<tr><td>anti-1i (+0.6 kcal·mol^{-1})</td><td>syn-1i</td></tr>
<tr><td colspan="2">From 2,6-$tBu_2C_5H_3OH$</td></tr>
<tr><td>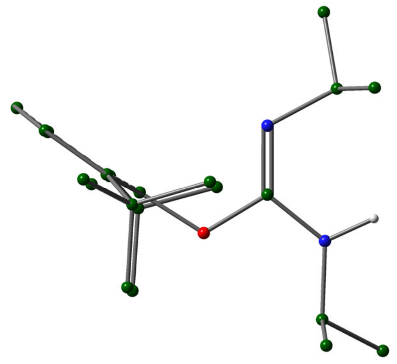</td><td>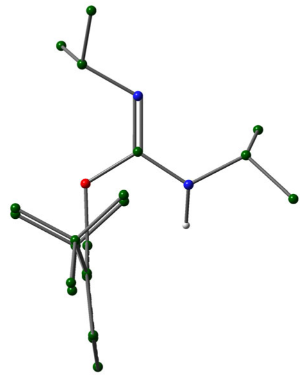</td></tr>
<tr><td>anti-1j (+2.2 kcal·mol^{-1})</td><td>syn-1j</td></tr>
<tr><td colspan="2">From CH_3OH</td></tr>
<tr><td>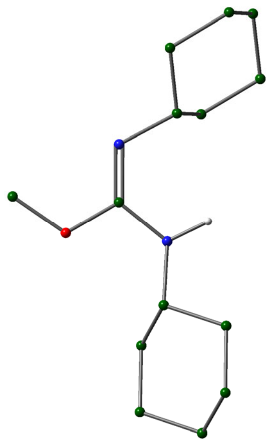</td><td>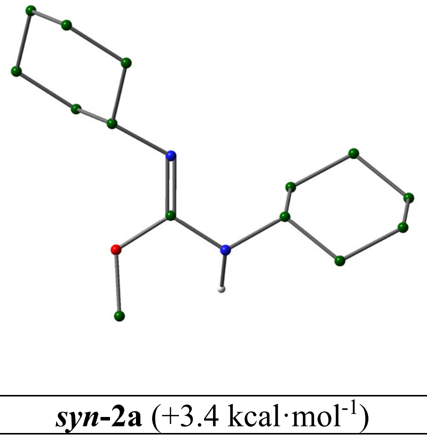</td></tr>
<tr><td>anti-2a</td><td>syn-2a (+3.4 kcal·mol^{-1})</td></tr>
<tr><td colspan="2">From CH_3OH</td></tr>
<tr><td>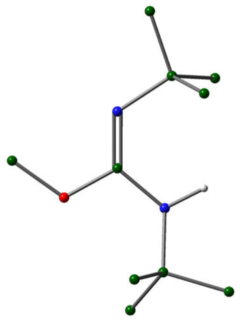</td><td>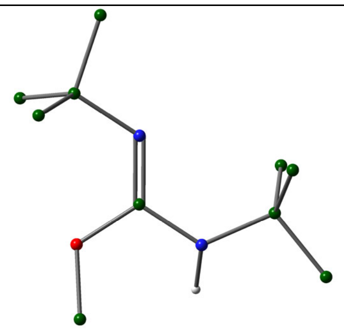</td></tr>
<tr><td>anti-3a</td><td>syn-3a (+2.1 kcal·mol^{-1})</td></tr>
</table>

<table>
<tr><th colspan="2">From CH_3OH</th></tr>
<tr><td>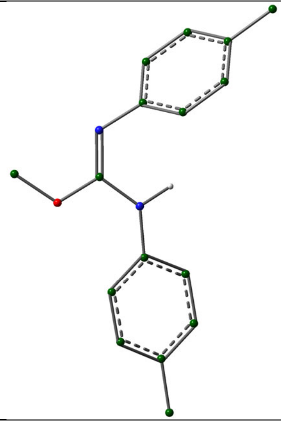</td><td>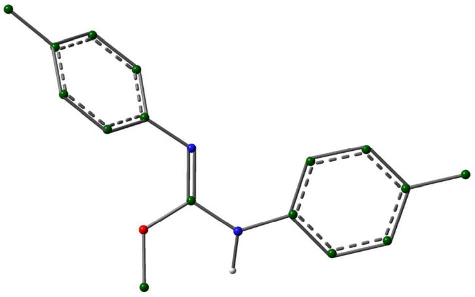</td></tr>
<tr><td>anti-4a</td><td>syn-4a (+5.6 kcal·mol^{-1})</td></tr>
<tr><th colspan="2">From tBuOH</th></tr>
<tr><td>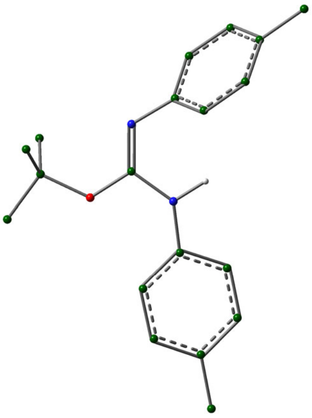</td><td>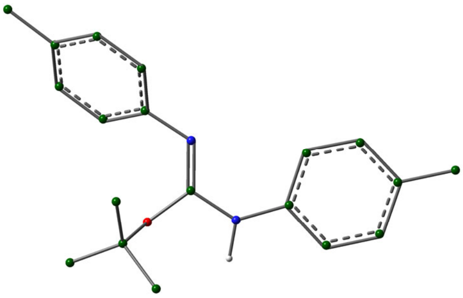</td></tr>
<tr><td>anti-4d</td><td>syn-4d (+5.5 kcal·mol^{-1})</td></tr>
<tr><th colspan="2">From 1-adamantanol</th></tr>
<tr><td>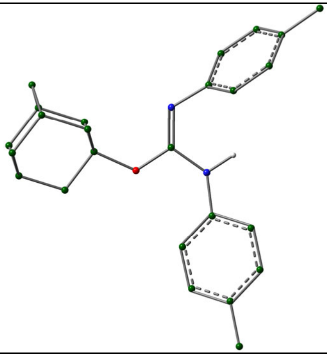</td><td>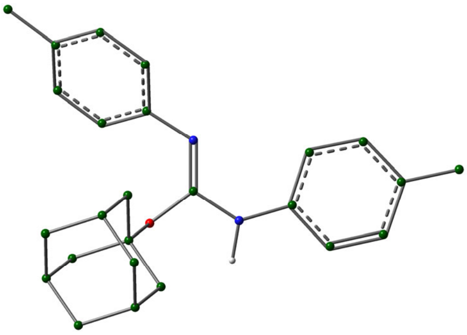</td></tr>
<tr><td>anti-4g</td><td>syn-4g (+2.9 kcal·mol^{-1})</td></tr>
<tr><th colspan="2">From 2,6-$Me_2C_5H_3OH$</th></tr>
<tr><td>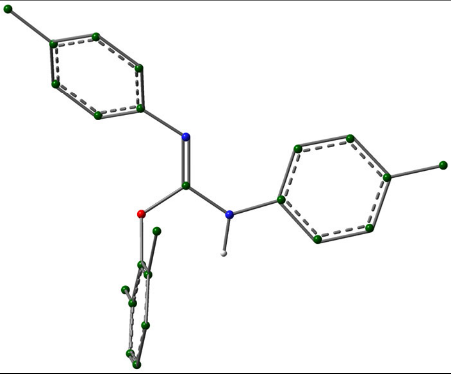</td><td>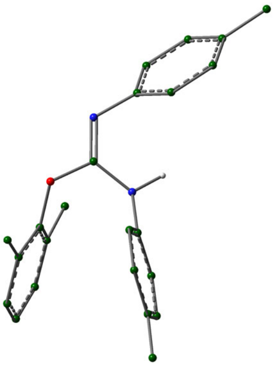</td></tr>
</table>

<table>
<tr><td>anti-4i</td><td>syn-4i (+0.5 kcal·mol^{-1})</td></tr>
<tr><td colspan="2">From 2,6-tBu$_2$C$_5$H$_3$OH</td></tr>
<tr><td>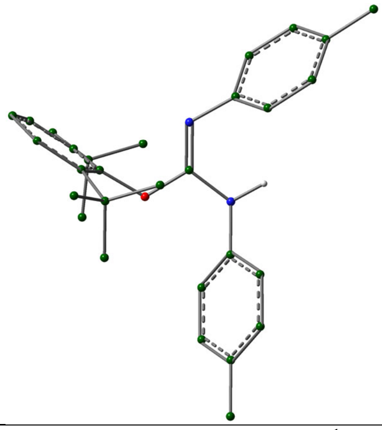</td><td>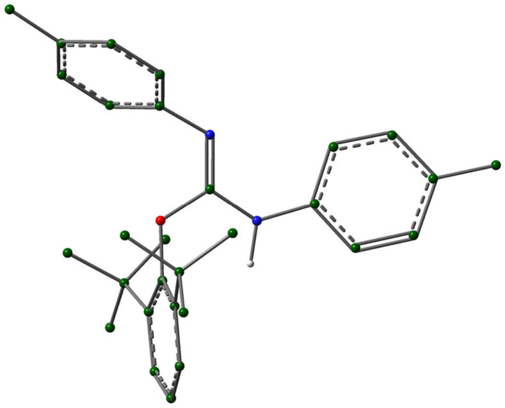</td></tr>
<tr><td>anti-4j (+3.2 kcal·mol^{-1})</td><td>syn-4j</td></tr>
<tr><td colspan="2">From CH$_3$OH</td></tr>
<tr><td>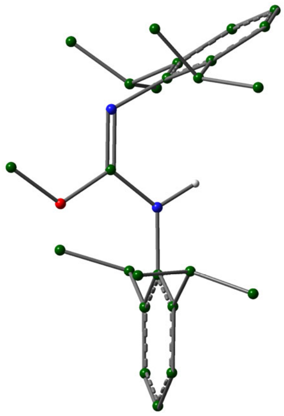</td><td>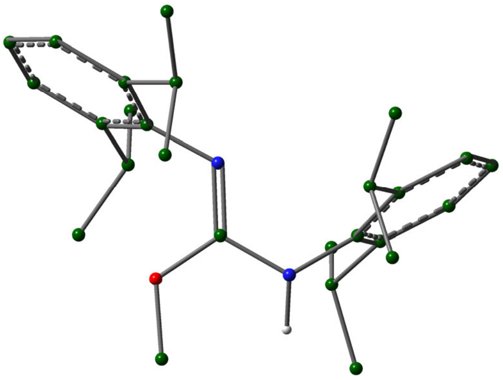</td></tr>
<tr><td>anti-5a</td><td>syn-5a (+7.9 kcal·mol^{-1})</td></tr>
</table>

REFERENCES

1. Melchor Bañales, A. J.; Larsen, M. B. Thermal Guanidine Metathesis for Covalent Adaptable Networks. *ACS Macro Lett.* **2020**, *9*, 937-943.

2. (a) Batrice, R. J.; Kefalidis, C. E.; Maron, L.; Eisen, M. S. Actinide-Catalyzed Intermolecular Addition of Alcohols to Carbodiimides. *J. Am. Chem. Soc.* **2016**, *138*, 2114-2117; (b) Liu, H.; Fridman, N.; Tamm, M.; Eisen, M. S. Catalytic Addition of Alcohols to Carbodiimides Mediated by Benzimidazolin-2-iminato Actinide Complexes. *Organometallics* **2017**, *36*, 4600-4610; (c) Imberdis, A.; Lefèvre, G.; Thuéry, P.; Cantat, T. Metal-Free and Alkali-Metal-Catalyzed Synthesis of Isoureas from Alcohols and Carbodiimides. *Angew. Chem. Int. Ed.* **2018**, *57*, 3084-3088.

3. Bhattacharjee, J.; Harinath, A.; Banerjee, I.; Nayek, H. P.; Panda, T. K. Highly Active Dinuclear Titanium(IV) Complexes for the Catalytic Formation of a Carbon–Heteroatom Bond. *Inorg. Chem.* **2018**, *57*, 12610-12623.

4. (a) West, K. R.; Bake, K. D.; Otto, S. Dynamic Combinatorial Libraries of Disulfide Cages in Water. *Org. Lett.* **2005**, *7*, 2615-2618; (b) Tietze, L. F.; Brasche, G.; Grube, A.; Bohnke, N.; Stadler, C. Synthesis of novel spinosyn A analogues by Pd-mediated transformations. *Chem. Eur. J.* **2007**, *13*, 8543-8563.

5. Hu, L.; Lu, C.; Zhao, B.; Yao, Y. Intermolecular addition of alcohols to carbodiimides catalyzed by rare-earth metal amides. *Org. Chem. Front.* **2018**, *5*, 905-908.

6. Tate, J. A.; Hodges, G.; Lloyd-Jones, G. C. O-Phenylisourea Synthesis and Deprotonation: Carbodiimide Elimination Precludes the Reported Chapman Rearrangement. *Eur. J. Org. Chem.* **2016**, *2016*, 2821-2827.

7. Badache, L.; Rahal, S.; Ghosez, L. Synthesis and characterization of a series of O-alkyl-N,N'-dialkylisoureas. *J. Soc. Alger. Chim.* **2002**, *12*, 11-19.

8. (a) Liu, H.; Khononov, M.; Fridman, N.; Tamm, M.; Eisen, M. S. Catalytic Addition of Alcohols into Carbodiimides Promoted by Organoactinide Complexes. *Inorg. Chem.* **2017**, *56*, 3153-3157. (b) Khononov, M.; Liu, H.; Fridman, N.; Tamm, M.; Eisen, M. S. Benzimidazolin-2-iminato Hafnium Complexes: Synthesis, Characterization, and Catalytic Addition of Alcohols to Carbodiimides. *Organometallics* **2020**, *39*, 3021-3033.

9. Liu, H.; Fridman, N.; Tamm, M.; Eisen, M. S. Addition of E–H (E = N, P, C, O, S) Bonds to Heterocumulenes Catalyzed by Benzimidazolin-2-iminato Actinide Complexes. *Organometallics* **2017**, *36*, 3896-3903.

10. Ghatak, T.; Fridman, N.; Eisen, M. S. Actinide Complexes Possessing Six-Membered N-Heterocyclic Iminato Moieties: Synthesis and Reactivity. *Organometallics* **2017**, *36*, 1296-1302.

11. Minamisono, K.; Suzuki, M.; Yoshimura, M.; Kuroda, T. Isourea derivatives for reducing terminal carboxyl concentration in polyesters. JP07145139A, 1995.

12. (a) Coates, G. E.; Ridley, D. 341. Alkoxy-, thio-, and amino-derivatives of methylzinc. *J. Chem. Soc.* **1965**, 1870-1877; (b) Yutaka, M.; Kazuhide, K.; Masao, N.; Yujiro, T. The Crystal Structure of Ethylzinc t-Butoxide. *Bull. Chem. Soc. Jpn.* **1966**, *39*, 1828-1828.

13. SAINT+NT Version 6.04, SAX Area-Detector Integration Program, Bruker Analytical X-ray Instruments, Madison, WI, 1997–2001.

14. Sheldrick, G. M. *SADABS, Program for Empirical Adsorption Correction*, Institute for Inorganic Chemistry, University of Gottingen: Germany, 1996.

15. Sheldrick, G. SHELXT - Integrated space-group and crystal-structure determination. *Acta Crystallogr. Sect. A: Found. Crystallogr.* **2015**, *71*, 3-8.

16. Sheldrick, G. Crystal structure refinement with SHELXL. *Acta Crystallogr. Sect. C: Cryst. Struct. Commun.* **2015**, *71*, 3-8.

17. Dolomanov, O. V.; Bourhis, L. J.; Gildea, R. J.; Howard, J. A. K.; Puschmann, H. OLEX2: a complete structure solution, refinement and analysis program. *J. Appl. Crystallogr.* **2009**, *42*, 339-341.

18. Frisch, M. J.; Trucks, G. W.; Schlegel, H. B.; Scuseria, G. E.; Robb, M. A.; Cheeseman, J. R.; Scalmani, G.; Barone, V.; Mennucci, B.; Petersson, G. A.; Nakatsuji, H.; Caricato, M.; Li, X.; Hratchian, H. P.; Izmaylov, A. F.; Bloino, J.; Zheng, G.; Sonnenberg, J. L.; Hada, M.; Ehara, M.; Toyota, K.; Fukuda, R.; Hasegawa, J.; Ishida, M.; Nakajima, T.; Honda, Y.; Kitao, O.; Nakai, H.; Vreven, T.; Montgomery, J. A.; Peralta, J. E.; Ogliaro, F.; Bearpark, M.; Heyd, J. J.; Brothers, E.; Kudin, K. N.; Staroverov, V. N.; Kobayashi, R.; Normand, J.; Raghavachari, K.; Rendell, A.; Burant, J. C.; Iyengar, S. S.; Tomasi, J.; Cossi, M.; Rega, N.; Millam, J. M.; Klene, M.; Knox, J. E.; Cross, J. B.; Bakken, V.; Adamo, C.; Jaramillo, J.; Gomperts, R.; Stratmann, R. E.; Yazyev, O.; Austin, A. J.; Cammi, R.; Pomelli, C.; Ochterski, J. W.; Martin, R. L.; Morokuma, K.; Zakrzewski, V. G.; Voth, G. A.; Salvador, P.; Dannenberg, J. J.; Dapprich, S.; Daniels, A. D.; Farkas; Foresman, J. B.; Ortiz, J. V.; Cioslowski, J.; Fox, D. J., Gaussian 09, Revision B.01. Wallingford CT, 2009.

19. Zhao, Y.; Truhlar, D. G. A new local density functional for main-group thermochemistry, transition metal bonding, thermochemical kinetics, and noncovalent interactions. *J. Chem. Phys.* **2006**, *125*, 194101.

20. (a) Hariharan, P. C.; Pople, J. A. The influence of polarization functions on molecular orbital hydrogenation energies. *Theor. Chim. Acta* **1973**, *28*, 213-222. (b Petersson, G. A.; Al-Laham, M. A. A complete basis set model chemistry. II. Open-shell systems and the total energies of the first-row atoms. *J. Chem. Phys.* **1991**, *94*, 6081-6090. (c) Petersson, G. A.; Bennett, A.; Tensfeldt, T. G.; Al-Laham, M. A.; Shirley, W. A.; Mantzaris, J. A complete basis set model chemistry. I. The total energies of closed-shell atoms and hydrides of the first-row elements. *J. Chem. Phys.* **1988**, *89*, 2193-2218.
